# Collective Oscillations in Coupled-Cell Systems

**DOI:** 10.1007/s11538-021-00883-7

**Published:** 2021-04-23

**Authors:** Kuan-Wei Chen, Chih-Wen Shih

**Affiliations:** Department of Applied Mathematics, National Yang Ming Chiao Tung University, National Chiao Tung University, Hsinchu, Taiwan 300

**Keywords:** Biological rhythm, Oscillation, Collective period, Average period, Hopf bifurcation

## Abstract

We investigate oscillations in coupled systems. The methodology is based on the Hopf bifurcation theorem and a condition extended from the Routh–Hurwitz criterion. Such a condition leads to locating the bifurcation values of the parameters. With such an approach, we analyze a single-cell system modeling the minimal genetic negative feedback loop and the coupled-cell system composed by these single-cell systems. We study the oscillatory properties for these systems and compare these properties between the model with Hill-type repression and the one with protein-sequestration-based repression. As the parameters move from the Hopf bifurcation value for single cells to the one for coupled cells, we compute the eigenvalues of the linearized systems to obtain the magnitude of the collective frequency when the periodic solution of the coupled-cell system is generated. Extending from this information on the parameter values, we further compute and compare the collective frequency for the coupled-cell system and the average frequency of the decoupled individual cells. To compare these scenarios with other biological oscillators, we perform parallel analysis and computations on a segmentation clock model.

## Introduction

Biological rhythms play important roles in nature. They include a wide variety of cyclical behaviors which are crucial in organisms, from development to homeostasis. The corresponding periodicity ranges from microseconds for cellular oscillations to seasons or years for recurring movements in ecological systems. Scientists have been continuously making concerted efforts to understand the mechanisms of synchrony, robustness, and sustainability of these oscillations, via mathematical and computational modeling, and experiments (Fall et al. 2002; Forger and Peskin 2003; Gonze 2011; Winfree 1980).

In mammals, a biological clock located in the suprachiasmatic nucleus (SCN) drives remarkably precise circadian rhythm. This master circadian clock is composed of multiple oscillator neurons that are coordinated through molecular regulation (Antle and Silver 2005; Bell-Pedersen et al. 2015). While individual cells oscillate with periods ranging from 20 to 28 hours, the collective rhythm in circadian clock is synchronized through intercellular coupling mediated by neurotransmitters. Experimental evidence shows that intercellular signal vasoactive intestinal polypeptide (VIP) expressed by some of the SCN neurons is such a major neurotransmitter (Aton et al. 2005). A special feature of the master circadian clock within the SCN is that the collective frequency of the synchronized coupled cells is close to the mean of the intrinsic frequencies of individual cells (Aton et al. 2005; Herzog et al. 2004; Honma et al. 2012; Liu et al. 1997; Taylor 2014).

The above-mentioned intricate biological mechanism involves some interesting features and evokes two issues in modeling: When a population of oscillatory cells is coupled, can a collective oscillation be produced? And will a collective oscillation be generated only under sufficiently large coupling strength? Herein, we consider that each individual cell oscillates at its own frequency when uncoupled. We call it a *collective oscillation* for these cells under coupling if a common frequency of oscillation is attained for all cells. Such common frequency was termed *compromise frequency* in a pair of phase equations in Strogatz (1994). Therein, such a collective oscillation is generated if the coupling strength is larger than half of the difference between the two individual frequencies. Herein, for generality, we call such common frequency of oscillation the *collective frequency* in the coupled-cell systems. The second issue is about the closeness between the collective frequency and the mean of the intrinsic frequencies of individual cells.

Denote by $\omega _i$ the individual frequency of the *i*th oscillator among the *N* oscillators, when isolated. Let us call it the *average frequency property* if the collective frequency of the coupled cells is equal to or close to the average frequency $\omega _{\mathrm{Ave}}$, where

1 $$\begin{aligned} \omega _{\mathrm{Ave}} := \frac{1}{N} \sum _{i=1}^{N} \omega _i. \end{aligned}$$

We note that average frequency is disparate from average period $T_{\mathrm{Ave}}$, and they were sometimes mixed up in the literature. The average period $T_{\mathrm{Ave}}$ is given by

2 $$\begin{aligned} T_{\mathrm{Ave}}: =\frac{1}{N} \sum _{i=1}^{N} T_i, \end{aligned}$$

where $T_i:=2\pi /\omega _i$ are the individual periods. For the average frequency $\omega _{\mathrm{Ave}}$ in (1), the corresponding period is

$$\begin{aligned} \frac{2\pi }{\omega _{\mathrm{Ave}}} = N/ \sum _{i=1}^{N} \frac{1}{T_i} \end{aligned}$$

which is not equal to $T_{\mathrm{Ave}}$ in (2) in general. Certainly, if all $\omega _i$ are close to each other, say $\omega _i \approx \omega$ for all $i=1, \ldots , N$, then the average frequency and the average period are about the same thing, as

$$\begin{aligned} \omega _{\mathrm{Ave}} \approx \omega , ~T_{\mathrm{Ave}}:= \frac{1}{N} \sum _{i=1}^NT_i \approx 2\pi /\omega \approx 2\pi /\omega _{\mathrm{Ave}}. \end{aligned}$$

A mathematical model which well depicts the average frequency property is the coupled phase equations:

3 $$\begin{aligned} {\dot{\theta }}_i = \omega _i + \sum _{j=1}^{N} K_{ji}f(\theta _j-\theta _i), \quad i=1,2,\ldots ,N, \end{aligned}$$

where $\theta _i$ is the oscillatory phase of cell *i*, $\omega _i$ is the intrinsic frequency of the *i*th cell, $K_{ji} \in {{\mathbb {R}}}$ is the connection weight, *f* is an interaction function, and *N* is the number of cells. System (3) is known as the Kuramoto model (Kuramoto 1984) and has been studied extensively (Chiba 2015; Ha et al. 2016). As pointed out in Liu et al. (1997), any phase-locked solution of (3) oscillates at the mean frequency $\sum _{i=1}^N \omega _i /N$, provided that *f* is an odd function and $[K_{ji}]$ is a symmetric matrix. This can be seen by adding up all components in (3). However, odd function and symmetric matrix are quite special among all possible interaction functions and connection matrices.

Phase equations are considered from focusing on the collective behavior in terms of the phase of oscillation in a collection of clock cells, instead of concentrating on the internal machinery of cells. However, it is significantly interesting to understand how oscillations are generated in cells. It has been identified that the intracellular transcriptional/translational negative feedback loops between activators and repressors are the key oscillatory mechanism in mammals and other organisms. Therefore, it is appealing to see whether the kinetic models based on such negative feedback loops accommodate the average frequency property.

Investigations on the average frequency property in some kinetic models have been reported in Gonze et al. (2005), Kim (2016), Kim et al. (2014). Recently, in a minimal genetic system constituted solely by a negative feedback loop, two types of gene regulation were studied and compared (Kim 2016; Kim et al. 2014). Therein, a single cell is modeled by

4 $$\begin{aligned} \left\{ \begin{aligned} \displaystyle {\dot{M}}&= \alpha _1 f(R)- \beta _1 M \\ \displaystyle {\dot{P}}&= \alpha _2 M - \beta _2 P \\ \displaystyle {\dot{R}}&= \alpha _3 P - \beta _3 R, \end{aligned} \right. \end{aligned}$$

where *M*, *P*, *R* are interpreted as the concentrations of a clock gene mRNA, the corresponding protein, and a transcriptional inhibitor, respectively, and the negative feedback loop is realized by the repression function *f*. The repression considered therein is either of Hill-function type or based on protein sequestration. System (4) is known as the Goodwin model, when the nonlinearity *f* is a Hill function (Goodwin 1965; Griffith 1968), i.e., $f=f_1$, where

5 $$\begin{aligned} f_1(R) := \frac{1}{1+(R/k_\mathrm{H})^n}, \end{aligned}$$

**Fig. 1:**
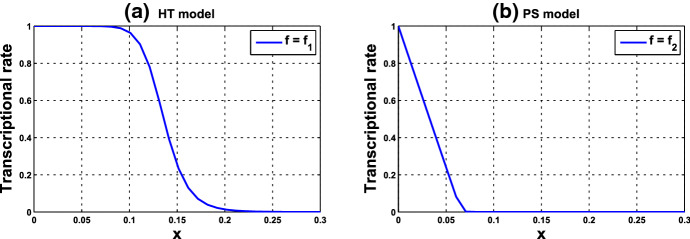
The transcription rate function **a** $f=f_1$, the Hill-function-type repression with $n=11$, $k_\mathrm{H}=0.136$, and **b** $f=f_2$, the protein-sequestration-based repression with $A=0.0659$, $k_d=0.00001$

$k_\mathrm{H} >0$ is the half-saturation constant, and positive integer *n* is the Hill coefficient. A graph of $f_1$ is depicted in Fig. 1a. The Goodwin model has been a prototypical system for accounting the core molecular mechanism associated with generation of self-sustained oscillations. It has been adopted to study circadian clocks (François et al. 2012; Ruoff et al. 1999, 2001). Hill functions were largely employed to describe cooperative binding of repressors to the gene promotor in transcription (Keller 1995) or repression based on multisite phosphorylation mechanism (Gonze and Abou-Jaoudé 2013). Hill-function-type repression has been widely adopted in various models for biological rhythms (Invernizzi and Treu 1991; Kim and Forger 2012; Kim 2016; Kurosawa et al. 2002; Kurosawa and Iwasa 2002, 2005).

Recently, another mechanism of transcriptional repression based on protein sequestration has been proposed and adopted into the negative feedback modeling. In such reaction, repressor protein *R* binds free activator *A* into inactive complex. The fraction of activators that are not sequestered is expressed by

6 $$\begin{aligned} f_2(R) := \frac{A-R-k_d+\sqrt{(A-R-k_d)^2+4A k_d}}{2A}, \end{aligned}$$

where $k_d>0$ is the dissociation constant between the activator and repressor (Buchler and Louis 2008; Buchler and Cross 2009; Kim 2016; Kim et al. 2014). A graph of $f_2$ is depicted in Fig. 1b. As transcription is in a ratio to this fraction, $f_2(R)$ was taken as *f*(*R*) in system (4) to depict the negative feedback loop. It was reported in Kim and Forger (2012) that tight binding between activators and repressors and balanced stoichiometry are the key for sustained rhythms in a detailed mathematical model on the mammalian circadian clock, and such property is also reflected in the simplified model, system (4) with $f=f_2$.

Goodwin’s model (system (4) with $f=f_1$) was proposed in Goodwin (1965). It was proved in Griffith (1968) that the equilibrium is stable for $n \le 8$, by the Routh–Hurwitz criterion. When $n >8$, it was shown that some parameter values can be found at which the equilibrium is unstable. In Kurosawa et al. Kurosawa et al. 2002, for a system similar to system (4), with $f=f_1$, it was shown that the equilibrium is stable for $n \le 8$, by the Routh–Hurwitz criterion, and for $n >8$, the equilibrium can be unstable for some specially chosen parameter values. In Fall et al. (2002), under the assumption of equal degradation rates, it was justified that the equilibrium is unstable if $n \ge 8$, if the degradation rates are small. We note that the steady state depends on the parameters, and so at the bifurcation value, the existence of equilibrium needs to be assured to confirm the occurrence of Hopf bifurcation. This was not addressed in those works. In Woller et al. (2014), under the assumption of equal degradation rates, the steady state at which the linearized system has a pair of purely imaginary eigenvalues was found, where the condition $n>8$ is explicitly revealed, yet, the crossing condition was not mentioned. In fact, $n>8$ is both a sufficient and necessary condition for the simple Hopf bifurcation. Our formulation considers general repression functions which accommodate both $f_1$ and $f_2$.

Via numerical computations on the coupled-cell system and analyzing the phase response curve, it was asserted in Kim (2016), Kim et al. (2014) that the average frequency property holds at reasonable parameter values for the system with repression based on protein sequestration, whereas the collective frequency is far from the mean if modeled with Hill-type regulation. The mathematical models adopted therein are coupled-cell system comprising subsystems of the form (4) with $f=f_1, f_2$, respectively. It was also reported in Kim (2016), Kim and Forger (2012) that the properties of such coupled-cell system with protein-sequestration-based repression are consistent with data from *Drosophila* and mammals, whereas the properties of such coupled-cell system with Hill-type repression match well with the experimental data from *Neurospora*. It is therefore interesting to perform a further mathematical study to see and compare the oscillatory properties between the coupled-cell system modeled with $f=f_1$ and the one modeled with $f=f_2$.

Connection between the kinetic models and the phase models has been built in the so-called weakly coupled oscillators theory, cf. (Ermentrout and Kopell 1984, 1991; Schwemmer and Lewis 2012). However, such connection requires certain assumptions and a transparent correspondence between the dynamics of the kinetic models and its phase equation counterpart remains to be further explored. While weak coupling is a prerequisite in the theory, sufficiently large coupling strength is needed for the existence of phase-locked solution in some phase models. Thus, how weak is weak so that these connection and correspondence are valid appears to be a complicated issue. Recently, it has been reported that phase-amplitude reduction and higher-order reduction, instead of merely phase reduction and linear approximation, are necessary to capture the dynamics in some oscillatory systems, as reviewed in Ermentrout et al. (2019), Kuramoto and Nakao (2019).

The goal of our study is to explore the oscillatory properties of coupled-cell systems modeling biological rhythms. We shall consider the coupled-cell systems which are composed by the single-cell subsystem (4) with $f=f_1, f_2$, respectively. On the one hand, system (4) models the minimal genetic negative feedback loop in single cells, and it is essential to investigate oscillations in the coupled-cell system which comprises subsystems (4). On the other hand, the above-mentioned two issues about the properties of coupled oscillators and circadian clocks deserve a close analysis. The other important concern is about the comparison between the dynamics in the model using Hill-function repression and the one in the model based on protein sequestration repression. Although the mathematics in the kinetic models is in general rather involved, as commented in Baker and Schnell (2009), we take on the challenge to develop efficacious mathematical approaches to analyze the coupled-cell systems to see the dynamical details. In addition, the comparison between indication from the kinetic models and the one from the phase models can be made only after the kinetic models are sufficiently understood.

The first task is to confirm the existence of periodic solutions in the single-cell systems and the coupled-cell systems. Our methodology is based on the Hopf bifurcation theory (Hassard et al. 1981) and a sufficient-and-necessary condition for the simple Hopf bifurcation derived in Liu (1994), which was an extension of the Routh–Hurwitz criterion. As the parameter values vary, we seek for the situation that the determinant $D_{m-1}$ of the second-to-last Hurwitz matrix $H_{m-1}$ decreases from positive to zero, for a polynomial of degree *m*. A pair of complex-conjugate eigenvalues then cross the imaginary axis of the complex plane. This tracing allows to locate the Hopf bifurcation (HB) values.

The criterion in Liu (1994) for detecting Hopf bifurcation has been applied in several works on mathematical biology. For instance, for models on immune responses to persistent viruses, some ODE systems up to five dimension were considered in Liu (1997). In Domijan and Kirkilionis (2009), bistability and oscillations in chemical reaction networks were reported and the criterion was applied to a four-dimensional system of ODEs. In a study on extracellular signal-regulated kinase (Obatake et al. 2019), a polynomial of degree seven was tested to meet the criterion. In those applications, a common predicament is that the linearized system depends on the values of steady states and parameters. Therefore, the basic task is to seek for suitable parameter values, and hence, the steady-state value so that the associated characteristic polynomial meets the criterion. To this end, Newton polytope and symbolic computation using MAPLE programming were employed for such verification in Obatake et al. (2019) and Liu (1994), respectively. On the other hand, numerical bifurcation software, such as AUTO, is also able to locate the HB values and draw the HB curves in the parameter space.

Even with the approach by numerical computation, the condition $D_{m-1}=0$ is also helpful for detecting and confirming the HB values. In this work, we will see from this condition that the Hopf bifurcation does not occur at those parameter values and steady states where $f'({\bar{x}})$ is too small (${\bar{x}}$ is one of the component of the steady state). In addition, the HB value for the single-cell system is larger than the HB value for the coupled-cell system, in the identical-cell case. More information about the Hopf bifurcation and the frequency of bifurcating periodic solution can also be observed from this condition.

While most of the literature about the application of Hopf bifurcation, including the above-mentioned ones, is only concerned with existence of periodic solutions, the Hopf bifurcation theory actually implicates more. It provides a foundation for our concern about the properties for the frequencies of oscillations. At the HB value, the magnitude of the purely imaginary eigenvalue gives the frequency of the emergent periodic solution. As this purely imaginary eigenvalue is a root of the characteristic polynomial, for the identical-cell case, we can link the frequency of oscillation to the parameters and steady states, at the HB values. It is interesting to observe that for the single-cell systems, the repression function *f* does not play a role in the size of frequency at the HB value. In contrast, $\gamma :=-f'({\bar{x}})$ is a factor to the frequency at the HB value in the coupled-cell systems, and thus, the modeling with $f=f_1$ or $f=f_2$ is distinguishable in this respect.

The paper is organized as follows. In Sect. 2, we introduce the Hopf bifurcation theory and the degenerate Routh–Hurwitz criterion to study the existence and stability of periodic solutions. We apply these theories to obtain periodic solutions in the single-cell system (4) with $$f=f_1$$ and $$ f_2$$, respectively, in Sect. 3. In Sect. 4, we discuss the collective frequency of periodic solutions for the coupled-cell system and compare it with the average of individual frequencies. In addition, we make a comparison between the system with Hill-function repression and the one with protein-sequestration-based repression. To compare with other biological oscillators, in Sect. 5, we conduct similar analysis on a segmentation clock model and make a comparison between these different types of biological oscillators. Some justifications and computations are arranged in Appendices and Supplementary Materials.

## Hopf Bifurcation and Degenerate Routh–Hurwitz Criterion

In this section, we review the Hopf bifurcation theory and an extension of the Routh–Hurwitz criterion, which are to be applied to investigate the stable periodic solutions and their periods for the single-cell systems and the coupled-cell systems. Hopf bifurcation theory not only confirms the existence of periodic solution, but also indicates that at the Hopf bifurcation value, the purely imaginary eigenvalue of the linearized system provides the frequency of the bifurcating periodic solution. The Routh–Hurwitz criterion characterizes a polynomial whose roots all have negative real parts. We shall apply a degeneracy of this criterion, established in Liu (1994), that provides a condition for the Hopf bifurcation.

Let us consider an autonomous system of ODEs

7 $$\begin{aligned} \dot{{\mathbf{x}}} = {\mathbf{F}}({\mathbf{x}};{\mathbf{p}}), \end{aligned}$$

where ${\mathbf{x}}=(x_1, \ldots , x_m)\in {\mathbb {R}}^m$, ${\mathbf{F}}=(F_1, \ldots , F_m)$, and ${\mathbf{p}} \in {\mathbb {R}}^k$ are parameters. Let ${\bar{\mathbf{x}}}$ be an equilibrium of system (7) with a fixed ${\mathbf{p}}$. Denote the Jacobian matrix associated with the linearization of system (7) at ${\bar{\mathbf{x}}}$ by $J({\bar{\mathbf{x}}}):=\left( \frac{\partial F_i}{\partial x_j}({\bar{\mathbf{x}}},{\mathbf{p}}) \right) _{m \times m}$. Its characteristic polynomial is

8 $$\begin{aligned} \varDelta (\lambda )&:= {\mathrm{det}}(\lambda I - J({\bar{\mathbf{x}}})) \nonumber \\&= \lambda ^m + b_1\lambda ^{m-1}+b_2\lambda ^{m-2}+\cdots +b_{m-2}\lambda ^2+b_{m-1}\lambda +b_m. \end{aligned}$$

The Hurwitz matrices associated with polynomial $\varDelta (\lambda )$ are defined as

9 $$\begin{aligned}&H_1= \left[ b_1 \right] ,~ H_2 = \left[ \begin{array}{cc} b_1&{}b_3\\ 1&{} b_2 \end{array}\right] , ~ H_3 = \left[ \begin{array}{ccc} b_1&{}b_3 &{}b_5\\ 1&{} b_2 &{}b_4\\ 0 &{} b_1 &{} b_3 \end{array}\right] , ~ \cdots , \nonumber \\&H_m = \left[ \begin{array}{ccccccc} ~b_1~ &{} ~b_3~ &{} ~b_5~ &{} ~\cdots ~ &{} ~\cdots ~ &{} ~b_{2m-3}~ &{} ~b_{2m-1}~ \\ ~1~ &{} ~b_2~ &{} ~b_4~ &{} &{} &{} ~\vdots ~ &{} ~\vdots ~ \\ ~0~ &{} ~b_1~ &{} ~b_3~ &{} &{} &{} ~\vdots ~ &{} ~\vdots ~ \\ ~0 ~ &{} ~1~ &{} ~b_2~ &{} ~\ddots ~ &{} &{} ~\vdots ~ &{} ~\vdots ~ \\ ~\vdots ~ &{} ~\vdots ~ &{} ~\vdots ~ &{} &{} ~\ddots ~ &{} ~\vdots ~ &{} ~\vdots ~ \\ ~\vdots ~ &{} ~\vdots ~ &{} ~\vdots ~ &{} &{} &{} ~b_{m-1} ~ &{} ~0 ~ \\ ~0~ &{} ~0~ &{} ~0~ &{} \cdots &{} \cdots &{} ~b_{m-2}~ &{} ~b_m~ \\ \end{array}\right] . \end{aligned}$$

The Routh–Hurwitz criterion indicates that all roots of (8) have negative real parts if and only if $D_i := \mathrm{det}(H_i) >0$, $i=1,2,\ldots ,m$, cf. (Gantmacher 1959; Hurwitz 1895; Kemperman 1982). Note that $D_m = b_m \cdot D_{m-1}$, and so the Routh–Hurwitz criterion can also be expressed by $D_i>0$, $i=1,2,\ldots ,m-1$, with $b_m>0$.

The following lemma completely characterizes a polynomial which has a pair of purely imaginary roots and negative real parts for all the remaining roots. It can be regarded as a degeneracy of the Routh–Hurwitz criterion.

### Lemma 1

$\varDelta (\lambda )$ has a pair of purely imaginary roots, and all other roots have negative real parts if and only if

$$\begin{aligned} b_m> 0,~D_i>0,~i=1,2,\ldots ,m-2,~\mathrm{and}~D_{m-1}=0. \end{aligned}$$

We note that the Routh–Hurwitz criterion has also been formulated as positive determinants of a sequence of matrices different from (9), see (Uspensky 1948). With $D_i$ defined as the determinants of such sequence of matrices, this lemma was proved in Liu (1994). By similar arguments, it can be justified that Lemma 1 holds true if we adopt $D_i$, the determinant of Hurwitz matrices $H_i$ defined in (9).

To apply the Hopf bifurcation theorem, one first needs to find the parameter values at which a complex-conjugate pair of eigenvalues of $J({\bar{\mathbf{x}}})$ cross the imaginary axis of the complex plane transversally and the real parts of the other eigenvalues remain negative. In a system modeling a biological process, there are usually a number of parameters. We choose one of the parameters as the bifurcation parameter, denoted by $\alpha$ in the following discussions, and fix the other parameters at suitable values. By doing so, we regard the equilibrium $\bar{{\mathbf{x}}}$ as a function of $\alpha$, i.e., $\bar{{\mathbf{x}}}=\bar{{\mathbf{x}}}(\alpha )$. The Jacobian matrix associated with the linearization of (7) at $\bar{{\mathbf{x}}}(\alpha )$ then depends on $\alpha$, i.e., $J(\bar{\mathbf{x}}(\alpha );\alpha )$ and is abbreviated as $J(\bar{\mathbf{x}};\alpha )$. The following Hopf bifurcation theorem was stated in Liu (1994), where instead of computing the eigenvalues of $J(\bar{\mathbf{x}};\alpha )$, one only needs to compute the determinants of the Hurwitz matrices.

### Theorem 1

Consider system (7) whose equilibrium $\bar{\mathbf{x}} = \bar{\mathbf{x}}(\alpha )$ is a function of $\alpha$ for $\alpha$ near certain $\alpha ^*$, when the other parameters are fixed. Assume that at $\alpha = \alpha ^*$, the following conditions hold

$$\begin{aligned}&b_m(\alpha ^*)> 0,~D_i(\alpha ^*) > 0,~i=1,2,\ldots ,m-2,~D_{m-1}(\alpha ^*)=0, \\&\frac{\mathrm{d}}{\mathrm{d}\alpha }[D_{m-1}(\alpha )]\vert _{\alpha =\alpha ^*}\ne 0. \end{aligned}$$

Then, the system undergoes a Hopf bifurcation at ${{\mathbf{x}}}=\bar{{\mathbf{x}}}(\alpha ^*)$, and a small-amplitude periodic solution surrounding $\bar{{\mathbf{x}}}$ emerges as $\alpha <\alpha ^*$ or $\alpha >\alpha ^*$ and $\alpha$ is close to $\alpha ^*$.

The scenario in Theorem 1 was called simple Hopf bifurcation in Liu (1994), as there are more complicated Hopf bifurcations, see (Golubitsky and Schaeffer 1985). The value $\alpha ^*$ in Theorem 1 is called Hopf bifurcation (HB) value. The advantage of Theorem 1 is that its conditions are transparent and computable, and the existence of periodic solutions is guaranteed. When $m=3, 4$, the conditions of the theorem can be clearly expressed by

10 $$\begin{aligned} m=3:&b_1(\alpha ^*)> 0,~b_3(\alpha ^*)>0,~b_1(\alpha ^*)b_2(\alpha ^*)-b_3(\alpha ^*)=0, \end{aligned}$$

11 $$\begin{aligned}&~\frac{\mathrm{d}}{\mathrm{d}\alpha }[b_1(\alpha )b_2(\alpha )-b_3(\alpha )]\vert _{\alpha =\alpha ^*}\ne 0, \end{aligned}$$

12 $$\begin{aligned} m=4:&b_1(\alpha ^*)> 0,~b_3(\alpha ^*)>0,~b_4(\alpha ^*)>0, \nonumber \\&b_1(\alpha ^*)b_2(\alpha ^*)b_3(\alpha ^*)-b_3^2(\alpha ^*)-b_1^2(\alpha ^*)b_4(\alpha ^*)=0, \end{aligned}$$

13 $$\begin{aligned}&\frac{\mathrm{d}}{\mathrm{d}\alpha }[b_1(\alpha )b_2(\alpha )b_3(\alpha )-b_3^2(\alpha )-b_1^2(\alpha )b_4(\alpha )]\vert _{\alpha =\alpha ^*}\ne 0 . \end{aligned}$$

To apply the Hopf bifurcation theory to the ODE systems, we look for values of $\alpha ^*$ and values of the other parameters, which satisfy the conditions of Theorem 1. This process relies on some mathematical formulations and numerical computations.

Hopf bifurcation theory actually provides more information, including the stability and period of the bifurcating periodic solution. The period (frequency) is especially the focus of the present investigation. Stability of the bifurcating periodic solutions is determined by some higher-order terms of the system. One first transforms the system into normal form and then applies the center manifold theorem to obtain these terms. While this formulation is standard, its computation is cumbersome for large *m*. We summarize this process in Supplementary Material I, where the terms $$g_{20}, g_{11}, g_{02}$$, and $$ g_{21}$$, and hence $$C_{1}(\alpha ^*)$$, $$p_2$$, $$\zeta _{2}$$, and $$T_2$$ are introduced. Let $$\lambda (\alpha )$$ be the branch of eigenvalue crossing the imaginary axis at $$\alpha =\alpha ^*$$, and $$\lambda (\alpha ^*)=i \omega ^*$$, with $$\omega ^* >0$$. The following Hopf bifurcation theorem can be found in Hassard et al. (1981).

### Theorem 2

Under the conditions of Theorem 1, the periods of the bifurcating periodic solutions of system (7) are

$$\begin{aligned} T = \frac{2\pi }{\omega ^*}(1+T_2\varepsilon ^2+O(\varepsilon ^4)), \end{aligned}$$

where

$$\begin{aligned} \varepsilon ^2 = \frac{\alpha -\alpha ^*}{p_2}+O((\alpha -\alpha ^*)^2). \end{aligned}$$

Furthermore,

(i) the Hopf bifurcation is supercritical (resp., subcritical) and the periodic solution exists for $\alpha >\alpha ^*$ (resp., $\alpha < \alpha ^*$) with $\alpha$ near $\alpha ^*$, provided $p_2>0$ (resp., $p_2<0$);
(ii) the periodic solution is stable (resp., unstable), provided $\zeta _2<0$ (resp., $\zeta _2>0$);
(iii) the period *T* increases (resp., decreases) as $\alpha$ increases, provided $T_2>0$ (resp., $T_2<0$) and $p_2>0$, and decreases (resp., increases) as $\alpha$ decreases, provided $T_2<0$ (resp., $T_2>0$) and $p_2<0$.

We note that numerical bifurcation software such as AUTO can also detect the Hopf bifurcation and draw the HB curve which comprises the HB values in the parameter space. However, the condition in Theorem 1 allows us to analyze how Hopf bifurcation occurs and locate and confirm the HB values via numerical computations. In addition, from Theorem 2, the frequency of the bifurcating periodic solution is given by the magnitude of the purely imaginary eigenvalue which is the root of the characteristic polynomial. One can thus link the frequency to the parameters and the HB values.

## Single-Cell HT Model and PS Model

In this section, we consider system (4), abbreviated as HT model for $f=f_1$, the Hill-type repression, and as PS model for $f=f_2$, the protein-sequestration-based repression. We shall apply Theorem  1 in Sect. 2 to locate the parameter values at which the Hopf bifurcation takes place in system (4). Near these bifurcation values, Theorem 2 provides the approximate periods for the bifurcating stable periodic solutions.

By assuming that the degradation rates are equal

14 $$\begin{aligned} \beta :=\beta _1=\beta _2=\beta _3, \end{aligned}$$

and scaling the variables and time

$$\begin{aligned} y_1:=\beta M/\alpha _1, ~ y_2:=\beta ^2 P/(\alpha _1 \alpha _2), y_3:=\beta ^3 R/(\alpha _1 \alpha _2 \alpha _3), ~ \tau =\beta t, \end{aligned}$$

Equation (4) can be transformed into a non-dimensional system:

15 $$\begin{aligned} \left\{ \begin{aligned} \displaystyle {\dot{y}}_1&= f(y_3) - y_1 \\ \displaystyle {\dot{y}}_2&= y_1 - y_2 \\ \displaystyle {\dot{y}}_3&= y_2 - y_3, \end{aligned} \right. \end{aligned}$$

where ${\dot{y}}_1, {\dot{y}}_2, {\dot{y}}_3$ are derivatives with respect to $\tau$, and

$$\begin{aligned}&f(y_3)=f_1(y_3)= \frac{1}{1+(y_3/{\tilde{k}}_H)^{n}},\\&\quad {\mathrm{or}} f(y_3)=f_2(y_3)= \frac{{\tilde{A}}-y_3-{\tilde{k}}_d+\sqrt{({\tilde{A}}-y_3-{\tilde{k}}_d)^2+4{\tilde{A}} {\tilde{k}}_d}}{2{\tilde{A}}},\\&{\tilde{k}}_d:= \frac{\beta ^3}{\alpha _1 \alpha _2 \alpha _3} k_d, ~{\tilde{A}}:= \frac{\beta ^3}{\alpha _1 \alpha _2 \alpha _3}A, ~{\tilde{k}}_H:= \frac{\beta ^3}{\alpha _1 \alpha _2 \alpha _3} k_\mathrm{H}. \end{aligned}$$

Herein, we retain the same notations $f_1, f_2$, since they have the same forms as (5) and (6). System of equations in form (15) has been studied in Kim (2016), Kim et al. (2014) through numerical simulations.

To analyze oscillatory properties for these models via Hopf bifurcation, herein, we consider another change of variables. Under the same assumption (14), we set

$$\begin{aligned} x_1=(\alpha _2\alpha _3/\beta ^2) M, x_2=(\alpha _3/\beta )P, ~x_3=R,~ \tau =\beta t. \end{aligned}$$

Then, system (4) can be transformed into

16 $$\begin{aligned} \left\{ \begin{aligned} \displaystyle {\dot{x}}_1&= \alpha f(x_3) - x_1 \\ \displaystyle {\dot{x}}_2&= x_1 - x_2 \\ \displaystyle {\dot{x}}_3&= x_2 - x_3, \end{aligned} \right. \end{aligned}$$

where $\alpha := \alpha _1\alpha _2\alpha _3/\beta ^3$ is to serve as the bifurcation parameter.

We call system (16) with $f=f_1$ the *single-cell HT model*. In this system, the ratio of rates $\alpha$, the Hill coefficient *n*, and dissociation constant $k_\mathrm{H}$ between the repressor and gene promoter determine the dynamics. We call system (16) with $f=f_2$ the *single-cell PS model*, where the rate ratio $\alpha$, activator concentration *A*, and dissociation constant $k_d$ between the activator and repressor, determine the dynamics. On the one hand, system (16) with $\alpha =1$ reduces to system (15). On the other hand, dynamical properties of system (16) certainly correspond to the kinetics in system (4). System in the form (16) with $f=f_1$ has also been studied in Woller et al. (2014). We now take $\alpha$ as the bifurcation parameter and apply Theorems  1 and 2 to investigate the periodic solutions of system (16).

Note that $\bar{{\mathbf{x}}}= ({\bar{x}}_1,{\bar{x}}_2,{\bar{x}}_3)$ is a positive equilibrium of system (16) if and only if ${\bar{x}}_1={\bar{x}}_2={\bar{x}}_3={\bar{x}}>0$, and

17 $$\begin{aligned} \alpha f({\bar{x}})={\bar{x}}. \end{aligned}$$

Such ${\bar{x}}$ uniquely exists for $f=f_1$ or $f=f_2$:

### Proposition 1

There exists a unique positive equilibrium $\bar{{\mathbf{x}}}= ({\bar{x}},{\bar{x}},{\bar{x}})$ for system (16) with $f=f_1$, for any $n\ge 1$, $k_\mathrm{H}, \alpha >0$, and for system (16) with $f=f_2$, for any *A*, $k_d$, $\alpha >0$, where ${\bar{x}}$ is the unique positive solution to $\alpha f_1({\bar{x}})={\bar{x}}$, and $\alpha f_2({\bar{x}})={\bar{x}}$, respectively. Moreover, for any fixed $n\ge 1$, $k_\mathrm{H} >0$, or fixed *A*, $k_d >0$, ${\bar{x}}$ is an increasing function of $\alpha$.

### Proof

It is obvious that $({\bar{x}}_1,{\bar{x}}_2,{\bar{x}}_3)$ is an equilibrium for system (16) if and only if ${\bar{x}}_1={\bar{x}}_2={\bar{x}}_3={\bar{x}}$ and ${\bar{x}}$ satisfies $\alpha f({\bar{x}})={\bar{x}}$. For $f=f_1$, this reads as

18 $$\begin{aligned} \frac{\alpha }{1+({\bar{x}}/k_\mathrm{H})^{n}} = {\bar{x}}, \end{aligned}$$

i.e., ${\bar{x}}$ satisfies $q(\xi )=\alpha k_\mathrm{H}^{n}$, where $q(\xi ):=\xi ^{n+1}+k_\mathrm{H}^{n}\xi$. For any $n\ge 1$, $k_\mathrm{H}, \alpha >0$, there exists exactly one positive solution to this equation, due to that *q* is strictly increasing on $[0, \infty )$, $q(\xi )\rightarrow \infty$ as $\xi \rightarrow \infty$, and $q(0)=0<\alpha k_\mathrm{H}^{n}$. We also see that ${\bar{x}}$ is a function of $\alpha$, as *q* is strictly increasing.

For $f=f_2$, we see that $f_2(\xi )>0$ for all $\xi \ge 0$, $f_2(0)>0$, $\lim _{\xi \rightarrow \infty } f_2(\xi )=0$, and $f_2'(\xi )<0$ for all $\xi > 0$. Thus, there exists exactly one positive solution to $\alpha f_2({\bar{x}})={\bar{x}}$. As $\xi /f_2(\xi )$ is strictly increasing and has an inverse, ${\bar{x}}$ is thus an increasing function of $\alpha$. $\square$

Denote $\bar{{\mathbf{x}}}=\bar{{\mathbf{x}}}(\alpha )$ to indicate the dependence on $\alpha$. We note that from (17), $\alpha$ can be expressed as a function of ${\bar{x}}$, i.e., $\alpha ={\bar{x}}/f({\bar{x}})$. This is the inverse expression of $\bar{{\mathbf{x}}}(\alpha )$. We shall analyze the periodic solutions bifurcating from $\bar{{\mathbf{x}}}$. The Jacobian matrix associated with the linearization of system (16) at $\bar{{\mathbf{x}}}$ is

$$\begin{aligned} J(\bar{{\mathbf{x}}};\alpha )=\left[ \begin{array}{ccc} ~-1 &{} 0 &{} -\alpha \gamma ~ \\ ~1 &{} -1 &{} 0~ \\ ~0 &{} 1 &{} -1~ \\ \end{array}\right] , \end{aligned}$$

where $\gamma :=-f'({\bar{x}})$. The characteristic polynomial $\varDelta (\lambda ):=\mathrm{det}(\lambda I-J(\bar{{\mathbf{x}}};\alpha ))$ is

19 $$\begin{aligned} \varDelta (\lambda )=\lambda ^3 + 3\lambda ^2 + 3\lambda + (\alpha \gamma +1). \end{aligned}$$

One can factorize this cubic polynomial and find all its roots. Certainly, we can also apply Theorem  1. From (10), we see that $\varDelta (\lambda )$ has a pair of purely imaginary roots and a negative root if and only if

20 $$\begin{aligned} \alpha =\alpha _0:=8/\gamma . \end{aligned}$$

Notice that $\gamma$ depends on ${\bar{x}}$ which is a function of $\alpha$. Hence, whether the Hopf bifurcation takes place is determined by the existence of solution ${\bar{x}}$ to

21 $$\begin{aligned} \alpha =-\frac{8}{f'({\bar{x}})}=\frac{{\bar{x}}}{f({\bar{x}})}. \end{aligned}$$

If such ${\bar{x}}$ exists, then the Hopf bifurcation occurs at equilibrium $({\bar{x}},{\bar{x}},{\bar{x}})$ and $\alpha =\alpha _0=-8/f'({\bar{x}})$. Let us elaborate to confirm such existence for $f=f_1$ and $f=f_2$, respectively. Since $\gamma$ will play an important role in the analysis, we denote $\gamma _1:=-f_1'({\bar{x}})$ and $\gamma _2:=-f_2'({\bar{x}})$.

For $$f=f_1$$,

22 $$\begin{aligned} \gamma _1 =-f'_1({\bar{x}})= \frac{k_\mathrm{H}^{n} n {\bar{x}}^{n-1}}{(k_\mathrm{H}^{n}+{\bar{x}}^n)^2}. \end{aligned}$$

At $\alpha =\alpha _0=8/\gamma _1$, solving (21) with $f=f_1$, we obtain

23 $$\begin{aligned} {\bar{x}} = k_\mathrm{H}\left( \frac{8}{n-8}\right) ^{\frac{1}{n}},~n>8. \end{aligned}$$

That is, for given $k_\mathrm{H}>0, n>8$, we set ${\bar{x}}$ as (23). Then, with $\alpha =\alpha _0=8/\gamma _1$ and $\gamma _1$ computed from (22), equality (18) holds, and hence $\bar{{\mathbf{x}}}= ({\bar{x}},{\bar{x}},{\bar{x}})$ is an equilibrium of system (16) with $f=f_1$.

For $$f=f_2$$,

24 $$\begin{aligned} \gamma _2 =-f_2'({\bar{x}})= \frac{A-{\bar{x}}-k_d+\sqrt{(A-{\bar{x}}-k_d)^2+4Ak_d}}{2A\sqrt{(A-{\bar{x}}-k_d)^2+4Ak_d}}. \end{aligned}$$

At $\alpha =\alpha _0=8/\gamma _2$, we solve (21) with $f=f_2$, and obtain

$$\begin{aligned} {\bar{x}} = \frac{64}{63}(A-k_d)\pm \frac{8}{63}\sqrt{(A-k_d)^2-252 A k_d}, \end{aligned}$$

where the square root is nonnegative provided

$$\begin{aligned} A \ge (127+48\sqrt{7})k_d ~\mathrm{or}~ A \le (127-48\sqrt{7})k_d. \end{aligned}$$

Herein, we consider

25 $$\begin{aligned} A > (127+48\sqrt{7})k_d \end{aligned}$$

and choose

26 $$\begin{aligned} {\bar{x}} = \frac{64}{63}(A-k_d)-\frac{8}{63}\sqrt{(A-k_d)^2-252 A k_d} \end{aligned}$$

so that ${\bar{x}}$ is positive and the corresponding $\alpha _0=8/\gamma _2=-8/f'_2({\bar{x}})$ is not large.

Denote $\gamma (\alpha )=\gamma ({\bar{x}}(\alpha ))$. For crossing condition (11), we have

27 $$\begin{aligned} \frac{\mathrm{d}}{\mathrm{d}\alpha }[b_1(\alpha )b_2(\alpha )-b_3(\alpha )]\vert _{\alpha = \alpha _0}= & {} \frac{\mathrm{d}}{\mathrm{d}\alpha }[8-\alpha \gamma ]\vert _{\alpha =\alpha _0} \nonumber \\= & {} \left[ -\gamma -\alpha \cdot \frac{\mathrm{d}\gamma }{\mathrm{d} \alpha }\right] \vert _{\alpha =\alpha _0}, \end{aligned}$$

where $\gamma =\gamma _1$ if $f=f_1$, and $\gamma =\gamma _2$ if $f=f_2$. Note that $\gamma =-f'({\bar{x}})$ is always positive for any smooth and strictly decreasing function *f*, including $f_1$ and $f_2$. We confirm $\gamma _1+\alpha \cdot \mathrm{d}\gamma _1/\mathrm{d} \alpha \ne 0$ provided $n>8$, and $\gamma _2+\alpha \cdot \mathrm{d}\gamma _2/\mathrm{d} \alpha \ne 0$ for all $A \ne A^*$, under condition (25), at $\alpha =\alpha _0$, in Appendix A, where $A^*$ is defined. That is, $n>8$ is both a sufficient and a necessary condition for the simple Hopf bifurcation of system (16) with $f=f_1$.

According to Theorem 1, system (16) undergoes a Hopf bifurcation at ${\mathbf{x}}=\bar{\mathbf{x}}(\alpha _0)$ and $\alpha = \alpha _0$, and a small-amplitude periodic solution near $\bar{\mathbf{x}}$ emerges as $\alpha <\alpha _0$ or $\alpha >\alpha _0$ and $\alpha$ is close to $\alpha _0$. Notice that at $\alpha =\alpha _0=8/\gamma$,

28 $$\begin{aligned} J(\bar{{\mathbf{x}}};\alpha _0)=\left[ \begin{array}{ccc} ~-1 &{} 0 &{} -8~ \\ ~1 &{} -1 &{} 0~ \\ ~0 &{} 1 &{} -1~ \end{array}\right] , \end{aligned}$$

a constant matrix. The eigenvalues of this matrix are $\lambda _{1,2}=\pm \sqrt{3} i, \lambda _{3}=-3$. Therefore, the bifurcating periodic solution has frequency near $\omega _0 =\sqrt{3}$, for $\alpha$ close to $\alpha _0$, for either $f=f_1$ or $f=f_2$, by Theorem 2. We emphasize that for any repression function *f* in system (16), when the simple Hopf bifurcation takes place, the bifurcating periodic solution always has frequency about $\sqrt{3}$. Let us summarize:

### Theorem 3

Assume that $n>8$ if $f=f_1$, and (25) holds and $A \ne A^*$ if $f=f_2$. System (16) undergoes a Hopf bifurcation at $\alpha =\alpha _0=8/\gamma$, where $\gamma =-f'({\bar{x}})$, and a small-amplitude periodic solution near $\bar{\mathbf{x}}=\bar{\mathbf{x}}(\alpha _0)$ emerges as $\alpha <\alpha _0$ or $\alpha >\alpha _0$ and $\alpha$ is close to $\alpha _0$, with frequency about $\omega _0 = \sqrt{3}$.

### Remark 1

(i) If we multiply each component in system (16) by a factor $\sigma$, or change the time from *t* to $t/\sigma$, then the eigenvalues of the linearized system at $\bar{\mathbf{x}}$ become $\lambda _{1,2}=\pm i \sqrt{3} \sigma , \lambda _{3}=-3\sigma$. The system still undergoes a Hopf bifurcation at $\alpha = \alpha _0$ and ${\mathbf{x}}=\bar{\mathbf{x}}$, and the frequency of the bifurcating periodic solution is near $\sigma \sqrt{3}$. This property will be used in Sect. 4.2 for a cell-to-cell system which has two different individual frequencies.
(ii) For the general situation of degradation rates, i.e., when assumption (14) does not hold, assertions similar to the ones in Theorem 3 still hold. In particular, the frequency $\omega _0$ at the HB value is a combination of the degradation rates (no longer $\sqrt{3}$), but still does not depend on $\gamma$, and hence does not differentiate the form of repression function *f*. In addition, for $f=f_1$, *n* will be required to be much larger than 8, if the difference among $\beta _1, \beta _2, \beta _3$ is large. This was mentioned in (Fall et al. 2002) and is shown precisely in Shih and Yang (2021).

The following examples illustrate Theorems 2 and 3. The parameter values considered here are mostly taken from Kim et al. (2014).

### Example 3.1

**Fig. 2:**
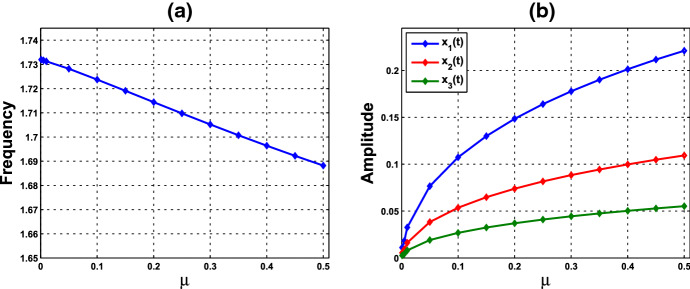
**a** Frequencies and **b** amplitudes of oscillations corresponding to various values of $\mu$, where $\mu :=\alpha - \alpha _0$, $\alpha _0\approx 0.545174$, for the single-cell HT model in Example 3.1

Consider system (16) with $f=f_1$ and parameter values $n=11$ and $k_\mathrm{H}=0.136$. The graph of $f_1$ is depicted in Fig. 1a. According to Theorem 3, as $n>8$, the Hopf bifurcation occurs at $\bar{{\mathbf{x}}}=({\bar{x}},{\bar{x}},{\bar{x}})$, where ${\bar{x}}\approx 0.148684$, computed from (23). Next, we compute to find $\gamma _1\approx 14.674227$ from (22). Accordingly, a small-amplitude periodic solution emerges as the value of $\alpha$ increases through $\alpha _0=8/\gamma _1 \approx 0.545174$, with frequency about $\sqrt{3}\approx 1.732051$. Furthermore, we compute to find that $p_2>0, \zeta _2<0, T_2>0$. The numerics are shown in Supplementary Material I. According to Theorem 2, system (16) with $f=f_1$ undergoes a supercritical Hopf bifurcation at $\alpha = \alpha _0$ and $\bar{{\mathbf{x}}}=({\bar{x}},{\bar{x}},{\bar{x}})$. The bifurcating periodic solution is stable and the period increases as $\alpha$ increases. We numerically solve system (16) and compute the frequencies and amplitudes of the periodic solutions corresponding to various values of $\mu :=\alpha - \alpha _0$, plotted in Fig. 2. It can be seen that as $\mu$ increases from 0, the frequency decreases from about $\sqrt{3}$ (i.e., the period is increasing), which is consistent with the assertion of Theorem 2.

Next, we adopt the parameter values in Kim et al. (2014) and illustrate that the numerically computed oscillations therein appear to be a continuation of the periodic solutions generated by the Hopf bifurcation.

### Example 3.2

**Fig. 3:**
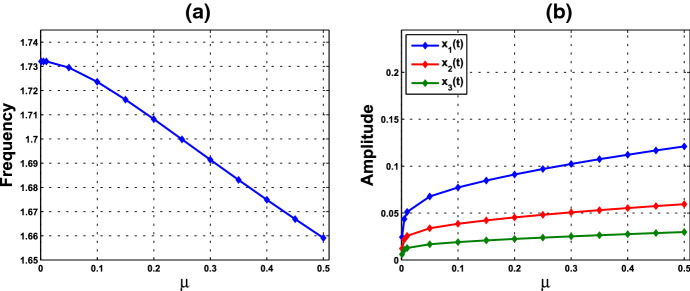
**a** Frequencies and **b** amplitudes of oscillations corresponding to various values of $\mu$, where $\mu :=\alpha - \alpha _0$, $\alpha _0\approx 0.533809$, for the single-cell PS model in Example 3.2

Consider system (16) with $f=f_2$, with parameter values $A=0.0659$ and $k_d=0.00001$. The graph of $f_2$ is depicted in Fig. 1b. As condition (25) in Theorem 3 is met, the Hopf bifurcation occurs at $\bar{{\mathbf{x}}}=({\bar{x}},{\bar{x}},{\bar{x}})$, with ${\bar{x}}\approx 0.058730$ computed from (26). We compute to find $\gamma _1\approx 14.986634$ from (24), and thus $\alpha _0=8/\gamma _1\approx 0.533809$. Therefore, a small-amplitude periodic solution emerges, as the value of $\alpha$ passes through $\alpha _0$, with frequency about $\sqrt{3}$. Furthermore, we compute to find that $p_2>0, \zeta _2<0, T_2>0$. The numerics are revealed in Supplementary Material I. According to Theorem 2, system (16) with $f=f_2$ undergoes a supercritical Hopf bifurcation at $\alpha = \alpha _0$ and $\bar{{\mathbf{x}}}=({\bar{x}},{\bar{x}},{\bar{x}})$. The bifurcating periodic solution is stable and the period increases as $\alpha$ increases. The frequencies and amplitudes corresponding to various values of $\mu :=\alpha -\alpha _0$ are plotted in Fig. 3. When $\alpha = 1$ ($\mu =0.466191$), system (16) becomes (15), and the parameter values herein are exactly the ones adopted in Kim et al. (2014). The system with such parameter values generates an oscillation with frequency about 1.664346.

**A comparison and summary** Let us summarize the above discussions, and make a comparison between the single-cell HT model and PS model. For either $$f=f_1$$ or $$f=f_2$$, system (16) has a unique equilibrium $$\bar{{\mathbf{x}}}=({\bar{x}},{\bar{x}},{\bar{x}})$$. For each set of parameters with $$n>8$$ for the case $$f=f_1$$, or satisfying (25) for the case $$f=f_2$$, the Hopf bifurcation occurs at $$(\alpha _0, \bar{{\mathbf{x}}})$$. The Jacobian matrix associated with the linearization of system (16) at $$\bar{{\mathbf{x}}}$$ with $$\alpha =\alpha _0$$ is a constant matrix. Restated, it is always this matrix whenever the Hopf bifurcation takes place in system (16), with $$f=f_1$$ or $$ f_2$$, or other smooth decreasing functions. Subsequently, the frequency of the bifurcating periodic solution near the bifurcation point is about $$\sqrt{3}$$. As $$\alpha $$ increases, the frequency decreases and the amplitude increases in both models, but the slopes are slightly different between these two models. The frequency drops faster in the single-cell PS model, whereas the amplitude increases faster in the single-cell HT model, see Figs. 2 and 3.

**Fig. 4:**
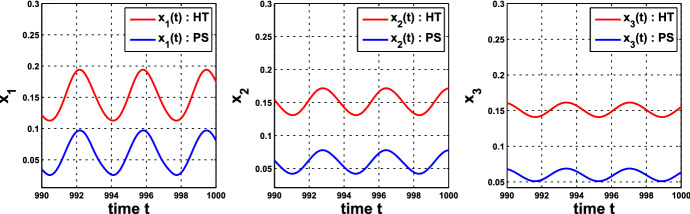
Components $x_1$, $x_2$, and $x_3$ of the solutions of system (16) with $f_1$ in Example 3.1 (red), evolved from (0.1, 0.1, 0.1), and $f_2$ in Examples 3.2 (blue), evolved from (0.295, 0.295, 0.295), with $\alpha = 0.60139$. The periods are 3.6367989020 and 3.6367987711 for the HT model and the PS model, respectively (Color figure online)

The parameters in Hill-function repression are different from the ones in protein-sequestration-based repression. So how would one choose parameter values in each of these two models to make comparison? In Examples 3.1, 3.2, we have chosen the parameter values $n=11$, $k_\mathrm{H}=0.136$ in the single-cell HT model and $A=0.0659$, $k_d=0.00001$ in the single-cell PS model. At $\alpha = 0.60139$, the oscillatory wave form in system (16) with $f=f_1$ is pretty close to the one with $f=f_2$, as demonstrated in Fig. 4. This is a consequence of Theorem 3.

### Remark 2

Recall that system (15) is identical to system (16) with $\alpha =1$. In Kim et al. (2014), system (15) was considered with $n=11$, $k_\mathrm{H}=0.04$ in the HT model and $A=0.0659$, $k_d=0.00001$ in the PS model. While the amplitudes in the two models with these parameter values are close to each other, our computation shows that the period for the single-cell HT model is 3.9302912, whereas the period for the single-cell PS model is 3.7751695. Therefore, we take $k_\mathrm{H}=0.136$ in the HT model in Example 3.1, and we can tune $\alpha$ so that the oscillatory wave forms in these two models are similar at $\alpha = 0.60139$.

## Coupled-Cell HT Model and PS Model

The synchronous or coherent rhythmicity for a collection of clock neurons is mediated by the neurotransmitters. It has been identified that vasoactive intestinal polypeptide (VIP) is the key synchronizing agent in several experiments (Aton et al. 2005; To et al. 2007). The collective period of oscillation is generated through intercellular coupling which is determined by the concentrations of neurotransmitters in extracellular medium. Modeling with such intercellular signaling via VIP, the following coupled-cell system has been considered in Kim et al. (2014) to investigate the average frequency property:

29 $$\begin{aligned} \left\{ \begin{aligned} {\dot{M}}_i&= \displaystyle f(R_i) - M_i + \frac{c}{N}\sum _{j=1}^{N} V_j \\ \displaystyle {\dot{P}}_i&= M_i - P_i \\ \displaystyle {\dot{R}}_i&= P_i - R_i \\ \displaystyle {\dot{V}}_i&= s[f(R_i)-V_i], \end{aligned} \right. \end{aligned}$$

where $i=1,2,\ldots ,N$, and parameters *c* and *s* are the coupling strength and the timescale of intercellular coupling, respectively. In this model, each cell releases VIP into the extracellular space at a rate proportional to the activity of the promoter *f*(*R*). The fourth component of the system indicates the release of *V* (VIP) by each cell into the extracellular space at a rate proportional to *f*(*R*) which describes the activity of the promoter. Note that the experimental data indicate that the intercellular coupling strength occurs much faster, compared to the intracellular feedback loop (An et al. 2011). This model is also based on the fact that the release of VIP is fast with respect to the 24 hours timescale, and thus the mean field expression $(\sum _{j=1}^{N} V_j)/N$ is adopted.

When $c=0$, the cells are decoupled, and we consider the parameter values at which each cell oscillates at the same frequency, as these *N* subsystems are identical. As *c* increases from 0, the cells become coupled. However, whether a collective periodic solution of the coupled system is then generated requires a justification.

In this section, we consider the coupled-cell HT model ($f=f_1$) and the coupled-cell PS model ($f=f_2$). We apply Theorem 1 to confirm the existence of periodic solution for these coupled-cell systems. We shall investigate how coupling strength affects the collective oscillations, and whether and how average frequency property holds in these two coupled systems. Referring to the frequency of periodic solution indicated in Theorem 2, we trace the eigenvalues of the linearized systems from the HB value for the single-cell subsystems ($c=0$) to the HB value for the coupled-cell system, and see which eigenvalue branch reaches the imaginary axis of the complex plane. Also, we compare the variations of collective frequency in these two models as the coupling strength *c* increases. In Sect. 4.1, we discuss the coupled system comprising two identical cells. This analysis is also valid for the coupled system comprising *N* identical cells, i.e., system (29). The coupled system comprising two nonidentical cells will be addressed in Sect. 4.2.

We note that synchronization in a more complicated model with Michaelian kinetics for the degradations and coupling through the mean field, has been reported in Gonze et al. (2005).

### Identical Cells

In this section, we consider coupled system (29) which consists of two identical cells, expressed by

30 $$\begin{aligned} \left\{ \begin{aligned} {\dot{x}}_1&= \displaystyle \alpha f(x_3) - x_1 + \frac{c}{2}[x_4 + y_4] \\ \displaystyle {\dot{x}}_2&= x_1 - x_2 \\ \displaystyle {\dot{x}}_3&= x_2 - x_3 \\ \displaystyle {\dot{x}}_4&= s[f(x_3)-x_4] \\ {\dot{y}}_1&= \displaystyle \alpha f(y_3) - y_1 + \frac{c}{2}[y_4 + x_4] \\ \displaystyle {\dot{y}}_2&= y_1 - y_2 \\ \displaystyle {\dot{y}}_3&= y_2 - y_3 \\ \displaystyle {\dot{y}}_4&= s[f(y_3)-y_4], \end{aligned} \right. \end{aligned}$$

where parameter $c>0$ represents the coupling strength and $s>0$ is the timescale of intercellular coupling. The discussion herein is also valid for system (29) which comprises *N* identical cells, as remarked below. Denote ${\mathbf{x}}=(x_1, x_2, x_3, x_4)$, ${\mathbf{y}}=(y_1, y_2, y_3, y_4)$, and ${\mathbb {X}}=({\mathbf{x}}, {\mathbf{y}})$. The following proposition about the equilibrium is straightforward.

#### Proposition 2

For each set of parameter values, system (30) has a unique equilibrium $$\overline{{\mathbb {X}}}$$ which is homogeneous, i.e., $$\overline{{\mathbb {X}}}=(\bar{{\mathbf{x}}}, \bar{{\mathbf{x}}})$$, $$\bar{{\mathbf{x}}}=({\bar{x}}_1,{\bar{x}}_2,{\bar{x}}_3,{\bar{x}}_4)$$. For $$ f=f_1$$,

$$\begin{aligned} {\bar{x}}_1 = {\bar{x}}_2 = {\bar{x}}_3 ={\bar{x}},~{\bar{x}}_4 = 1/\left[ 1+\left( \frac{{\bar{x}}}{k_\mathrm{H}}\right) ^n\right] , \end{aligned}$$

and ${\bar{x}}$ is the unique positive solution to $(\alpha +c)f_1({\bar{x}})={\bar{x}}$; for $f=f_2$,

$$\begin{aligned} {\bar{x}}_1 = {\bar{x}}_2 = {\bar{x}}_3 ={\bar{x}},~{\bar{x}}_4 = [A-{\bar{x}}-k_d+\sqrt{(A-{\bar{x}}-k_d)^2+4Ak_d}]/2A, \end{aligned}$$

and ${\bar{x}}$ is the unique positive solution to $(\alpha +c)f_2({\bar{x}})={\bar{x}}$. Moreover, for any fixed $n \ge 1, k_\mathrm{H} >0$, or fixed $A, k_d >0$, and $c>0$, ${\bar{x}}$ is a function of $\alpha$.

We show that system (30) has only homogeneous equilibrium $\overline{{\mathbb {X}}}=(\bar{{\mathbf{x}}},\bar{{\mathbf{x}}})$ in Appendix B(I). Now we take $\alpha$ as the bifurcation parameter, while holding all other parameters fixed at suitable values, and apply Theorem 1 to confirm the existence of periodic solution bifurcating from $\overline{{\mathbb {X}}}$. The Jacobian matrix associated with the linearization of system (30) at $\overline{{\mathbb {X}}}$ is

$$\begin{aligned} J(\overline{{\mathbb {X}}};\alpha ):= \left[ \begin{array}{cccccccc} ~-1 &{} 0 &{} -\alpha \gamma &{} \displaystyle \frac{c}{2} &{} 0 &{} 0 &{} 0 &{} \displaystyle \frac{c}{2}~ \\ ~1 &{} -1 &{} 0 &{} 0 &{} 0 &{} 0 &{} 0 &{} 0~ \\ ~0 &{} 1 &{} -1 &{} 0 &{} 0 &{} 0 &{} 0 &{} 0~ \\ ~0 &{} 0 &{} -s\gamma &{} -s &{} 0 &{} 0 &{} 0 &{} 0~ \\ ~0 &{} 0 &{} 0 &{} \displaystyle \frac{c}{2} &{} -1 &{} 0 &{} -\alpha \gamma &{} \displaystyle \frac{c}{2}~ \\ ~0 &{} 0 &{} 0 &{} 0 &{} 1 &{} -1 &{} 0 &{} 0~ \\ ~0 &{} 0 &{} 0 &{} 0 &{} 0 &{} 1 &{} -1 &{} 0~ \\ ~0 &{} 0 &{} 0 &{} 0 &{} 0 &{} 0 &{} -s\gamma &{} -s~ \end{array}\right] \end{aligned}$$

where $\gamma :=-f'({\bar{x}})>0$. The characteristic polynomial $\varDelta (\lambda ) := \det (\lambda I- J(\overline{{\mathbb {X}}};\alpha ) )$ can be factored as

31 $$\begin{aligned} \varDelta (\lambda ) = \varDelta _{+}(\lambda )\cdot \varDelta _{-}(\lambda ), \end{aligned}$$

where

$$\begin{aligned} \varDelta _{\pm }(\lambda )&= \lambda ^4 +b_1\lambda ^3 +b_2\lambda ^2 + b_3\lambda + b_4^{\pm } \\ b_1&:= s+3,~b_2 := 3(s+1),~b_3 := \alpha \gamma +3s+1\\ b_4^{-}&:=s(\alpha \gamma +1),~b_4^{+}:=b_4^{-}+cs\gamma . \end{aligned}$$

This factorization can be made since system (30) consists of two identical subsystems and the synchronous set $\{x_1=y_1, x_2=y_2, x_3=y_3, x_4=y_4\}$ is invariant, see Remark 5. Note that $b_1>0$, $b_3>0$, and $b_4^+>b_4^->0$. Considering (12) for $\varDelta _{+}(\lambda )$ and $\varDelta _{-}(\lambda )$, respectively, we see that $D_3^+(\alpha )< D_3^-(\alpha )$, where

$$\begin{aligned} D_3^{\pm }(\alpha ):= b_1(\alpha )b_2(\alpha )b_3(\alpha )-b_3^2(\alpha ) -b_1^2(\alpha )b_4^{\pm }(\alpha ). \end{aligned}$$

That is, if $D_3^-(\alpha ^*)=0$ for some $\alpha ^*>0$, then $D_3^+(\alpha ^*)<0$, and thus the simple Hopf bifurcation does not occur from $D_3^-(\alpha ^*)=0$. Accordingly, $\varDelta (\lambda )$ has a pair of purely imaginary roots and six roots with negative real parts if and only if $D_3^+(\alpha ^*)=0$, by Lemma 1. From this equality, we find

32 $$\begin{aligned} \alpha ^*=\frac{1}{2\gamma }\left[ -(s-1)(s^2+4s+7) \pm (s+3)\sqrt{(s^2+3)^2-4sc\gamma }\right] , \end{aligned}$$

where the square root is positive if and only if

33 $$\begin{aligned} \gamma <\frac{(s^2+3)^2}{4sc}. \end{aligned}$$

Herein, we only consider the case $s>1$, and hence only the “+” one in (32). Then, $\alpha ^*>0$ provided

34 $$\begin{aligned} \gamma <\frac{8(s+1)^3}{cs(s+3)^2}. \end{aligned}$$

Note that $s>1$ implies

$$\begin{aligned} \frac{(s^2+3)^2}{4sc}>\frac{8(s+1)^3}{cs(s+3)^2}. \end{aligned}$$

Thus, (34) implies (33), provided $s>1$. The case $0<s\le 1$ can also be discussed.

For crossing condition in (13), we consider

35 $$\begin{aligned}&\frac{\mathrm{d}}{\mathrm{d}\alpha }[b_1(\alpha )b_2(\alpha )b_3(\alpha )-b_3^2(\alpha )-b_1^2(\alpha )b_4^{+}(\alpha )] \\&\quad =-[\alpha \gamma '(\alpha )+\gamma (\alpha )][(s-1)(s^2+4s+7)+2\alpha \gamma (\alpha )]-cs\gamma '(\alpha )(s+3)^2 \nonumber \end{aligned}$$

at $\alpha =\alpha ^*$. When $c=0$, (35) becomes

$$\begin{aligned} -\frac{(s+3)(s^2+3)[8\gamma '(\alpha _0)+\gamma ^2(\alpha _0)]}{\gamma (\alpha _0)}, \end{aligned}$$

where $\alpha _0 = 8/\gamma (\alpha _0)$, which is $(s+3)(s^2+3)$ multiplied to the term for determining the crossing condition in the decoupled single-cell case (27), discussed in Sect. 3, see Appendix A. Hence, by continuity, (35) is nonzero for small coupling strength *c*, under the condition in Theorem 3.

We confirm the existence of homogeneous equilibrium $\overline{{\mathbb {X}}}=(\bar{\mathbf{x}}, \bar{\mathbf{x}})$ at the bifurcation value $\alpha = \alpha ^*$ for system (30) with sufficiently small coupling strength *c* in Appendix B(II). By substituting $\alpha ^*$ in (32) into the fourth-degree polynomial $\varDelta _{+}(\lambda )$, we can actually find its purely imaginary roots $\pm i \omega _c^*$.

#### Theorem 4

Consider $s>1$ and assume that the equilibrium $\overline{{\mathbb {X}}}=(\bar{\mathbf{x}}, \bar{\mathbf{x}})$ exists at $\alpha = \alpha ^*$ defined in (32), and that (34) and the crossing condition hold. System (30) undergoes a Hopf bifurcation at $\alpha ^*$ and a small-amplitude periodic solution near $\overline{{\mathbb {X}}}$ emerges as $\alpha <\alpha ^*$ or $\alpha >\alpha ^*$ and $\alpha$ is close to $\alpha ^*$, with frequency $\omega _c$ about

$$\begin{aligned} \omega _c^* := \sqrt{\frac{1}{2}\left[ \sqrt{(s^2+3)^2-4sc\gamma }-(s^2-3)\right] }, \end{aligned}$$

where $\gamma =\gamma _1:= -f_1'({\bar{x}})$ if $f=f_1$, and $\gamma =\gamma _2:= -f_2'({\bar{x}})$ if $f=f_2$.

Notably, it can be shown that $\alpha ^*>0$ if and only if $\omega _c^* >0$. Certainly, the collective period of the bifurcating periodic solution is near $T_c^* = 2\pi /\omega _c^*$. We will provide further observation from the assertion of this theorem below.

**Implementation and Computation** First, we fix the parameter values of *n* and $$k_\mathrm{H}$$ in the HT model, and *A* and $$ k_d$$ in the PS model. For fixed values of *c* and *s*, we substitute $$\gamma =-f'({\bar{x}})$$ into (32), and express $$\alpha ^*$$ in terms of $${\bar{x}}$$. Then we substitute $$\alpha =\alpha ^*$$ into $$(\alpha +c)f({\bar{x}})= {\bar{x}}$$ to solve for $${\bar{x}}$$, and then obtain the equilibrium, as indicated in Proposition 2. Next, we substitute $${\bar{x}}$$ into $$\gamma $$, and then compute $$\alpha ^*$$ in (32) to confirm that it is positive or check condition (34). Note that for the PS model, there are two values of $${\bar{x}}$$ and we choose the smaller one to have smaller value of $$\alpha ^*$$. By examining the crossing condition, we confirm that the assertion of Theorem 4 holds. We denote such *c* by $$c^*$$ and still call $$(c^*, \alpha ^*)$$ a HB value. These HB values $$(c^*, \alpha ^*)$$ form the *Hopf bifurcation curve* in the $$(c, \alpha )$$-plane.

According to Theorem 4, a small-amplitude periodic solution emerges, at $\alpha > \alpha ^*$ or $\alpha < \alpha ^*$ and $\alpha$ close to $\alpha ^*$, with frequency $\omega _c$ about $\omega _c^*$. We are interested in seeing how the collective frequency $\omega _c$ of oscillation varies with the coupling strength *c* and parameter $\alpha$ in system (30). Let us pick one of the HB values and denote it by $P_1=P_1(c^*, \alpha ^*)$. Recall that the Hopf bifurcation occurs at $\alpha =\alpha _0$ in the single-cell systems.

**Variations of eigenvalues** At $(c, \alpha )=P_0(0,\alpha _0$), there are two pairs of purely imaginary eigenvalues for the linearized system at $\overline{{\mathbb {X}}}(\alpha _0)$. For the identical-cell case, system (30), these two pairs coincide. It is interesting to see how the two complex-conjugate branches emanating from these two pairs move in complex plane ${{\mathbb {C}}}$, and which of them reaches the imaginary axis again at $P_1(c^*, \alpha ^*)$. As the magnitudes of the purely imaginary eigenvalues correspond to the frequencies of the individual cells at $P_0$ and coupled-cell at $P_1$, we can observe the transition of frequency from the variation of eigenvalues from $P_0$ to $P_1$. More precisely, it is interesting to see, as *c* increases from 0 so that the coupling becomes effective and a collective periodic solution is formed, the relative positions of the eigenvalues at $P_1$ with respect to the eigenvalues at $P_0$. This reflects a transition from single-cell oscillation to coupled-cell oscillation. Moreover, we can also see how the local dynamics around $\overline{{\mathbb {X}}}$ is changing from the variations of eigenvalues. Certainly, there are infinitely many paths from $P_0$ to $P_1$ in $(c, \alpha )$-plane. For simplicity and illustration, we take the line segment from $P_0$ to $P_1$ to trace the variations of eigenvalues.

To confirm that the Hopf bifurcation is supercritical so that the emergent periodic solution is stable and see whether it occurs for $\alpha > \alpha ^*$ or $\alpha < \alpha ^*$, we take another line segment in $(c, \alpha )$-plane by fixing $c=c^*$, and allowing $\alpha$ to increase from below $\alpha ^*$ to above $\alpha ^*$. We call such segment *HB course*. We can compute the eigenvalues of $J(\overline{{\mathbb {X}}};\alpha )$ to see if there is a stability switch for equilibrium $\overline{{\mathbb {X}}}$ when $\alpha$ crosses $\alpha ^*$, along such HB courses.

Let us illustrate the implementation of these ideas by the following examples. We take the same parameter values of *n* and $$k_\mathrm{H}$$ in Example 3.1, and *A* and $$ k_d$$ in Example 3.2. Recall that the frequency for the isolated ($$c=0$$) individual cell is approximately $$\omega _0 =\sqrt{3}$$, when $$\alpha $$ is close to $$\alpha _0$$. In the following example, we fix $$s=20$$. These numerical results are carried out by MATLAB programming and are consistent with the computations by software AUTO.

#### Example 4.1.1

**Fig. 5:**
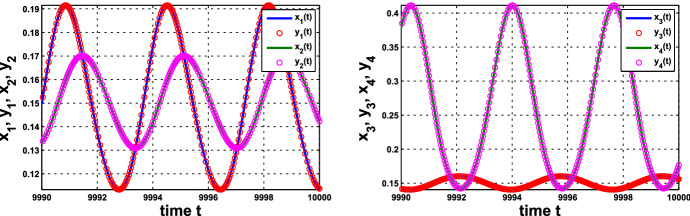
Synchronous periodic solution in Example 4.1.1(i)

**Fig. 6:**
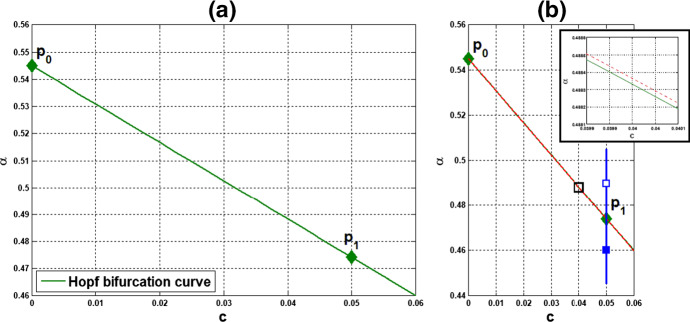
For Example 4.1.1(i): **a** HB curve comprising HB values plotted by green curve on $(c,\alpha )$-plane; $P_0$ is a HB value for the single-cell system, $P_1$ is a HB value for the coupled-cell system. **b** Line segment $P_0P_1$ in red line, the HB course through $P_1$ in blue line, from solid square to hollow square (Color figure online)

(i) Consider coupled-cell HT model (30) with parameter values $n=11$, $k_\mathrm{H}=0.136$, and $s=20$. With such parameter values, the single-cell system undergoes a Hopf bifurcation at $\alpha _0 \approx 0.54174$, as shown in Example 3.1. Through computation, the HB curve is drawn in Fig. 6. For illustration, we take $P_1(c^*=0.05, \alpha ^*=0.4742)$ on the curve. At this HB value, we compute to find $\gamma _1\approx 15.05911$, and thus confirm that condition (34) in Theorem 4 holds. Note that the line segment $P_0P_1$ lies above the HB curve, as shown in Fig. 6. We demonstrate the synchronous periodic solution at parameter values near $P_1$ in Fig. 5.

**Fig. 7:**
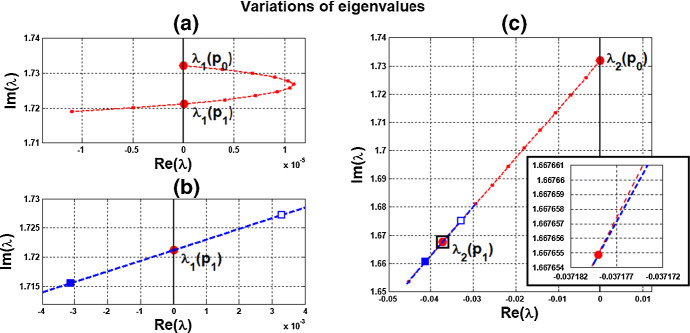
Variations of two complex eigenvalues as $(c,\alpha )$ moves along segment $P_0P_1$ (red) and the HB course (blue) in Fig. 6: **a** $\lambda _1$-branch along $P_0P_1$ reaches the imaginary axis at $P_1$, where $(c,\alpha )=(c^*, \alpha ^*)= (0.05,0.4742)$. **b** $\lambda _1$-branch along the course, from solid square to hollow square, crosses the imaginary axis at parameter value $P_1$. **c** $\lambda _2$-branches fall on the left complex plane along both $P_0P_1$ and the course. A supercritical Hopf bifurcation occurs at $\alpha ^* \approx 0.4742$ (Color figure online)

Variation of eigenvalues in ${{\mathbb {C}}}$ along the segment $P_0P_1$ is plotted by the curve in Fig. 7a, c. There are two branches of complex-conjugate eigenvalues $\lambda _1, {\overline{\lambda }}_1$ and $\lambda _2, {\overline{\lambda }}_2$, and four negative real eigenvalues. We denote by $\lambda _1(P)$ and $\lambda _2(P)$ the eigenvalues $\lambda _1$ and $\lambda _2$ at point *P*. When $c=0$, $\lambda _1(P_0)$ and $\lambda _2(P_0)$ are purely imaginary, with $\lambda _1(P_0)=\lambda _2(P_0)= \sqrt{3}~i$. As the parameters *c* and $\alpha$ vary along $P_0P_1$, the $\lambda _1$-branch moves to the right complex plane and makes a turn downward to reach the imaginary axis at parameter value at $P_1$. Notice that $\lambda _1(P_1)=i \omega _c^*$ lies below $\lambda _1(P_0)$, i.e., the collective frequency at $(c,\alpha )=(c^*, \alpha ^*)$ is smaller than the individual frequency at $(c,\alpha )=(0, \alpha _0)$. At the meantime, $\lambda _2$-branch moves to the left complex plane. To summarize, at $P_1$, there are a pair of purely imaginary eigenvalues $\lambda _1(P_1), {\overline{\lambda }}_1(P_1)$ and six eigenvalues with negative real parts, including $\lambda _2(P_1), {\overline{\lambda }}_2(P_1)$ . They are $\pm 1.721229 i$, $-0.037179 \pm 1.667655 i$, $-2.925642$, $-3.002199$, $-19.997801$, and $-20$.

**Fig. 8:**
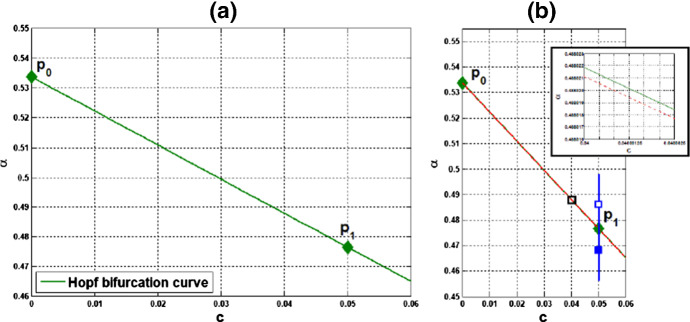
For Example 4.1.1(ii): **a** HB curve comprising HB values plotted by green curve on $(c,\alpha )$-plane; $P_0$ is a HB value for the single-cell system, $P_1$ is a HB value for the coupled-cell system. **b** Line segment $P_0P_1$ in red line; the HB course through $P_1$ in blue line, from solid square to hollow square (Color figure online)

**Fig. 9:**
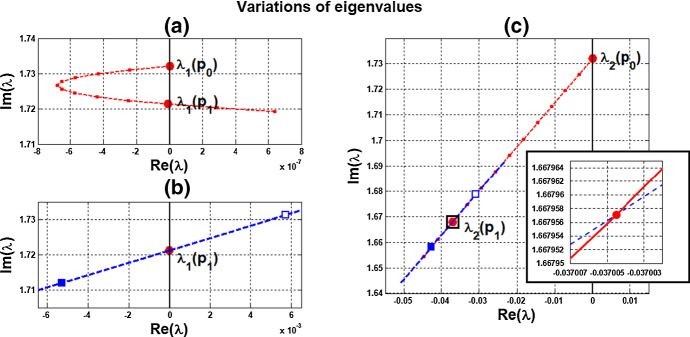
Variations of two complex eigenvalues as $(c,\alpha )$ moves along segment $P_0P_1$ (red) and the course (blue) in Fig. 8: **a** $\lambda _1$-branch along $P_0P_1$ reaches the imaginary axis at parameter value $P_1(0.05,0.4766)$. **b** $\lambda _1$-branch along the course, from solid square to hollow square, crosses the imaginary axis at $P_1$. **c** $\lambda _2$-branches fall on the left complex plane along both $P_0P_1$ and the course. A supercritical Hopf bifurcation occurs at $\alpha ^* \approx 0.4766$ (Color figure online)

Variation of eigenvalues in ${{\mathbb {C}}}$ along the HB course in Fig. 6 is plotted by the curve in Fig. 7b, c. We thus confirm that a periodic solution emerges as $\alpha$ passes slightly over $\alpha ^*$, with frequency $\omega _c$ close to $\omega _c^*\approx 1.721229$, by Theorem 4. Our numerical simulation shows that for $\alpha$ slightly above $\alpha ^*$, the collective frequency is $\omega _c\approx 1.71999$ for $(c, \alpha ) = (0.05, 0.475)$.

(ii) Consider coupled-cell PS model (30) with parameter values $A=0.0659$, $k_d=0.00001$, and $s=20$. With such parameter values, the single-cell system undergoes a Hopf bifurcation at $\alpha _0 \approx 0.533809$, as shown in Example 3.2. We compute and draw the HB curve in Fig. 8. For illustration, we take $P_1(c^*=0.05, \alpha ^*=0.4766)$ in the curve. We compute to find $\gamma _2\approx 14.99108$, and then confirm the condition (34) in Theorem 4. Note that this line segment $P_0P_1$ lies below the HB curve, as shown in Fig. 8. Variations of eigenvalues along $P_0P_1$ are depicted by the curves in Fig. 9a, c. When $c=0$, $\lambda _1(P_0)$ and $\lambda _2(P_0)$ are purely imaginary, with $\lambda _1(P_0)=\lambda _2(P_0)=\sqrt{3}~i$. As the parameters *c* and $\alpha$ vary along $P_0P_1$, $\lambda _1$-branch moves to the left complex plane, and makes a turn downward to reach the imaginary axis at parameter value $P_1$. As $\lambda _1(P_1)=i \omega _c^*$ lies below $\lambda _1(P_0)$, we see that the collective frequency at $(c,\alpha )=(c^*, \alpha ^*)$ is smaller than the individual frequency at $(c,\alpha )=(0, \alpha _0)$. Meanwhile, $\lambda _2$-branch moves to the left complex plane. At $P_1$, the eigenvalues are computed as $\pm 1.721278i$, $-0.037004\pm 1.667957 i$, $-2.925991$, $-3.002189$, $-19.997811$, and $-20$.

Variations of eigenvalues along the HB course are shown by the curves in Fig. 9b, c. According to Theorem 4, a periodic solution emerges for $\alpha >\alpha ^*$ and close to $\alpha ^*$, with frequency $\omega _c$ close to $\omega _c^* \approx 1.721278$. Our numerical simulation reveals that for $\alpha$ slightly above $\alpha ^* =0.4766$, the collective frequency is $\omega _c\approx 1.72115$ at $(c, \alpha ) =(0.05, 0.498)$.

We carry out similar computation for the system at the other HB values in Figs. 6 and 8. The scenarios, the variations of eigenvalues along line segments from $P_0$, resemble the ones for $P_0P_1$ in Figs. 7a, c and 9a, c. The variation of eigenvalues along each associated HB course is also similar to the one for $P_1$ in Figs. 7b, c and 9b, c.

Let us make a comparison about variations of eigenvalues along the line segments between the coupled-cell HT model and PS model. In the HT model, $\lambda _1$-branch moves to the right half plane of ${{\mathbb {C}}}$ and then makes a turn downward to reach the imaginary axis at $P_1$, whereas $\lambda _2$-branch remains on the left complex plane. In the PS model, both $\lambda _1$-branch and $\lambda _2$-branch move into the left complex plane. Then, $\lambda _1$-branch makes a turn downward and reaches the imaginary axis at $P_1$, and $\lambda _2$-branch continues to stay in the left complex plane. The basic reason for the different movement of $\lambda _1$-branch is that line segment $P_0P_1$ is above the HB curve for the HT model, whereas it lies below the HB curve in the PS model, as indicated in Figs. 6 and 8, respectively. Accordingly, there is a difference on the transition of the local dynamics around equilibrium $\overline{{\mathbb {X}}}$ between these two models. For the HT model, the dimension of the unstable manifold of $\overline{{\mathbb {X}}}$ increases from zero to two (the dimension of the stable manifold from four to six) when $(c, \alpha )$ moves from $P_0$ along $P_0P_1$, before reaching $P_1$. On the other hand, for the PS model, the dimension of the stable manifold of $\overline{{\mathbb {X}}}$ increases from four to eight when $(c, \alpha )$ moves from $P_0$, before reaching $P_1$. Other than that distinction, both models undergo a supercritical Hopf bifurcation at $\alpha =\alpha ^*$. As the stable periodic solutions of the coupled-cell systems emerge, the collective frequency at each $(c, \alpha )=(c^*, \alpha ^*)$ is smaller than the individual frequencies at $(c, \alpha )=(0, \alpha _0)$ in both models. Indeed, the purely imaginary eigenvalues $\lambda _1(P_1)$ lie below the purely imaginary eigenvalues $\lambda _1(P_0)$ in both models.

#### Remark 3

The intercellular coupling is relatively fast, and we took $s=20$ in the above examples. We observe from system (30) that $x_4$ and $y_4$ may be regarded as being in quasi-steady state, i.e., $x_4(t) \approx f(x_3(t))$, $y_4(t) \approx f(y_3(t))$, and thus the first equation is approximately

$$\begin{aligned} {\dot{x}}_1 = ( \alpha +c) f(x_3) - x_1. \end{aligned}$$

Recalling the Hopf bifurcation in the single-cell system (16), we see that the occurrence of the Hopf bifurcation in the coupled system (30) is approximately along the curve $\alpha +c= \alpha _0$ where $\alpha _0 \cong 0.545174$ in Example 4.1.1(i), and $\alpha _0 \cong 0.533809$ in Example 4.1.1(ii). This explains why the HB curves in Figs. 6 and 8 are almost straight lines and that the frequency decreases with increasing coupling strength is linked to the frequency decrease with increasing $\alpha$ in the single-cell system.

#### Remark 4

**Fig. 10:**
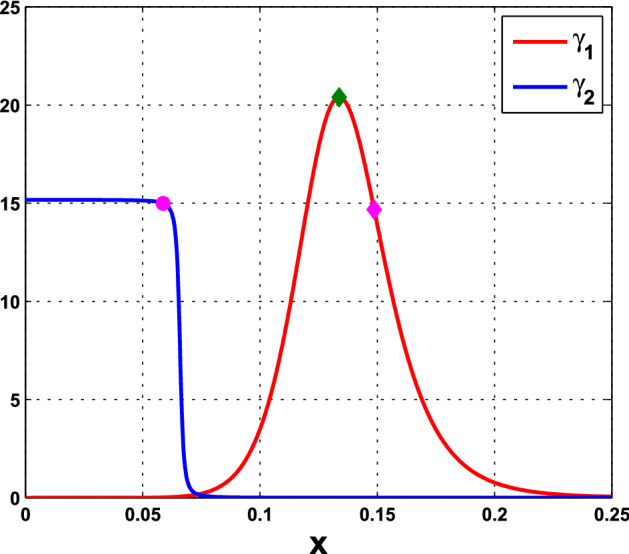
The graphs of $\gamma _1$ and $\gamma _2$, for the parameter values in Example 4.1.1, with $n=11$, $k_\mathrm{H}=0.136$, $A=0.0659$, $k_d=0.00001$. The green diamond represents $x_{\mathrm{M}}\approx 0.133764$, the magenta diamond represents ${\bar{x}}\approx 0.148684$ when $c=0$ in the HT model, and the magenta circle represents ${\bar{x}}\approx 0.058730$ when $c=0$ in the PS model (Color figure online)

(i) It is seen from (32) that the HB value $\alpha ^*$ for the coupled-cell system is smaller than the HB value $\alpha _0$ for the single-cell system (when $c=0$). Based on the Hopf bifurcation theorem, this order provides a potential parameter regime where oscillations exist in both single-cell and coupled-cell systems, as well as a potential regime where oscillations exist in the coupled-cell system, but not in the single-cell system.
(ii) We observe from (32) that $\alpha ^* \rightarrow \infty$ as $\gamma \rightarrow 0$. That is, the Hopf bifurcation does not occur at the flat parts of the repression functions $f_1$ and $f_2$ (small values of $\gamma _1$ and $\gamma _2$), see Fig. 10. On the other hand, the Hopf bifurcation occurs when $\gamma$, the magnitude of $f'({\bar{x}})$, is lower than the bound in (34). This condition was actually derived to guarantee that $\alpha ^*$ is positive. This bound is large when *c* is small. In our Example 4.1.1, the bound is above the maximal values of $\gamma _1$ and $\gamma _2$, and thus, condition (34) is automatically met.
(iii) From Theorem  4, we see that frequency $\omega _c^*$ decreases as $\gamma$ increases. That is, in contrast to the single-cell case, the frequency of the periodic solution near the HB value distinguishes between $\gamma = \gamma _1$ and $\gamma = \gamma _2$, and hence the repression function $f_1$ from $f_2$. From the graphs of $\gamma _1$ and $\gamma _2$ in Fig. 10, we see that while $\gamma _1$ has larger maximum value, $\gamma _2$ has wider range of large values. Figure 10 is depicted with the data in Example 4.1.1. It is indirect to track the value of $\gamma$, as it depends on ${\bar{x}}$ which is determined by the parameter values. For the HT model, we may compare the point $x_{\mathrm{M}}$ where $\gamma _1$ attains its maximum value with the component ${\bar{x}}$ of the equilibrium at $c=0$, that is,
  36 $$\begin{aligned} x_{\mathrm{M}}:= k_\mathrm{H}\left( \frac{n-1}{n+1}\right) ^{\frac{1}{n}}, ~{\bar{x}} = k_\mathrm{H}\left( \frac{8}{n-8}\right) ^{\frac{1}{n}}. \end{aligned}$$
  Their distance can be measured from
  37 $$\begin{aligned} {\bar{x}}^n - x_{\mathrm{M}}^n = -\frac{k_\mathrm{H}^n n(n-17)}{(n-8)(n+1)}. \end{aligned}$$
  When the coupling strength *c* is small, ${\bar{x}}$ in the coupled system is close to ${\bar{x}}$ in (36), and the quantity in (37) estimates how far the point ${\bar{x}}$ that $\gamma _1$ takes on is away from $x_{\mathrm{M}}$, where the maximum value of $\gamma _1$ is attained. Note that for $n<17$, ${\bar{x}}$ lies on the right hand side of $x_{\mathrm{M}}$, whereas it is reverse for $n>17$. In addition, we see that the gap between $x_{\mathrm{M}}$ and ${\bar{x}}$ can be enlarged, by choosing larger $k_\mathrm{H}$ and *n*. The corresponding frequencies $\omega _c^*$ will then further distinguish the repression function $f_1$ from $f_2$. The analysis for the graphs of $\gamma _1$ and $\gamma _2$ is arranged in Appendix C. From the expression for frequency $\omega _c^*$, we also see that large coupling strength *c* enhances the effect from $\gamma$, whereas large intercellular coupling time scale *s* suppresses the factor from $\gamma$. In Appendix D, we provide more numerics for the values of ${\bar{x}}$, $\gamma _1, \gamma _2$, and the corresponding frequencies $\omega _c^*$, along the HB curves for the cases with $s=10$, and $n=18$.

What are discussed in Remark 4 are about the scenario at the HB points and HB values. To extend the understanding of the frequency, we perform further numerical simulations and follow the continuation from the HB values. We are interested in comparing the collective frequency $\omega _c$ and the average frequency $\omega _{\mathrm{Ave}}$ of the individual cells. For system (30) comprising identical cells, the average frequency coincides with the individual frequency. To this end, we need to choose parameter values at which the periodic solutions exist for both of the single-cell and coupled-cell systems. In addition, to make comparison, we consider the same values of $\alpha$ in both single-cell and coupled-cell systems. As mentioned in Remark 4(i), the HB value $\alpha _0$ for the single-cell system is larger than the HB value $\alpha ^*$ for the coupled-cell system. This can also be seen in Figs. 6 and  8. For $\alpha$ slightly larger than $\alpha _0$ (resp., $\alpha ^*$), the stable periodic solutions emerge in the single-cell systems (resp., coupled-cell systems), so that we can compute the average frequency (resp., collective frequency). For those values of $\alpha$ which deviate farther from $\alpha _0$ (resp., $\alpha ^*$), the stable periodic solutions for the single-cell system (resp., coupled-cell systems) may persist, following the continuation of bifurcating periodic solutions from the Hopf bifurcation.

The following example illustrates a comparison between the average frequencies and the collective frequencies.

#### Example 4.1.2

**Fig. 11:**
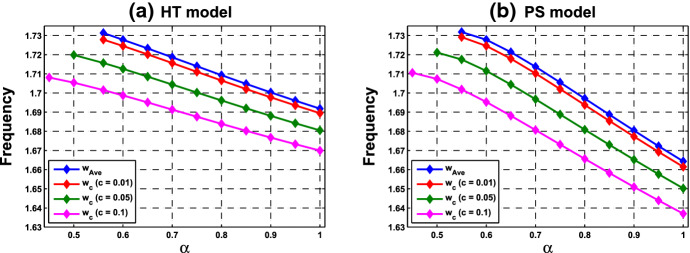
Collective frequencies $\omega _c$ of the periodic solutions for the coupled-cell systems and average frequencies $\omega _{\mathrm{Ave}}$ for the periodic solutions of the single-cell systems, corresponding to increasing value of $\alpha$, at $c=0.01, 0.05, 0.1$, in **a** the HT model, **b** the PS model, with the values of the other parameters given in Examples 4.1.1 and 4.1.2, respectively

For increasing values of $\alpha >\alpha _0$, we compute the average frequency from the frequencies of individual cells in Examples 3.1, 3.2. For each of $c^*=0.01, 0.05, 0.1$ and its corresponding $\alpha ^*$, with increasing values of $\alpha >\alpha ^*$, by solving the ODE system numerically, we observe the periodic solutions of coupled-cell system (30) at these parameter values, and identify the frequency for each of these periodic solutions. The computed collective frequencies of these periodic solutions are plotted in Fig. 11. It can be seen that, in both HT and PS models, the frequency decreases as $\alpha$ increases, and it drops faster in the PS model. Notably, the leftmost points of the plots in Fig. 11 correspond to the average frequency about $\sqrt{3}$ at $\alpha$ near $\alpha _0$, and the collective frequencies at $\alpha$ near each $\alpha ^*$, respectively. Moreover, for each fixed $\alpha$, the collective frequency decreases as the coupling strength increases. These computations all show that the collective frequency is smaller than the average of individual frequencies (when $c=0$), and are close to the average frequency when *c* is small.

#### Remark 5

In system (29), the synchronous set ${{{\mathcal {S}}}}:=\{M_i=M_j, P_i=P_j, R_i=R_j, V_i=V_j: 1\le i,j \le N \}$ is invariant. Indeed, $M_i(t)=M(t), P_i(t)=P(t), R_i(t)=R(t), V_i(t)=V(t)$, $i=1, \ldots , N$, will constitute a solution of (29) if (*M*(*t*), *P*(*t*), *R*(*t*), *V*(*t*)) is a solution of

38 $$\begin{aligned} \left\{ \begin{aligned} \displaystyle {\dot{M}}&= f(R) - M +c V \\ \displaystyle {\dot{P}}&= M - P \\ \displaystyle {\dot{R}}&= P - R \\ \displaystyle {\dot{V}}&= s[f(R)-V]. \end{aligned} \right. \end{aligned}$$

Accordingly, we can study the dynamics of system (29) restricted to the synchronous set ${{{\mathcal {S}}}}$. That is, we can consider the *N*-cell analog of system (30) on its synchronous set, which is a four-dimensional system like (38). The characteristic polynomial for the linearization of the restricted system at $\bar{\mathbf{x}}$ is exactly $\varDelta _+(\lambda )$. Recall that the bifurcation analysis in this section unfolds from the roots of $\varDelta _{+}(\lambda )=0$. Thus, Theorem 4 and the analysis herein remains valid for the restricted system. And the bifurcating periodic solution $(x_1(t), x_2(t), x_3(t), x_4(t))$ for the restricted system extends, with its copies $(x_1(t), x_2(t), x_3(t), x_4(t))^N$, to a synchronous periodic solution for the *N*-cell system.

### Two Nonidentical Cells

In this section, we consider the coupled-cell system consisting of two nonidentical cells, by adding a scaling factor $\sigma$ into the second cell:

39 $$\begin{aligned} \left\{ \begin{aligned} {\dot{x}}_1&= \displaystyle \alpha f(x_3) - x_1 + \frac{c}{2}(x_4 + y_4) \\ \displaystyle {\dot{x}}_2&= x_1 - x_2 \\ \displaystyle {\dot{x}}_3&= x_2 - x_3 \\ \displaystyle {\dot{x}}_4&= s[f(x_3)-x_4] \\ {\dot{y}}_1&= \displaystyle \sigma (\alpha f(y_3) - y_1) + \frac{c}{2}(y_4 + x_4) \\ \displaystyle {\dot{y}}_2&= \sigma (y_1 - y_2) \\ \displaystyle {\dot{y}}_3&= \sigma (y_2 - y_3) \\ \displaystyle {\dot{y}}_4&= s[f(y_3)-y_4]. \end{aligned} \right. \end{aligned}$$

When $c=0$, two cells are decoupled. We consider the parameter values at which each cell oscillates at its own frequency. As *c* increases from 0, the cells become coupled, yet it is not assured whether a collective periodic solution is then generated. The periodic solution, if exists, is no longer synchronous, and becomes phase-locked. Indeed, as system (39) comprises nonidentical subsystems, the synchronous set is no longer invariant and the solutions are asynchronous in general. Therefore, for different components of solutions to attain (or compromise to reach) the same period, it is quite natural that there exists a phase difference between the corresponding components of a collective periodic solution.

We again choose $\alpha$ as the bifurcation parameter and employ Theorem 1 to analyze the existence of periodic solution for system (39). Note that when $c=0$, system (39) reduces to two decoupled subsystems

40 $$\begin{aligned} \left\{ \begin{aligned} \displaystyle {\dot{x}}_1&= \alpha f(x_3) - x_1 \\ \displaystyle {\dot{x}}_2&= x_1 - x_2 \\ \displaystyle {\dot{x}}_3&= x_2 - x_3, \end{aligned} \right. \end{aligned}$$

and

41 $$\begin{aligned} \left\{ \begin{aligned} \displaystyle {\dot{y}}_1&= \sigma (\alpha f(y_3) - y_1) \\ \displaystyle {\dot{y}}_2&= \sigma (y_1 - y_2) \\ \displaystyle {\dot{y}}_3&= \sigma (y_2 - y_3). \end{aligned} \right. \end{aligned}$$

Components $x_4(t), y_4(t)$ can be obtained by integrating the fourth and eighth components of system (39) once $x_3(t), y_3(t)$ are solved. According to the discussions in Sect. 3, both of systems (40) and (41) undergo a Hopf bifurcation at $\alpha =\alpha _0$, with respective frequency

$$\begin{aligned} \omega _1^*= \sqrt{3}, ~ \omega _2^*= \sigma \sqrt{3}. \end{aligned}$$

The positive equilibrium $\overline{{\mathbb {X}}}=(\bar{{\mathbf{x}}},\bar{{\mathbf{y}}})= ({\bar{x}}_1,{\bar{x}}_2,{\bar{x}}_3,{\bar{x}}_4,{\bar{y}}_1,{\bar{y}}_2,{\bar{y}}_3,{\bar{y}}_4)$ of system (39) satisfies ${\bar{x}}_1={\bar{x}}_2={\bar{x}}_3$, $f({\bar{x}}_3)={\bar{x}}_4$, and ${\bar{y}}_1={\bar{y}}_2={\bar{y}}_3$, $f({\bar{y}}_3)={\bar{y}}_4$, and

42 $$\begin{aligned} \left\{ \begin{aligned} \alpha f({\bar{x}}_3)-{\bar{x}}_3 +\frac{c}{2}[f({\bar{x}}_3)+f({\bar{y}}_3)]&=0\\ \sigma [\alpha f({\bar{y}}_3)-{\bar{y}}_3] +\frac{c}{2}[f({\bar{x}}_3)+f({\bar{y}}_3)]&=0. \end{aligned} \right. \end{aligned}$$

The existence of such heterogenous equilibrium is shown in Appendix E. The Jacobian matrix associated with the linearization of system (39) at $\overline{{\mathbb {X}}}$ is

$$\begin{aligned} J(\overline{{\mathbb {X}}};\alpha ) := \left[ \begin{array}{cccccccc} ~-1 &{} 0 &{} -\alpha \gamma &{} \displaystyle \frac{c}{2} &{} 0 &{} 0 &{} 0 &{} \displaystyle \frac{c}{2}~ \\ ~1 &{} -1 &{} 0 &{} 0 &{} 0 &{} 0 &{} 0 &{} 0~ \\ ~0 &{} 1 &{} -1 &{} 0 &{} 0 &{} 0 &{} 0 &{} 0~ \\ ~0 &{} 0 &{} -s\gamma &{} -s &{} 0 &{} 0 &{} 0 &{} 0~ \\ ~0 &{} 0 &{} 0 &{} \displaystyle \frac{c}{2} &{} -\sigma &{} 0 &{} -\sigma \alpha {\tilde{\gamma }} &{} \displaystyle \frac{c}{2}~ \\ ~0 &{} 0 &{} 0 &{} 0 &{} \sigma &{} -\sigma &{} 0 &{} 0~ \\ ~0 &{} 0 &{} 0 &{} 0 &{} 0 &{} \sigma &{} -\sigma &{} 0~ \\ ~0 &{} 0 &{} 0 &{} 0 &{} 0 &{} 0 &{} -s{\tilde{\gamma }} &{} -s~ \end{array}\right] , \end{aligned}$$

where $\gamma :=-f'({\bar{x}}_3)$, and ${\tilde{\gamma }}:=-f'({\bar{y}}_3)$. We denote the characteristic polynomial $\varDelta (\lambda ):=\mathrm{det}(\lambda I-J(\overline{{\mathbb {X}}};\alpha ))$ by

43 $$\begin{aligned} \lambda ^8+b_1\lambda ^7+b_2\lambda ^6+b_3\lambda ^5+b_4\lambda ^4 +b_5\lambda ^3+b_6\lambda ^2+b_7\lambda +b_8, \end{aligned}$$

where the coefficients $b_i$ depend on $s, \sigma , c$, and $\gamma , {\tilde{\gamma }}$, which in turn depend on ${\bar{x}}_3$ and ${\bar{y}}_3$. In contrast to the discussion on systems comprising two identical cells, herein, we could not factorize this polynomial $\varDelta (\lambda )$. To apply the Hopf bifurcation theory, we look for the values of $\alpha ^*$ and the values for the other parameters, which satisfy the conditions of Theorem 1 with $m=8$. We can also observe that $\alpha ^* \rightarrow \infty$ as $\gamma \rightarrow 0$, for almost all parameter values. That is, the Hopf bifurcation does not occur at the flat parts of $f_1$ and $f_2$. This is due to that $\alpha$ always appears together with $\gamma$ or ${\tilde{\gamma }}$ in $J(\overline{{\mathbb {X}}};\alpha )$.

The following example is parallel to the discussions in Example 4.1.1. The computation process is similar, but slightly different, as we do not have an explicit expression of $\alpha ^*$ like (32) for the identical-cell case, nor a concrete formula like the one in Theorem 4 to resort to. We solve numerically for $\alpha ^*$ and ${\bar{x}}_3$ and ${\bar{y}}_3$ which satisfy $D_7(\alpha ^*)=0$ and (42). Then, we confirm that the other conditions of Theorem 1 are met. Again, for parameters of the individual cells, we take the same values as in Examples 3.1 and 3.2. For the scaling factor in the second cell, we take $\sigma = 1.05$.

#### Example 4.2

**Fig. 12:**
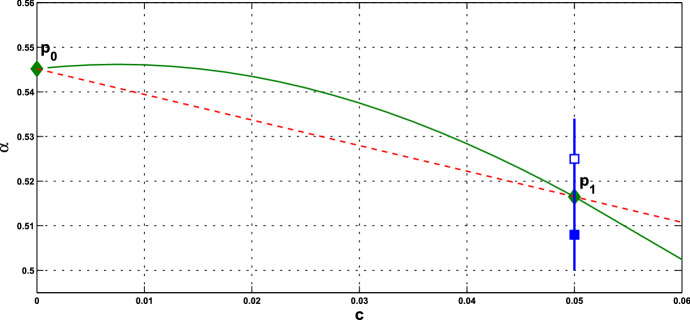
For Example 4.2.1(i): The HB curve plotted in green curve on $(c,\alpha )$-plane; $P_0$ is a HB value for the single-cell system. $P_1$ is a HB value for the coupled-cell system; line segment $P_0P_1$ in red line, the HB course in blue line (Color figure online)

**Fig. 13:**
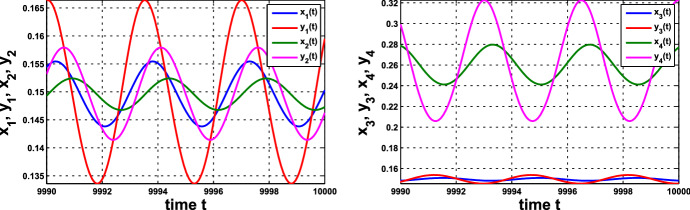
Phase-locked periodic solution, with phase difference approximately 0.310757 (the difference of *t* between the peaks of $x_j$ and $y_j$), in Example 4.2(i)

**Fig. 14:**
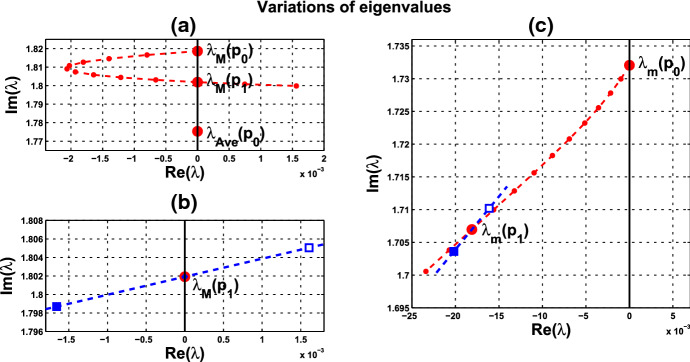
Variations of two complex eigenvalues as $(c,\alpha )$ moves along segment $P_0P_1$ (red) and the HB course (blue) in Fig. 12: **a** Along $P_0P_1$, $\lambda _{\mathrm{M}}$-branch reaches the imaginary axis again at $P_1(0.05,0.5165)$. **b** Along the course, from solid square to hollow square, $\lambda _{\mathrm{M}}$-branch crosses the imaginary axis at $P_1$. (c) $\lambda _{\mathrm{m}}$-branches fall on the left complex plane along both of $P_0P_1$ and the course. A supercritical Hopf bifurcation occurs when $\alpha ^* \approx 0.5165$. $\lambda _{\mathrm{Ave}}(P_0):= [\lambda _{\mathrm{M}}(P_0)+ \lambda _{\mathrm{m}}(P_0)]/2$ (Color figure online)

(i) Consider the coupled-cell HT model (39) with parameter values $n=11$, $k_\mathrm{H}=0.136$, $s=20$, and $\sigma = 1.05$. The HB curve is plotted in Fig. 12. For illustration, we take one of the HB values $P_1(c^*=0.05, \alpha ^*=0.5165)$. We demonstrate the periodic solution at parameter values near $P_1$ in Fig. 13, which appears to be phase-locked (asynchronous). Variations of the eigenvalues along the line segment $P_0P_1$ are shown by the curves in Fig. 14a, c. There are two branches of complex-conjugate eigenvalues $\lambda _{\mathrm{M}}, {\overline{\lambda }}_{\mathrm{M}}$ and $\lambda _{\mathrm{m}}, {\overline{\lambda }}_{\mathrm{m}}$, and four negative real eigenvalues. We denote by $\lambda _{\mathrm{M}}(P)$ and $\lambda _{\mathrm{m}}(P)$ the eigenvalues of $\lambda _{\mathrm{M}}$ and $\lambda _{\mathrm{m}}$ at point *P*. When $c=0$, $\lambda _{\mathrm{M}}(P_0)$ and $\lambda _{\mathrm{m}}(P_0)$ are purely imaginary, with $\lambda _{\mathrm{m}}(P_0)= \sqrt{3}~i$, $\lambda _{\mathrm{M}}(P_0)=1.05\sqrt{3}~i$. As *c* and $\alpha$ vary along $P_0P_1$, the $\lambda _{\mathrm{M}}$-branch moves to the left complex plane, and makes a turn downward to reach the imaginary axis at $P_1$. Observe that $\lambda _{\mathrm{M}}(P_1)=i\omega _c^*$ lies below $\lambda _{\mathrm{M}}(P_0)$, i.e., the magnitude of $\lambda _{\mathrm{M}}(P_1)$ is smaller than that of $\lambda _{\mathrm{M}}(P_0)$. On the other hand, along $P_0P_1$, $\lambda _{\mathrm{m}}$-branch moves to and stays in the left complex plane. That is, at $P_1$, there are a pair of purely imaginary eigenvalues $\lambda _{\mathrm{M}}(P_1), {\overline{\lambda }}_{\mathrm{M}}(P_1)$ and six eigenvalues with negative real parts, including $\lambda _{\mathrm{m}}(P_1)$, namely $\pm 1.801918i$, $-0.018114 \pm 1.706965$, $-2.974896$, $-3.141083$, $-19.997793$, $-20$.

**Fig. 15:**
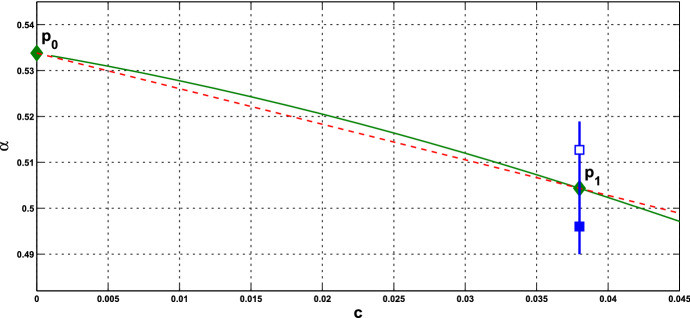
For Example 4.2(ii): The HB curve plotted by green curve on $(c,\alpha )$-plane; $P_0$ is a HB value for the single-cell system, $P_1$ is a HB value for the coupled-cell system; $P_0P_1$ in red line, the HB course in blue line (Color figure online)

**Fig. 16:**
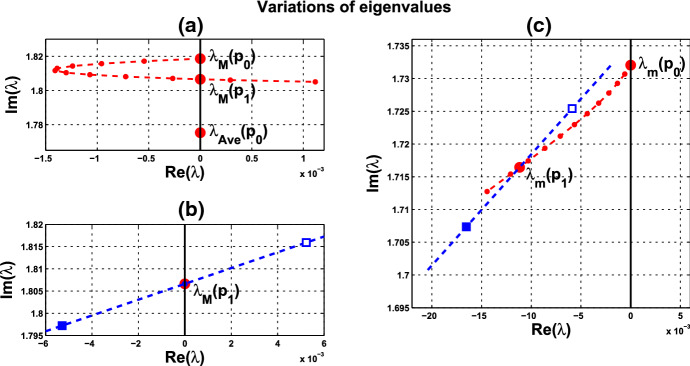
Variations of two complex eigenvalues as $(c,\alpha )$ moves along segment $P_0P_1$ (red) and the HB course (blue) in Fig. 15: **a** $\lambda _{\mathrm{M}}$-branch along $P_0P_1$ reaches the imaginary axis again at $P_1(0.038,0.5043)$. **b** $\lambda _{\mathrm{M}}$-branch along the course, from solid square to hollow square, crosses the imaginary axis at $P_1$. **c** The branches of $\lambda _{\mathrm{m}}$ fall on the left complex plane along both of $P_0P_1$ and the course. A supercritical Hopf bifurcation occurs when $\alpha ^* \approx 0.5043$. $\lambda _{\mathrm{Ave}}(P_0):= [\lambda _{\mathrm{M}}(P_0)+ \lambda _{\mathrm{m}}(P_0)]/2$ (Color figure online)

Variations of the eigenvalues along the HB course are shown by the curves in Fig. 14b, c. As $\alpha$ increases from below $\alpha ^*$ to above $\alpha ^*$, the stable equilibrium $\overline{{\mathbb {X}}}$ becomes unstable and a stable periodic solution emerges, with frequency $\omega _c$ close to $\omega _c^* \approx 1.801918$.

(ii) Consider the coupled-cell PS model (39) with parameter values $A=0.0659$, $k_d=0.00001$, $s=20$, and $\sigma =1.05$. We compute and draw the HB curve in Fig. 15. We take the HB value $P_1(c^*=0.038, \alpha ^*=0.5043)$. Variations of eigenvalues along the segment $P_0P_1$ are shown by the curves in Fig. 16a, c. The scenario is similar to the one in Fig. 14a, c.

Variations of eigenvalues along the HB courses are shown in Fig. 16b, c. As $\alpha$ increases from below $\alpha ^*$ to above $\alpha ^*$, the stable equilibrium $\overline{{\mathbb {X}}}$ becomes unstable and a stable periodic solution emerges, with frequency $\omega _c$ about $\omega _c^* \approx 1.806613$.

The scenarios at the other Hopf bifurcation values $(c^*, \alpha ^*)$ in Fig. 12 (resp. Fig. 15) are qualitatively similar to the ones in Fig. 14 (resp. Fig. 16). For each of $c^*=0.05, 0.075, 0.1$ in the HT model and $c^*=0.04, 0.05, 0.1$ in the PS model, there corresponds an $\alpha ^*$. We further trace the frequency of oscillation as $\alpha$ increases over each $\alpha ^*$ by solving system (39) numerically. The results are plotted in Fig. 18, where the average frequency is $\omega _{\mathrm{Ave}} =(\omega _1+\omega _2)/2$, with $\omega _1$, $\omega _2$ the frequencies of the first cell and second cell, respectively, when isolated. The leftmost points of the plots indicate the values $\alpha _0$ for $c=0$, and $\alpha ^*$ for each $c^*$, which are different.

**Fig. 17:**
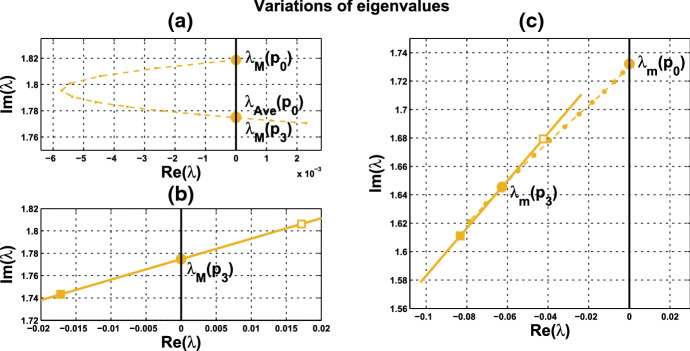
Variations of complex eigenvalue **a** $\lambda _{\mathrm{M}}$ along $P_0P_3$. **b** $\lambda _{\mathrm{M}}$ along the associated HB course. **c** $\lambda _{\mathrm{m}}$ along $P_0P_1$ (dotted curve) and the course (solid curve) in Fig. 15, where $P_3$ is at $(c, \alpha )=(c^*, \alpha ^*)= (0.1, 0.4325)$; $\lambda _{\mathrm{M}}(P_3) = 1.774746i$, $\lambda _{\mathrm{Ave}}(P_0) = 1.775352i$

**Fig. 18:**
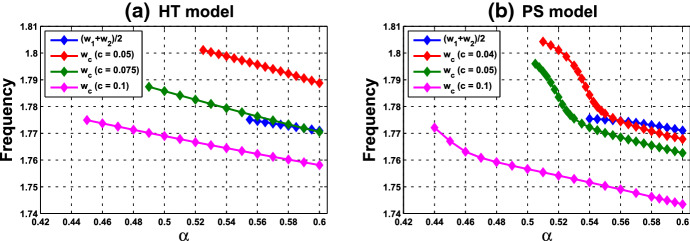
Average frequency $\omega _{\mathrm{Ave}} = (\omega _1+\omega _2)/2$ with respect to $\alpha$ at $c=0$, and collective frequency with respect to $\alpha$ at fixed *c* in **a** the HT model with $c=0.05, 0.075, 0.1$, and **b** the PS model with $c=0.04, 0.05, 0.1$, where $\omega _1$ and $\omega _2$ are the frequencies for the two single cells corresponding to the value of $\alpha$

#### Remark 6

(i) We note that the HB curve is not connected to point $P_0$ in Figs. 12 and 15, since it is not known if the Hopf bifurcation can occur at arbitrarily small coupling strength *c*.
(ii) We observe from Figs. 12 and  15 that the HB values $\alpha ^*$ for the HT model are larger than the ones for the PS model, at the same coupling strength *c*. This difference is also shown in Fig. 18, at the leftmost endpoints of these plots at coupling strength $c=0.05$ and $c=0.1$. Thus, for an $\alpha$ larger than $\alpha ^*$ for the HT model, it is even larger than $\alpha ^*$ for the PS model. This could be a factor that the frequency for the PS model drops faster than the HT model as $\alpha$ increases over $\alpha ^*$.

### Collective Frequency Versus Average Frequency

With the discussions in Sects. 4.1 and 4.2, we like to observe further how the collective frequency of coupled-cell systems is compared to the average frequency of individual cells when isolated.

For coupled-cell system (30) comprising two identical cells, the average frequency is the individual frequency. We have seen in Example 4.1.2 and Fig. 11 that the collective frequencies are lower than the average frequencies for both HT model and PS model. In addition, the collective frequencies are close to the average frequencies when the coupling strength *c* is small. This result also holds for coupled-cell system (29) with *N* identical cells, as mentioned in Remark 5.

For coupled system (39) consisting of two nonidentical cells with individual frequencies $\omega _1$ and $\omega _2$ when isolated, the average frequency is $\omega _{\mathrm{Ave}}:=(\omega _1+ \omega _2)/2$. To indicate dependence on $\alpha$, we denote $\omega _1= \omega _1(\alpha ), \omega _2= \omega _2(\alpha )$, and $\omega _1^*:= \omega _1^*(\alpha _0), \omega _2^*:= \omega _2(\alpha _0)$, where $\alpha _0$ is the bifurcation value for the single cell. Note that $\omega _2^*=\sigma \omega _1^*$. In Examples 4.2(i) (HT model) and 4.2(ii) (PS model), we have seen that both $\lambda _{\mathrm{M}}(P_1)$ lie below $\lambda _{\mathrm{M}}(P_0)$, with $\sigma =1.05$, $\lambda _{\mathrm{M}}(P_0)=i \omega _2^*$ and $\lambda _{\mathrm{m}}(P_0)=i \omega _1^*$, $\lambda _{\mathrm{M}}(P_1)=i \omega _c^*$. That is, when the collective periodic solution is formed at a HB value $(c^*, \alpha ^*)$, the collective frequency $\omega _c^*$ drops below the larger individual frequency $\omega _2^*$ at $c=0$ (decoupled) and $\alpha$ near $\alpha _0$. However, on the one hand, the average frequency at $c=0$ and $\alpha$ near $\alpha _0$ is about $\omega _{\mathrm{Ave}}^*:=(\omega _1^*+ \omega _2^*)/2$. On the other hand, the comparison of the collective frequency $\omega _c=\omega _c(\alpha )$ (at $c>0$) and the average frequency $\omega _{\mathrm{Ave}}=\omega _{\mathrm{Ave}}(\alpha )$ (at $c=0$) should be made at the same parameter values, including $\alpha$. Notably, our notation means $\omega _c^*=\omega _c(\alpha ^*)$, $\omega _{\mathrm{Ave}}^*=\omega _{\mathrm{Ave}}(\alpha _0)$.

Our strategy of comparison starts with comparing $\lambda _{\mathrm{M}}(P_j), \lambda _{\mathrm{m}}(P_j)$, $j \in {{\mathbb {N}}}$, with $\lambda _{\mathrm{M}}(P_0)$, $\lambda _{\mathrm{m}}(P_0)$, and $\lambda _{\mathrm{Ave}}(P_0):= [\lambda _{\mathrm{M}}(P_0)+ \lambda _{\mathrm{m}}(P_0)]/2$, where $P_j$ is a HB value. That is, we compare the eigenvalues at $(c, \alpha )=(c^*, \alpha ^*)$ with the ones at $(c, \alpha )=(0,\alpha _0)$. For example, for the case $\alpha ^*<\alpha _0$, if $\lambda _{\mathrm{M}}(P_j)$ lies below $\lambda _{\mathrm{Ave}}(P_0)$ on the imaginary axis of ${{\mathbb {C}}}$, i.e., $\omega _c^*< \omega _{\mathrm{Ave}}^*$, and if $\omega _c=\omega _c(\alpha )$ decreases as $\alpha$ increases (with fixed $c=c^*$), then we are sure that $\omega _c< \omega _c^*<\omega _{\mathrm{Ave}}$ at $\alpha$ slightly larger than $\alpha _0$ (with fixed $c=c^*$), should the periodic solution exist up to such $\alpha$. An example for such a scenario is the HB value $P_3(c^*=0.1, \alpha ^*=0.4325)$ in Example 4.2(ii) (not shown in Fig. 15); the movement of $\lambda _{\mathrm{M}}(P_3)$ is shown in Fig. 17. If $\lambda _{\mathrm{M}}(P_j)$ lies above $\lambda _{\mathrm{Ave}}(P_0)$, i.e., $\omega _c^*> \omega _{\mathrm{Ave}}^*$, we can still compute $\omega _c$ numerically for increasing values of $\alpha$, starting from $\alpha ^*$, and compare it with $\omega _{\mathrm{Ave}}$ as $\alpha$ passes over $\alpha _0$.

With parameter values in Example 4.2 and three different coupling strengths *c*, it is indicated in Fig. 18 that at larger coupling strength $c=0.1$, the collective frequency $\omega _c$ is always smaller than the average frequency $\omega _{\mathrm{Ave}}$, for $\alpha$ in a range where periodic solutions exist for both single-cell and coupled-cell HT model and PS model. In addition, the coupling strength *c* is smaller in the PS model than in the HT model, at which the collective frequency stays close to the average frequency in a range of $\alpha$.

In the following examples, we fix $\alpha$ and allow coupling strength *c* to increase. If it happens that $\omega _c$ is close to $\omega _{\mathrm{Ave}}$, we are interested to see whether it holds only for certain parameter values and the coupling strength. If it holds only for one of the HT model and PS model, we like to see the scenario for the other model.

#### Example 4.3.1

For parameters of the individual cells, we take $n=11$, $k_\mathrm{H}=0.136$ as in Examples 3.1, 4.1.1(i), 4.2(i) (HT model), and $A=0.0659$, $k_d=0.00001$ as in Examples 3.2, 4.1.1(ii), 4.2(ii) (PS model). In addition, we take the same time scaling factor $s=20$ and $\sigma =1.05$ as in the above examples. Recall that Hopf bifurcation occurs at $\alpha = \alpha _0 \approx 0.545174$ in Example 3.1 (single-cell HT model), and at $\alpha = \alpha _0 \approx 0.533809$ in Example 3.2 (single-cell PS model). We fix $\alpha =0.60139$ so that the single-cell HT model and the single-cell PS model have analogous oscillatory wave forms, as in Fig. 4.

**Fig. 19:**
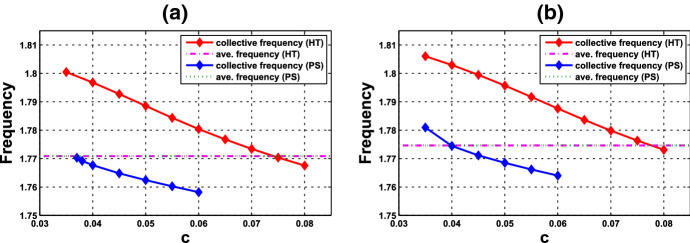
Relation between the collective frequency and the average of individual frequencies for a range of coupling strength *c*, when **a** $\alpha =0.60139$, the average frequency is 1.77086089 for $f=f_1$, and 1.77086095 for $f=f_2$, **b** $\alpha = 0.56$, the average frequency is 1.774609880 for $f=f_1$, and 1.774625695 for $f=f_2$

Via solving the ODE system (39) numerically, we find the phase-locked periodic solutions for *c* starting from $c = 0.035$ in the coupled-cell HT model and from $c = 0.037$ in the coupled-cell PS model. Then, we observe the relation between the collective frequencies and the average of the individual frequencies. The results are shown in Fig. 19a. At the coupling strength $c\approx 0.037$, the collective frequency $\omega _c$ is less than and close to the average frequency $\omega _{\mathrm{Ave}}$ in the coupled-cell PS model, whereas the difference between $\omega _c$ and $\omega _{\mathrm{Ave}}$ is substantial in the coupled-cell HT model. If we further increase the coupling strength *c* in the coupled-cell HT model, the collective frequency tends to the average frequency when $c\approx 0.075$.

In Example 4.3.1, $\alpha =0.60139$ may be too far from the values of $\alpha ^*$ in Examples 4.2.1, 4.2.2. Let us consider the value of $\alpha$ which is closer to $\alpha ^*$.

#### Example 4.3.2

Consider the same parameter values in Example 4.3.1, except that here we choose $\alpha =0.56$ in both models (larger than $\alpha _0$ and all $\alpha ^*$ in Example 4.2) and vary the coupling strength *c*. We compute numerically and observe the phase-locked periodic solutions starting from $c = 0.035$ in both models. The results are shown in Fig. 19b. At $c = 0.035$, the collective frequency is greater than the average frequency in both models. For the coupled-cell PS model, the collective frequency matches the average frequency at coupling strength $c \approx 0.04$. At this value of *c*, for the coupled-cell HT model, the collective frequency is above the average frequency. If we still increase the coupling strength in the coupled-cell HT model, the collective frequency matches the average frequency when $c\approx 0.0776$.

The above examples indicate that starting with similar oscillatory wave forms for individual cells in the HT model and in the PS model, the collective frequency of the coupled-cell system decreases as the coupling strength increases in both models. Moreover, the collective frequency drops faster in the PS model than in the HT model, as the coupling strength increases. In both models, there exist coupling strengths *c* such that the collective frequency equals the average frequency of individual cells. For the HT model, such strength *c* is larger. Our result in this regard is basically consistent with the one reported in Kim et al. (2014). It was reported in Kim et al. (2014) that protein-sequestration-based repression is more suitable for modeling circadian rhythms of mammals. One of the reasons is that the average frequency property holds for the PS model at suitable coupling strength, whereas this property holds for the HT model at coupling strength unreasonably large. Herein, we have proposed a methodology to examine closely the parameter values at which the average frequency property holds. Regarding which repression mechanism is more suitable for the modeling, among other concerns such as robustness, pertinence of parameter values in the models is certainly crucial.

## Segmentation Clock Model

It is interesting to see the scenario of collective frequencies for other biological oscillators and compare it with the ones in Sect. 4. In this section, we perform parallel analysis and computation to a mathematical model on segmentation clock genes in zebrafish (Chen et al. 2018; Herrgen et al. 2012; Uriu et al. 2010). There are delay models and ODE models depicting the somitogenesis of zebrafish. We consider the following ODE system:

44 $$\begin{aligned} \left\{ \begin{aligned} \displaystyle {\dot{x}}_1&= \displaystyle \frac{k_1^n}{k_1^n + x_3^n}(\nu _1 + \nu _c y_4) - \frac{\nu _2 x_1}{k_2 +x_1} \\ \displaystyle {\dot{x}}_2&= \displaystyle \nu _3 x_1 -\frac{\nu _4 x_2}{k_4 +x_2} -\nu _5 x_2 \\ \displaystyle {\dot{x}}_3&= \displaystyle \nu _5 x_2 -\frac{\nu _6 x_3}{k_6 +x_3} \\ \displaystyle {\dot{x}}_4&= \displaystyle \frac{\nu _7 k_7^h}{k_7^h + x_3^h} - \frac{\nu _8 x_4}{k_8 +x_4} \\ \displaystyle {\dot{y}}_1&= \displaystyle \sigma \left[ \frac{k_1^n}{k_1^n + y_3^n}\left( \nu _1 + \frac{\nu _c}{\sigma } x_4 \right) - \frac{\nu _2 y_1}{k_2 +y_1}\right] \\ \displaystyle {\dot{y}}_2&= \displaystyle \sigma \left[ \nu _3 y_1 -\frac{\nu _4 y_2}{k_4 +y_2} -\nu _5 y_2 \right] \\ \displaystyle {\dot{y}}_3&= \displaystyle \sigma \left[ \nu _5 y_2 -\frac{\nu _6 y_3}{k_6 +y_3}\right] \\ \displaystyle {\dot{y}}_4&= \displaystyle \sigma \left[ \frac{\nu _7 k_7^h}{k_7^h + y_3^h} - \frac{\nu _8 y_4}{k_8 +y_4}\right] . \\ \end{aligned} \right. \end{aligned}$$

System (44) depicts the interaction of two nonidentical cells if $\sigma \ne 1$, and two identical cells if $\sigma = 1$. Synchronous oscillations and traveling waves for the *N*-cell system, expanded from system (44) with $\sigma =1$, were studied in Chen et al. (2018), Liao and Shih (2012), Liao et al. (2012), Uriu et al. (2009, 2010). All parameters are positive, and their meanings can be found in those papers. In particular, Hill coefficients *n* and *h* are positive integers. When the coupling strength $\nu _c = 0$, system (44) reduces to two decoupled subsystems, each of four dimension. We arrange the discussion of periodic solutions of single-cell systems in Supplementary Materials II.

The Hopf bifurcation theory has been applied to investigate synchronous oscillations in system (44) with $\sigma =1$, and in delay models in Chen et al. (2018), Liao and Shih (2012). It was shown that the collective frequency decreases as the coupling strength $\nu _c$ increases. Herein, we investigate the periodic solution of system (44) which comprises two nonidentical subsystems, i.e., when $\sigma \ne 1$.

The existence of positive equilibrium $\overline{{\mathbb {X}}}=(\bar{{\mathbf{x}}},\bar{{\mathbf{y}}})= ({\bar{x}}_1,{\bar{x}}_2,{\bar{x}}_3,{\bar{x}}_4,{\bar{y}}_1,{\bar{y}}_2,{\bar{y}}_3,{\bar{y}}_4)$ for system (44) can be argued by the implicit function theorem, and is similar to the one in Appendix E. The Jacobian matrix associated with the linearization of system (44) at $\overline{{\mathbb {X}}}$ can be computed, as shown in Supplementary Materials II.

The characteristic polynomial $\varDelta (\lambda ):=\mathrm{det}(\lambda I-J(\overline{{\mathbb {X}}};\nu _1))$ is

45 $$\begin{aligned} \lambda ^8+b_1\lambda ^7+b_2\lambda ^6+b_3\lambda ^5+b_4\lambda ^4 +b_5\lambda ^3+b_6\lambda ^2+b_7\lambda +b_8, \end{aligned}$$

where the coefficients $b_i$ can be computed. To apply the Hopf bifurcation theorem to system (44), we look for the value of $\nu _1=\nu _1^*$ and the values for the other parameters, which satisfy the conditions of Theorem 1 with $m=8$. We follow the computation steps in Sect. 4.2.

We are interested in seeing how the collective frequency $\omega _c$ of oscillation in system (44) varies with the coupling strength $\nu _c$ and parameter $\nu _1$. We adopt the parameter values in Example S for single cell (in Supplementary Material II), where $\nu _1^\diamond$ denotes the HB value, and the frequency for the isolated ($\nu _c=0$) individual cell is approximately $\omega _0$ in Theorem 5 (in Supplementary Material II). The HB values are denoted by $(\nu _c^*, \nu _1^*)$.

### Example 5.1

**Fig. 20:**
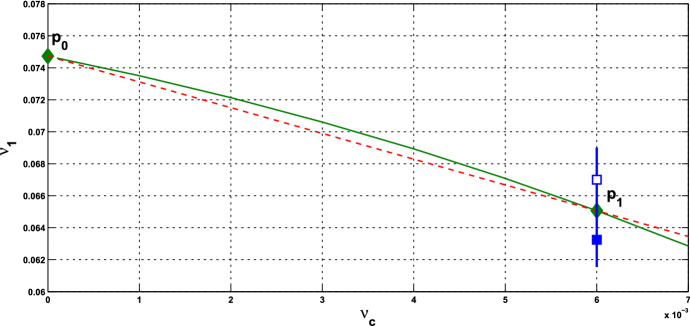
For Example 5.1: The HB curve plotted by green curve on $(\nu _c,\nu _1)$-plane; $P_0$ is a HB value for the single-cell system, $P_1$ is a HB value for the coupled-cell system, the segment $P_0P_1$ in red line, the HB course in blue line (Color figure online)

**Fig. 21:**
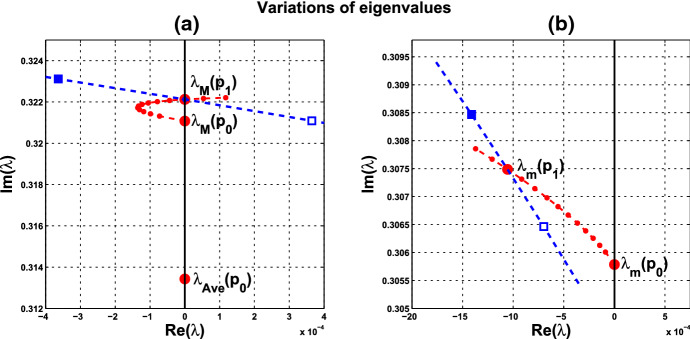
Variations of two complex eigenvalues as $(\nu _c,\nu _1)$ moves along segment $P_0P_1$ (red), and the HB course (blue) in Fig. 20: **a** $\lambda _{\mathrm{M}}$-branch reaches the imaginary axis at $P_1(0.006,0.06507)$; $\lambda _{\mathrm{M}}$-branch along the course, from solid square to hollow square, crosses the imaginary axis at parameter value $P_1$. **b** $\lambda _{\mathrm{m}}$-branches enter into and stay in the left complex plane along $P_0P_1$ and the course. A supercritical Hopf bifurcation occurs at $\nu _1^*\approx 0.06507$. $\lambda _{\mathrm{Ave}}(P_0):=[\lambda _{\mathrm{m}}(P_0)+\lambda _{\mathrm{M}}(P_0)]/2$ (Color figure online)

Consider coupled system (44) with parameter values in Example S and $\sigma =1.05$. When $\nu _c=0$, the system is decoupled, and each of the two subsystems has a periodic solution with respective frequency about $\omega _1^*=0.305785$ and $\omega _2^*=1.05 \cdot \omega _1^*$. The HB curve consisting of the HB values $(\nu _c^*,\nu _1^*)$ is drawn in Fig. 20. For illustration, we take the line segment $P_0P_1$, with $P_0(\nu _c,\nu _1^\diamond )$, $P_1(\nu _c^*,\nu _1^*)$, where $(\nu _c, \nu _1^\diamond )=(0,0.074725)$ and $(\nu _c^*,\nu _1^*)=(0.006,0.06507)$. The variation of eigenvalues along $P_0P_1$ is depicted in Fig. 21a, c. There are two branches of complex-conjugate eigenvalues $\lambda _{\mathrm{M}}, {\overline{\lambda }}_{\mathrm{M}}$ and $\lambda _{\mathrm{m}}, {\overline{\lambda }}_{\mathrm{m}}$, and four negative real eigenvalues. We denote by $\lambda _{\mathrm{M}}(P)$ and $\lambda _{\mathrm{m}}(P)$ the eigenvalues of $\lambda _{\mathrm{M}}$ and $\lambda _{\mathrm{m}}$ at point *P*. When $\nu _c=0$, $\lambda _{\mathrm{M}}(P_0)$ and $\lambda _{\mathrm{m}}(P_0)$ are purely imaginary, with $\lambda _{\mathrm{M}}(P_0)=i \omega _2^*=1.05 \cdot \lambda _{\mathrm{m}}(P_0)= 1.05 \cdot i\omega _1^*$. As the parameters $\nu _c$ and $\nu _1$ vary along $P_0P_1$, $\lambda _{\mathrm{M}}$-branch moves to the left complex plane, and makes a turn upward to reach the imaginary axis at $P_1$. That is, $\lambda _{\mathrm{M}}(P_1)$ lies above $\lambda _{\mathrm{M}}(P_0)$. On the other hand, along the route, $\lambda _{\mathrm{m}}$-branch moves to and stays in the left complex plane. To summarize, at $P_1$, there is a pair of purely imaginary eigenvalues $\lambda _{\mathrm{M}}(P_1), {\overline{\lambda }}_{\mathrm{M}}(P_1)$ and the remaining eigenvalues have negative real parts, including $\lambda _{\mathrm{m}}(P_1)$. These eigenvalues are $\pm 0.322124i$, $-0.001056\pm 0.307488 i$, $-0.338223$, $-0.362978$, $-0.712781$, and $-0.749678$.

Variation of eigenvalues along the HB course is depicted in Fig. 21a, b. We observe from numerical computations that periodic solutions emerge at $\nu _1 > \nu _1^*$ for each $\nu _1^*$, and $\nu _1$ close to $\nu _1^*$, with frequency about $\omega _c^* \approx 0.322124$. The scenarios at other Hopf bifurcation points $(\nu _c^*, \nu _1^*)$ in Fig. 20 are similar to the ones for $P_1$ in Fig. 21.

**Fig. 22:**
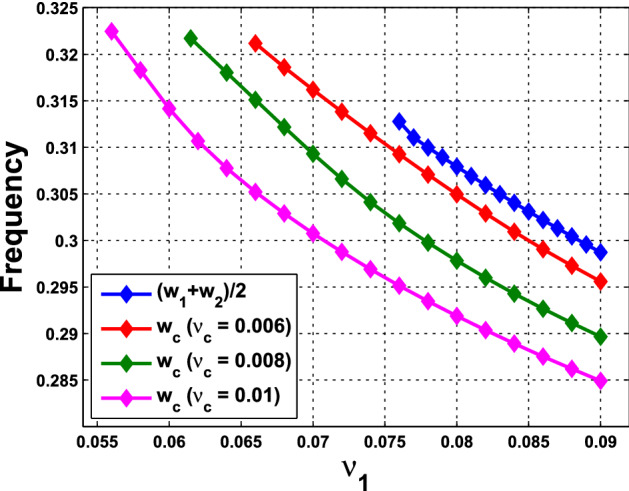
Average frequency $\omega _{\mathrm{Ave}}=(\omega _1+\omega _2)/2$ with respect to $\nu _1$ for $\nu _1>\nu _1^\diamond$, at $\nu _c=0$, and collective frequency $\omega _c$ with respect to $\nu _1$ for $\nu _1>\nu _1^*$, at $\nu _c=0.006, 0.008, 0.01$, respectively

For $\nu _c=0.006, 0.008, 0.01$, respectively, we increase $\nu _1$ further over $\nu _1^*$, while holding $\nu _c$ fixed at each $\nu _c^*$, and perform numerical computation on system (44) to observe the periodic solutions and see how their frequencies vary with $\nu _1$. The result is shown in Fig. 22. The leftmost points of the plots represent the collective frequencies which are approximately $\omega _c^*$, corresponding to $\nu _1$ slightly larger than $\nu _1^*$, respectively, and the average frequency about $\omega ^*_{\mathrm{Ave}}=(\omega _1^*+\omega _2^*)/2 \approx (0.305785+1.05 \cdot 0.305785)/2$ corresponding to $\nu _1$ slightly larger than $\nu _1^\diamond$. It can be seen that, in each case, both the collective frequency and the average frequency decrease as $\nu _1$ increases.

Next, let us see how coupling strength $\nu _c$ affects the collective frequency and compare it with the average of the individual frequencies.

### Example 5.2

**Fig. 23:**
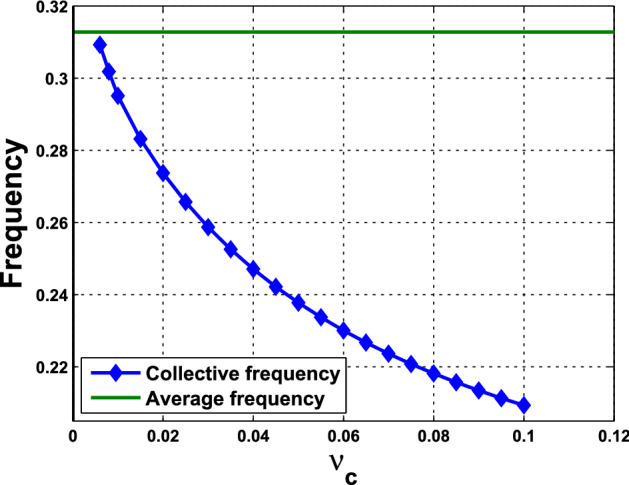
Collective frequency versus the average of individual frequencies, for increasing values of $\nu _c$, with $\nu _1 = 0.076$

With parameter values in Example S, single-cell systems (54) and (55) undergo a Hopf bifurcation at $\nu _1 = \nu _1^\diamond \approx 0.074725$, and coupled-cell system (44) with $\nu _c=0.006$ undergoes a Hopf bifurcation at $\nu _1 = \nu _1^{*}\approx 0.06507$. We take $\nu _1 = 0.076$ so that the stable periodic solutions exist in both coupled-cell system (44) and single-cell systems (54), (55). Next, we increase the coupling strength from $\nu _c=0.006$ and observe the relation between the collective frequency and the average frequency. The relations are depicted in Fig. 23. When $\nu _c = 0.1$, the collective frequency $\omega _c\approx 0.209346$, i.e., the collective period $T_c\approx 30.013387$, falls within the range [25,35] minutes of biological interest. We observe that as the coupling strength increases, the collective frequency decreases and deviate farther from the average frequency of individual cells. This can also be seen in Fig. 22. The scenario is different from the one in coupled-cell HT and PS models, discussed in Sect. 4.

We remark that for some other parameter values, the periodic solutions may not persist for $\nu _1$ extending well over $\nu _1^*$ in system (44). From the theory, the Hopf bifurcation occurs, and hence, the stable periodic solutions are confirmed to exist, at $\nu _1$ slightly over $\nu _1^*$ in coupled-cell system (44), and $\nu _1$ slightly over $\nu _1^\diamond$ in single-cell systems (54) and (55). In our computations, the values of $\nu _1^*$ are all smaller than those of $\nu _1^\diamond$. If the periodic solution of system (44) does not exist for $\nu _1$ at least larger than $\nu _1^\diamond$, then we will not be able to compare the collective frequency and the average frequency at the same parameter values. Nevertheless, the present approach still leads us to locate the parameter values at which the existence of periodic solutions for the single-cell systems and the ones for the coupled-cell systems is assured.

## Discussions and Conclusions

We have investigated the stable periodic solutions and their properties in some single-cell systems and coupled-cell systems. The methodology is based on the Hopf bifurcation theory and numerical simulations on the considered systems at parameter values unfolding from where the bifurcations occur. In addition, vanishing determinant for one of the Hurwitz matrices in the Routh–Hurwitz criterion leads to a condition which determines the parameter values where the Hopf bifurcation takes place. The condition not only detects and confirms the occurrence of Hopf bifurcation, but also allows us to analyze the bifurcation. With this approach, we further explored how the frequency of oscillation in the coupled-cell systems varies with respect to a system parameter and the coupling strength.

We applied this approach to investigate oscillations, represented by stable periodic solutions, and their frequencies, in a system modeling minimal genetic negative feedback loop. We analyzed the oscillatory properties and compared these properties between Hill-type repression and protein-sequestration-based repression. Taking $\alpha$ as the bifurcation parameter, we computed the bifurcation value $\alpha _0$ for the single-cell systems. Then, we located the parameter values at which the oscillatory wave forms for the two systems, each with one of these two repressions, resemble each other. Then, for the coupled system comprising such single cells, we investigated how (at what parameter values) the collective periodic solutions emerge. We computed the eigenvalues of the linearized system along parameters $(c, \alpha )$ on the line segment from $(0, \alpha _0)$ to $(c^*, \alpha ^*)$, where $\alpha ^*$ is a HB value of the coupled-cell system at coupling strength $c=c^*$. The purely imaginary eigenvalues provide the magnitudes of the collective frequencies at $\alpha =\alpha ^*$. This exhibits the relative magnitude between the average frequency of individual cells at $\alpha$ near $\alpha _0$ and the collective frequency of coupled-cell system at $\alpha$ near $\alpha ^*$. Unfolding from such information, we further computed to observe how the collective frequency of oscillation varies with the coupling strength and $\alpha$. Moreover, we extended the computation to compare the average frequency with the collective frequency at the same value of $\alpha$, and discussed the average frequency property. The collective behaviors for the coupled systems with two types of repression were compared. We observed that the collective frequency in the coupled-cell system with protein-sequestration-based repression approaches the average frequency at smaller coupling strength than the one with Hill-type repression.

For the system comprising two identical cells, we were able to express explicitly the HB values in terms of the component of the equilibrium, and the collective frequency at the HB values. This expression reveals the role played by the repression function on the frequency property. The influence from the Hill-type repression or from the protein-sequestration-based repression is indicated in the expression by the slope of the repression function. It can also be seen that the effect in the collective frequency at the HB values from this slope is enhanced by large coupling strength and suppressed by large timescale of the intercellular coupling. For further oscillatory properties at parameter values beyond the HB values, we resorted to numerical computations on the ODE systems. For the system comprising two nonidentical cells, an eight-dimensional ODE system, we still used the criterion in Liu (1994) to detect the Hopf bifurcation, but this process was carried out through numerical computations, as described in Sect. 4.2.

To compare with other biological oscillators, we performed a similar analysis and computation to a segmentation clock model. We observed different variations of eigenvalues along the parameter values connecting the HB value of the single-cell system and the HB value of the coupled-cell system. It appears in all our computations that the collective frequency of such coupled-cell system does not match the average frequency of individual cells.

Another significant issue is that our results enable a comparison between the oscillatory properties indicated in the kinetic models which take into account gene regulations and the ones obtained from the phase equations. As mentioned in Introduction, the average frequency property in the coupled phase Eq. (3) holds under the assumption of odd interaction function and symmetric connection. In our discussions in Sects. 4 and 5, the average frequency property only holds for specific parameter values or does not hold, even for small coupling strengths, see Figs. 18 and 22. Therefore, whether and under what assumption can the coupled phase equations accommodate the oscillatory properties of the coupled-cell systems remains an issue to be further examined.

In a sense of coordinating two individual oscillators with different frequencies, it was conceived that the coupling strength *c* has to attain a threshold (coupling strength is strong enough) to generate a collective periodic solution and the cells oscillate at a compromise frequency. The notion of compromise frequency was discussed in a coupled phase equation in Strogatz (1994). It was shown therein that the phase-locked solution exists only if the total coupling strength is larger than the difference of the individual frequencies, in a two-component phase equation. On the contrary, in Sect. 4.2, we saw that the HB curves in Figs. 12 and 15 can extend to small values of coupling strength *c* (as small as $10^{-8}$ in AUTO computation). Our numerical computation shows that a supercritical Hopf bifurcation still occurs for $c=10^{-4}$, but the bifurcating periodic solution has very small amplitude. The difference of individual frequencies for these two decoupled cells is about $0.05 \cdot \sqrt{3}$ which is much larger than the coupling strength.

It has been our goal to develop an efficacious mathematical and computational approach to tackle the problems about how the oscillations of the individual cells with different intrinsic frequencies compromise to generate a collective periodic solution through intercellular coupling, and how the collective frequency of oscillation changes with the coupling strength and other parameters. The Hopf bifurcation theory provides a theoretical basis for confirming the existence of stable periodic solutions so that the findings on oscillations, their frequencies, and relevant dynamics are not merely based on numerical simulations. Combining effective numerical computation with the Hopf bifurcation theory, as demonstrated in this paper, one expands the capacity to observe the correspondence between oscillations and parameter values. Some of the examples in this work are based on the HB theory, whereas the other examples are extended from the HB theory via numerical simulations. Although the later are phenomenological findings, the present approach provides a way to explore the nature of single-cell systems and coupled-cell systems and make a close observation and comparison on the kinetics induced by Hill-type repression and the ones by protein-sequestration-based repression. The kinetics in terms of the properties imbedded in these mathematical models on biological processes can then be understood more thoroughly. We hope that the present approach has also contributed toward the selection or tuning of parameter values in the modeling.

## Figures and Tables

**Fig. 24:**
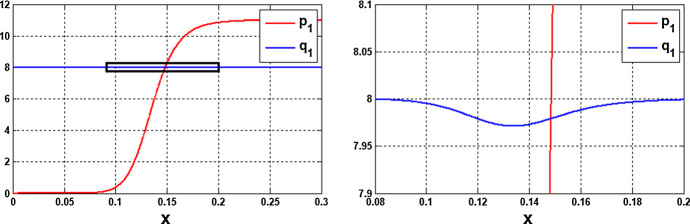
The intersection for the graphs of $p_1(x)$ and $q_1(x)$, with parameter values in Example 4.1.1(i): $n=11$, $k_\mathrm{H}=0.136$, $s=20$, and $c=0.01$. The right figure zooms in the left one

**Fig. 25:**
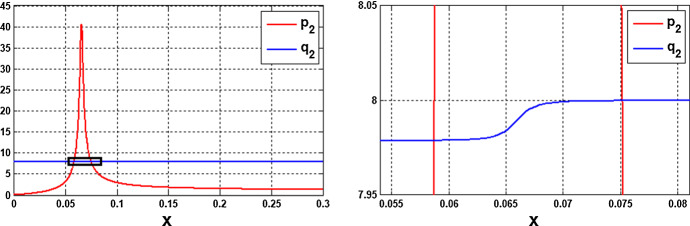
The intersection for the graphs of $p_2(x)$ and $q_2(x)$, with parameter values in Example 4.1.1(ii): $A=0.0659$, $k_d=0.00001$, $s=20$, and $c=0.01$. The right figure zooms in the left one

**Fig. 26:**
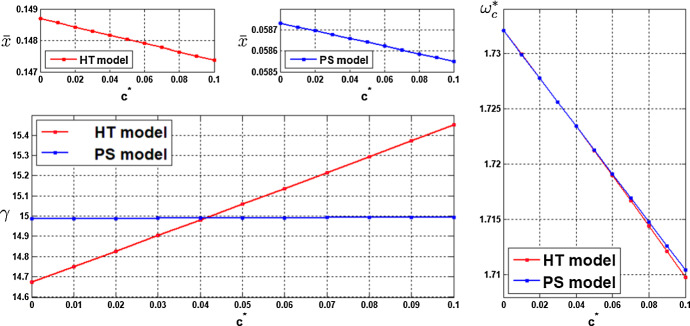
The values for the component ${\bar{x}}$ of the equilibrium, $\gamma$ and $\omega _c^*$ at the HB values $(c^*, \alpha ^*)$ along the HB curve. Here, $n = 11$, $k_\mathrm{H} = 0.136$, $A = 0.0659$, $k_d = 0.00001$, and $s =20$

**Fig. 27:**
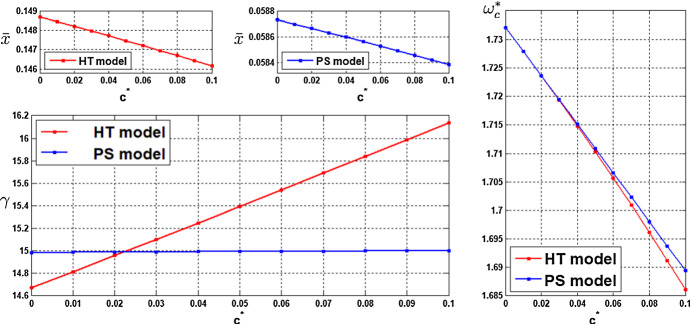
The values for the component ${\bar{x}}$ of the equilibrium, $\gamma$ and $\omega _c^*$ at the HB values $(c^*, \alpha ^*)$ along the HB curve. Here, $n = 11$, $k_\mathrm{H} = 0.136$, $A = 0.0659$, $k_d = 0.00001$, and $s =10$

**Fig. 28:**
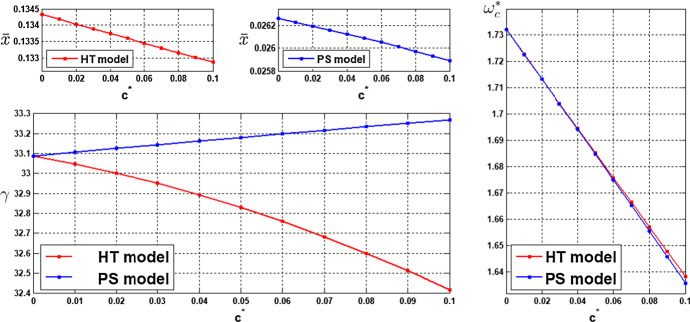
The values for the component ${\bar{x}}$ of the equilibrium, $\gamma$ and $\omega _c^*$ at the HB values $(c^*, \alpha ^*)$ along the HB curve. Here, $n = 18$, $k_\mathrm{H} = 0.136$, $A = 0.029375$, $k_d = 0.00001$, and $s=10$

**Fig. 29:**
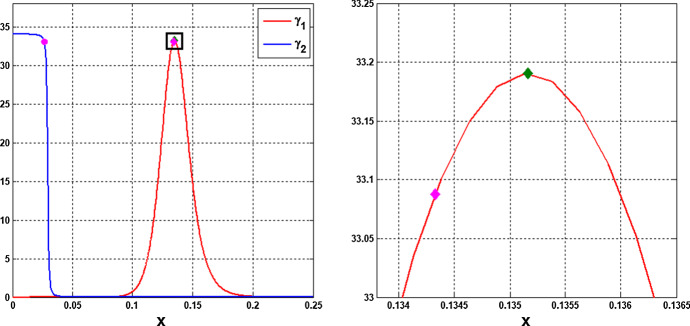
The graphs of $\gamma _1(x)$ and $\gamma _2(x)$, with $n = 18$, $k_\mathrm{H} = 0.136$, $A = 0.029375$, $k_d = 0.00001$. The green diamond represents $x_M \approx 0.135162$, the magenta diamond represents ${\bar{x}}\approx 0.134324$ when $c=0$ in the HT model, and the magenta circle represents ${\bar{x}}\approx 0.025891$ when $c = 0$ in the PS model. The right figure zooms in the left one (Color figure online)

**Fig. 30:**
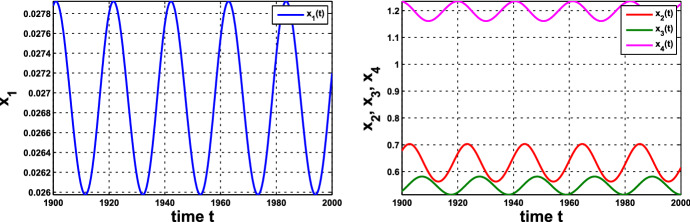
The solution $(x_1(t), x_2(t), x_3(t), x_4(t))$ of system (54) with $\nu _1=0.076$, evolved from (0.02653,  0.5969,  0.4705,  1.3115), approaches a periodic solution with period about 20.591043

## Appendices

**A.** Crossing condition for single-cell system: We consider

46 $$\begin{aligned} \frac{\mathrm{d}}{\mathrm{d}\alpha }[b_1(\alpha )b_2(\alpha )-b_3(\alpha )]\vert _{\alpha =\alpha _0}= & {} -\frac{8\gamma '(\alpha _0)}{\gamma (\alpha _0)}-\gamma (\alpha _0) \nonumber \\= & {} -\frac{1}{\gamma (\alpha _0)}[8\gamma '(\alpha _0)+\gamma ^2(\alpha _0)], \end{aligned}$$

where

$$\begin{aligned} \gamma (\alpha ):=-f'({\bar{x}}(\alpha )),~\gamma '(\alpha )= -f''({\bar{x}}(\alpha )){\bar{x}}'(\alpha ) = -\frac{f''({\bar{x}}(\alpha )) f^2({\bar{x}}(\alpha ))}{f({\bar{x}}(\alpha ))+{\bar{x}}(\alpha )\gamma (\alpha )}, \end{aligned}$$

with

$$\begin{aligned} {\bar{x}}'(\alpha )=\frac{1}{\alpha '({\bar{x}})}=\left[ \frac{\mathrm{d}}{\mathrm{d}{\bar{x}}} \left( \frac{{\bar{x}}}{f({\bar{x}})}\right) \right] ^{-1}= \frac{f^2({\bar{x}})}{f({\bar{x}})-{\bar{x}}f'({\bar{x}})}. \end{aligned}$$

For $f=f_1$, $\gamma =\gamma _1$, and at $\alpha =\alpha _0=8/\gamma _1$, we compute from $\alpha f_1({\bar{x}})={\bar{x}}$ to find ${\bar{x}} = k_\mathrm{H} (8/(n-8))^{1/n}, ~n>8$. Thus,

$$\begin{aligned} \gamma _1(\alpha _0)=-f_1'({\bar{x}}(\alpha _0))= \frac{k_\mathrm{H}^n n {\bar{x}}^{n-1}(\alpha _0)}{[k_\mathrm{H}^n+{\bar{x}}^n(\alpha _0)]^2}= \frac{8^{\frac{n-1}{n}}(n-8)^{\frac{n+1}{n}}}{nk_\mathrm{H}}, \end{aligned}$$

and

$$\begin{aligned} \gamma _1'(\alpha _0)=-f_1''({\bar{x}}(\alpha _0))\cdot \frac{\mathrm{d}{\bar{x}}}{\mathrm{d}\alpha }\vert _{\alpha =\alpha _0} =\frac{8^{\frac{n-2}{n}}(n-8)^\frac{2n+2}{n}(n^2-17n)}{9n^3k_\mathrm{H}^2}, \end{aligned}$$

with

$$\begin{aligned} -f_1''({\bar{x}}(\alpha _0))=\frac{8^{\frac{n-2}{n}}(n-8) ^\frac{n+2}{n}(n^2-17n)}{n^2k_\mathrm{H}^2}, \end{aligned}$$

and

$$\begin{aligned} \frac{\mathrm{d}{\bar{x}}}{\mathrm{d}\alpha }\vert _{\alpha =\alpha _0}=\frac{n-8}{9n}. \end{aligned}$$

Therefore, (46) becomes

$$\begin{aligned} -\frac{1}{\gamma _1(\alpha _0)}[8\gamma _1'(\alpha _0)+\gamma _1^2(\alpha _0)] = -\frac{8^{\frac{n-1}{n}}(n-8)^\frac{2n+1}{n}}{9nk_\mathrm{H}}, \end{aligned}$$

which is nonzero for $n>8$.

For $f=f_2$, $\gamma =\gamma _2$, and at $\alpha =\alpha _0=8/\gamma _2$, we compute from $\alpha f_2({\bar{x}})={\bar{x}}$ to find

$$\begin{aligned} {\bar{x}} = \frac{64}{63}(A-k_d)\pm \frac{8}{63}\sqrt{(A-k_d)^2-252 A k_d}. \end{aligned}$$

We assume (25) and take ${\bar{x}}$ in (26). Thus, at $\alpha _0=8/\gamma _2=-8/f'_2({\bar{x}})$, $\gamma _2(\alpha _0)=-N_0/D_0$, where

$$\begin{aligned} N_0&:=A-k_d-8\sqrt{(A-k_d)^2-252Ak_d}\\&\quad -\left[ 65(A-k_d)^2-252Ak_d-16(A-k_d) \sqrt{(A-k_d)^2-252Ak_d}\right] ^{\frac{1}{2}}\\ D_0&:=2A\left[ 65(A-k_d)^2-252Ak_d-16(A-k_d)\sqrt{(A-k_d)^2-252Ak_d}\right] ^{\frac{1}{2}} \end{aligned}$$

and $$\gamma _2'(\alpha _0)=N_1/D_1$$, where

$$\begin{aligned} \begin{aligned} N_1&:= 3969k_d\left[ A-k_d-8\sqrt{(A-k_d)^2-252Ak_d}\right. \\&\quad \left. -[65(A-k_d)^2-252Ak_d-16(A-k_d)\sqrt{(A-k_d)^2-252Ak_d}]^{\frac{1}{2}}\right] \end{aligned} \end{aligned}$$

and

$$\begin{aligned} \begin{aligned} D_1&:= A[65(A-k_d)^2-252Ak_d-16(A-k_d)\sqrt{(A-k_d)^2-252Ak_d}]\\&\quad \times \left[ 64(A-k_d)-8\sqrt{(A-k_d)^2-252Ak_d}\right. \\&\quad \left. +[65(A-k_d)^2-252Ak_d -16(A-k_d)\sqrt{(A-k_d)^2-252Ak_d}]^{\frac{1}{2}}\right] . \end{aligned} \end{aligned}$$

Then, (46) becomes

$$\begin{aligned} -\frac{1}{\gamma _2(\alpha _0)}[8\gamma _2'(\alpha _0)+\gamma _2^2(\alpha _0)] = \frac{N}{D}, \end{aligned}$$

where

$$\begin{aligned} \begin{aligned} N&= 63\left[ A^2+1762Ak_d+k_d^2-8(A-k_d)\sqrt{(A-k_d)^2-252Ak_d} \right. \\&\quad \left. -(A-k_d)[65(A-k_d)^2-252Ak_d-16(A-k_d)\sqrt{(A-k_d)^2-252Ak_d}]^{\frac{1}{2}}\right] , \end{aligned} \end{aligned}$$

and

$$\begin{aligned} \begin{aligned} D&= 2A\left[ 65(A-k_d)^2-252Ak_d-16(A-k_d)\sqrt{(A-k_d)^2-252Ak_d}\right] ^{\frac{1}{2}} \\&\quad \times \left[ 64(A-k_d)-8\sqrt{(A-k_d)^2-252Ak_d}\right. \\&\quad \left. +[65(A-k_d)^2-252Ak_d -16(A-k_d)\sqrt{(A-k_d)^2-252Ak_d}]^{\frac{1}{2}}\right] . \end{aligned} \end{aligned}$$

Let us analyze the numerator *N*: Under $A>(127+48\sqrt{7})k_d$, we have

$$\begin{aligned}&(A-k_d)^2-252Ak_d>0 \\&65(A-k_d)^2-252Ak_d>0, ~16(A-k_d)\sqrt{(A-k_d)^2-252Ak_d}>0. \end{aligned}$$

Note that

$$\begin{aligned} {[}65(A-k_d)^2-252Ak_d]^2-[16(A-k_d)\sqrt{(A-k_d)^2-252Ak_d}]^2=3969(A-k_d)^4>0. \end{aligned}$$

With $A^2+1762Ak_d+k_d^2>0$, $8(A-k_d)\sqrt{(A-k_d)^2-252Ak_d}>0$, and $(A-k_d)[65(A-k_d)^2-252Ak_d-16(A-k_d)\sqrt{(A-k_d)^2-252Ak_d}]^{\frac{1}{2}}>0$, we have

$$\begin{aligned} \begin{aligned}&(A^2+1762Ak_d+k_d^2)^2-[8(A-k_d)\sqrt{(A-k_d)^2-252Ak_d}]^2 \\&\quad -[(A-k_d)[65(A-k_d)^2-252Ak_d-16(A-k_d)\sqrt{(A-k_d)^2-252Ak_d}]^{\frac{1}{2}}]^2 \\&\quad =4\sqrt{(A-k_d)^2-252Ak_d}[4(A-k_d)^3\\&\qquad -(32A^2+3023Ak_d+32k_d^2)\sqrt{(A-k_d)^2-252Ak_d}]. \end{aligned} \end{aligned}$$

There is only one intersection point $A=A^*$ for the graphs of functions $4(A-k_d)^3$ and $(32A^2+3023Ak_d+32k_d^2)\sqrt{(A-k_d)^2-252Ak_d}$. Thus, $N\ne 0$ if $A>(127+48\sqrt{7})k_d$ and $A\ne A^*$.

**B.** Equilibrium of system (30) with identical cells:

(I) Homogeneous equilibrium: A point $(x_1,x_2,x_3,x_4, y_1,y_2,y_3,y_4)$ is an equilibrium of system (30) if and only if

$$\begin{aligned}&x_1=x_2=x_3, f(x_3)=x_4, \alpha f(x_3) - x_1 + \frac{c}{2}[x_4 + y_4]=0,\\&y_1=y_2=y_3, f(y_3)=y_4, \alpha f(y_3) - y_1 + \frac{c}{2}[y_4 + x_4]=0. \end{aligned}$$

Suppose there exists an equilibrium. Combining these equalities yields

$$\begin{aligned} \alpha [f(x_1)-f(y_1)]=x_1-y_1. \end{aligned}$$

As $\alpha >0$, we have $x_1=y_1$ provided $f'<0$, by the mean value theorem. Subsequently, $x_i=y_i$, for $i=2, 3, 4$. That is, an equilibrium of system (30) has to be of the form

47 $$\begin{aligned} (\xi , \xi , \xi , f(\xi ), \xi , \xi , \xi , f(\xi )), \end{aligned}$$

where $\xi$ satisfies $(\alpha +c)f(\xi )=\xi$. With given $\alpha , c>0$, such $\xi >0$ always exists uniquely for $f=f_1, f_2$, as they are strictly decreasing, and $f_i(0)>0, \lim _{\xi \rightarrow \infty }f_i(\xi )=0$, $i=1,2$. Therefore, for any given parameters (with positive values), there always exists an unique positive equilibrium for system (30), and it has to be homogeneous (i.e., in the form (47)).

(II) Equilibrium of system (30) at the HB value: With $\alpha =\alpha ^*$ in (32), we solve $(\alpha +c)f({\bar{x}})={\bar{x}}$ to find positive ${\bar{x}}$. That is, we look for ${\bar{x}}$ which satisfies

48 $$\begin{aligned} \frac{\gamma {\bar{x}}}{f({{\bar{x}}})}=\frac{1}{2}[-(s-1)(s^2+4s+7) +(s+3)\sqrt{(s^2+3)^2-4sc\gamma }]+c\gamma , \end{aligned}$$

where $\gamma =-f'({\bar{x}})$. For $f=f_1$, $\gamma =\gamma _1 = (k_\mathrm{H}^n n{\bar{x}}^{n-1})/(k_\mathrm{H}^n+{\bar{x}}^n)^2$. For convenience, we drop the bar on *x*. We analyze the intersection of the graphs of $p_1(x)$ and $q_1(x)$, where

$$\begin{aligned} p_1(x)&:= \frac{nx^n}{k_\mathrm{H}^n+x^n} \\ q_1(x)&:= \frac{1}{2}[-(s-1)(s^2+4s+7)+(s+3)\sqrt{(s^2+3)^2-4sc\gamma _1}]+c\gamma _1. \end{aligned}$$

We compute to find

$$\begin{aligned} p_1'(x)=\frac{k_\mathrm{H}^n n^2 x^{n-1}}{(k_\mathrm{H}^n+x^n)^2},~\mathrm{and}~p_1''(x)= \frac{k_\mathrm{H}^n n^2 x^{n-2}[k_\mathrm{H}^n(n-1)-x^n(n+1)]}{(k_\mathrm{H}^n+x^n)^3}. \end{aligned}$$

Thus, $p_1(x)$ is increasing for $x>0$, concave upward for $0<x<x_{\mathrm{M}}$ and concave downward for $x>x_{\mathrm{M}}$, where $x_{\mathrm{M}}:=k_\mathrm{H}[(n-1)/(n+1)]^{1/n}$. In addition, $p_1(0)=0$ and $\lim _{x\rightarrow \infty } p_1(x)=n$. On the other hand, $q_1(0)=8$, $\lim _{x\rightarrow \infty } q_1(x)=8$, and $q_1'(x)=g_1(x)/h_1(x)$, where

$$\begin{aligned} g_1(x)&:=ck_\mathrm{H}^n nx^{n-2}[k_\mathrm{H}^n(n-1)-x^n(n+1)]\left\{ -s(s+3)\right. \\&\quad \left. +\left[ (s^2+3)^2 -\frac{4sck_\mathrm{H}^n nx^{n-1}}{(k_\mathrm{H}^n+x^n)^2}\right] ^{1/2}\right\} \\ h_1(x)&:=(k_\mathrm{H}^n+x^n)^3 \left[ (s^2+3)^2-\frac{4sck_\mathrm{H}^n nx^{n-1}}{(k_\mathrm{H}^n+x^n)^2}\right] ^{1/2}. \end{aligned}$$

It can be seen that there is only one critical point $x_{\mathrm{M}}$ for $q_1(x)$, when *c* is small. The existence of equilibrium at $\alpha =\alpha ^*$ thus follows. We draw the graphs of $p_1(x)$ and $q_1(x)$ for the parameter values in Example 4.1.1(i) in Fig. 24.

For $$f=f_2$$,

$$\begin{aligned} \gamma =\gamma _2 = \frac{A-{\bar{x}}-k_d+\sqrt{(A-{\bar{x}}-k_d)^2+4Ak_d}}{2A\sqrt{(A-{\bar{x}}-k_d)^2+4Ak_d}}. \end{aligned}$$

For convenience, we drop the bar on *x*. We look for the intersection of the graphs of $p_2(x)$ and $q_2(x)$, where

$$\begin{aligned} p_2(x)&:= \frac{x}{\sqrt{(A-x-k_d)^2+4Ak_d}}\\ q_2(x)&:= \frac{1}{2}[-(s-1)(s^2+4s+7)+(s+3)\sqrt{(s^2+3)^2-4sc\gamma _2}]+c\gamma _2. \end{aligned}$$

Note that $p_2(0)=0$, and $\lim _{x\rightarrow \infty } p_2(x)=1$. We compute to find

$$\begin{aligned} p_2'(x)= \frac{(A+k_d)^2-x(A-k_d)}{[(A-x-k_d)^2+4Ak_d]^\frac{3}{2}} \end{aligned}$$

and

$$\begin{aligned} p_2''(x)=\frac{(A-k_d)(A+k_d)^2-x[2(A^2+Ak_d+k_d^2)-x(A-k_d)]}{[(A-x-k_d)^2+4Ak_d]^\frac{5}{2}}. \end{aligned}$$

There is a critical point of $p_2(x)$ at $x=\frac{(A+k_d)^2}{A-k_d}$ and

$$\begin{aligned} p_2''\left( \frac{(A+k_d)^2}{A-k_d}\right) = -\frac{(A-k_d)^4}{8(A+k_d)^3(Ak_d)^\frac{3}{2}}<0. \end{aligned}$$

Thus, $p_2(x)$ attains a local maximum at this critical point, by the second derivative test. On the other hand, $\lim _{x\rightarrow \infty } q_2(x)=8$, and

$$\begin{aligned} q_2(0)= \frac{1}{2}\left[ -(s-1)(s^2+4s+7)+(s+3) \left[ (s^2+3)^2-\frac{4sc}{A+k_d}\right] ^{1/2}\right] +\frac{c}{A+k_d}. \end{aligned}$$

We compute to find $q_2'(x)=g_2(x)/h_2(x)$, where

$$\begin{aligned} g_2(x)= & {} 2ck_d \left[ s(s+3)- \left[ (s^2+3)^2-4sc\frac{A-x-k_d+ \sqrt{(A-x-k_d)^2+4Ak_d}}{2A\sqrt{(A-x-k_d)^2+4Ak_d}}\right] ^{1/2}\right] ,\\ h_2(x)= & {} [(A-x-k_d)^2+4Ak_d]^\frac{3}{2} \\&\left[ (s^2+3)^2-4sc \frac{A-x-k_d +\sqrt{(A-x-k_d)^2+4Ak_d}}{2A\sqrt{(A-x-k_d)^2+4Ak_d}}\right] ^{1/2}. \end{aligned}$$

At $$c=0$$, we have $$q_2(0)=8$$, $$g_2(x)>0$$ for $$s>1$$, and $$h_2(x)>0$$. Thus, we conclude that $$q_2(x) $$ is an increasing function with $$q_2(0)\approx 8$$, when *c* is small. Thus, there exist two equilibria at $$\alpha =\alpha ^*$$ when *c* is small. We draw the graphs of $$p_2(x)$$ and $$q_2(x)$$ for the parameter values in Example 4.1.1(ii) in Fig. 25.

**C.** The graphs of $\gamma _1$ and $\gamma _2$:

We compute to find $\gamma _1=\gamma _1({\bar{x}})= \frac{k_\mathrm{H}^n n {\bar{x}}^{n-1}}{(k_\mathrm{H}^n+{\bar{x}}^n)^2}$. We drop the bar on *x* and regard $\gamma _1$ as a function of *x*. Then,

$$\begin{aligned} \gamma _1'(x) = \frac{k_\mathrm{H}^n n x^{n-2}[k_\mathrm{H}^n(n-1)-x^n(n+1)]}{(k_\mathrm{H}^n+x^n)^3}. \end{aligned}$$

There is a critical point $x_{\mathrm{M}}:= k_\mathrm{H}\left( \frac{n-1}{n+1}\right) ^{\frac{1}{n}}$, where $\gamma _1$ attains a local maximum, by the first derivative test. In addition, $\gamma _1(0)=0$, and $\displaystyle \lim _{x\rightarrow \infty } \gamma _1(x)=0$.

We compute to find $\gamma _2=\gamma _2({\bar{x}})= \frac{A-{\bar{x}}-k_d+\sqrt{(A-{\bar{x}}-k_d)^2+4Ak_d}}{2A\sqrt{(A-{\bar{x}}-k_d)^2+4Ak_d}}$. Dropping the bar on *x*, then

$$\begin{aligned} \gamma _2'(x) = \displaystyle \frac{-2k_d}{[(A-x-k_d)^2+4Ak_d]^{\frac{3}{2}}}~\mathrm{and}~\gamma _2''(x)=\frac{-6k_d(A-x-k_d)}{[(A-x-k_d)^2+4Ak_d]^{\frac{5}{2}}}. \end{aligned}$$

Thus, $\gamma _2$ is decreasing for $x>0$, concave downward for $0<x<A-k_d$ and concave upward for $x>A-k_d$. In addition, $\gamma _2(0)=1/(A+k_d)$ and $\lim _{x\rightarrow \infty } \gamma _2(x)=0$. The graphs of $\gamma _1$ and $\gamma _2$ for the data in Example 4.1.1 are drawn in Fig. 10.

**D.** Numerics of ${\bar{x}}$, $\gamma _1$, $\gamma _2$, and $\omega _c^*$, along the HB curves:

(i) We take the parameter values in Example 4.1.1: $n=11$, $k_\mathrm{H}=0.136$, $A=0.0659$, and $k_d=0.00001$. We compute to find $x_{\mathrm{M}}=k_\mathrm{H} (\frac{n-1}{n+1})^{1/n}\approx 0.133764$, and ${\bar{x}} = k_\mathrm{H} (\frac{8}{n-8})^{1/n}\approx 0.148684$, where ${\bar{x}}$ is the component of the equilibrium for the single-cell HT model. Thus, ${\bar{x}}-x_{\mathrm{M}}\approx 0.014919$. On the other hand, ${\bar{x}}\approx 0.058730$ in the single-cell PS model. The component ${\bar{x}}$ of the equilibrium of coupled system (30) at $c=0.1$ is ${\bar{x}}\approx 0.147363$ in the HT model, and ${\bar{x}}\approx 0.058549$ in the PS model. The component ${\bar{x}}$ of the equilibrium of coupled system (30), the values of $\gamma _1$, $\gamma _2$, and the frequency $\omega _c^*$ at the HB values along the HB curve are plotted in Fig. 26.

(ii) Holding the parameter values in Example 4.1.1 and taking $s =10$ instead of $s=20$, we compute to find $x_M = k_\mathrm{H}(\frac{n-1}{n+1})^{1/n} \approx 0.133764$, and ${\bar{x}} = k_\mathrm{H}(\frac{8 }{n-8})^{1/n} \approx 0.148684$, where ${\bar{x}}$ is the component of the equilibrium for the HT single-cell model. Thus, ${\bar{x}}-x_M \approx 0.014919$. On the other hand, ${\bar{x}} \approx 0.059994$ in the single-cell PS model. The component ${\bar{x}}$ of the equilibrium of coupled system (30) at $c = 0.1$ is ${\bar{x}} \approx 0.146170$ in the HT model, and ${\bar{x}}\approx 0.058385$ in the PS model. The component ${\bar{x}}$ of the equilibrium of coupled system (30), the values of $\gamma _1$, $\gamma _2$, and the frequency $\omega _c^*$ at the HB values along the HB curve are plotted in Fig. 27. The difference between the model with $f_1$ and the model with $f_2$ is more obvious.

(iii) Taking $n = 18$, and parameter values $k_\mathrm{H} = 0.136$, $A = 0.029375$, $k_d = 0.00001$, and $s =10$, we compute to find $x_M = k_\mathrm{H}(\frac{n-1}{n+1})^{1/n} \approx 0.135162$, and ${\bar{x}} = k_\mathrm{H}(\frac{8 }{n-8})^{1/n} \approx 0.134324$, where ${\bar{x}}$ is the component of the equilibrium for the single-cell HT model. Thus, ${\bar{x}}-x_M \approx -0.000838$. On the other hand, ${\bar{x}} \approx 0.026266$ in the PS model. For the component ${\bar{x}}$ of the equilibrium of coupled system (30), at $c = 0.1$, ${\bar{x}} \approx 0.132864$ in the HT model, and ${\bar{x}}\approx 0.025891$ in the PS model. The component ${\bar{x}}$ of the equilibrium of coupled system (30), the values of $\gamma _1$, $\gamma _2$, and the frequency $\omega _c^*$ at the HB values along the HB curve are plotted in Fig. 28. The graphs of $\gamma _1$ and $\gamma _2$ are drawn in Fig. 29. Note that ${\bar{x}}$ now lies on the left hand side of $x_M$.

**E.** Existence of equilibrium for system (39) with nonidentical cells:

A point $(x_1,x_2,x_3,x_4, y_1,y_2,y_3,y_4)$ is an equilibrium of system (39) if and only if

$$\begin{aligned}&x_1=x_2=x_3, f(x_3)=x_4, \alpha f(x_3) - x_1 + \frac{c}{2}[x_4 + y_4]=0,\\&y_1=y_2=y_3, f(y_3)=y_4, \sigma [\alpha f(y_3) - y_1] + \frac{c}{2}[y_4 + x_4]=0. \end{aligned}$$

That is, an equilibrium of system (39) is in the form

$$\begin{aligned} (\xi , \xi , \xi , f(\xi ), \eta , \eta , \eta , f(\eta )), \end{aligned}$$

where $\xi , \eta$ satisfy $H_1(\xi , \eta , \sigma )=0, H_2(\xi , \eta , \sigma )=0$, with

$$\begin{aligned} H_1(\xi , \eta , \sigma )&:= \alpha f(\xi ) - \xi + \frac{c}{2}[f(\xi ) + f(\eta )],\\ H_2(\xi , \eta , \sigma )&:=\sigma [\alpha f(\eta ) - \eta ] + \frac{c}{2}[f(\eta ) + f(\xi )], \end{aligned}$$

regarding that all parameters other than $\sigma$ are fixed. Suppose $\xi _0$ satisfies $(\alpha +c)f(\xi _0)=\xi _0$. Then, $H_1(\xi _0, \xi _0, 1)=0, H_2(\xi _0, \xi _0, 1)=0$. We compute to have

$$\begin{aligned} \det \frac{\partial (H_1, H_2)}{\partial (\xi , \eta )}=[\alpha f'(\xi _0)-1][(\alpha +c) f'(\xi _0)-1] > 0 \end{aligned}$$

at $(\xi , \eta , \sigma )=(\xi _0, \xi _0, 1)$, due to $\alpha , c >0, f'<0$. Thus, there exists a unique branch of solutions $(\xi (\sigma ), \eta (\sigma ))$ so that $H_1(\xi (\sigma ), \eta (\sigma ), \sigma )=0, H_2(\xi (\sigma ), \eta (\sigma ), \sigma )=0$, with $(\xi (1), \eta (1))=(\xi _0, \xi _0)$, for $\sigma$ close to 1, according to the implicit function theorem. The existence of equilibrium of system (39) with $\sigma$ close to 1 is thus confirmed.

**Supplementary Materials**

**I.** The terms determining the properties of Hopf bifurcation in Theorem 2: Let $\alpha$ denote one of the parameters in system (7). While holding the other parameters at fixed values, we write the system as $d {\mathbf{x}}/\mathrm{d}t={\mathbf{f}}({\mathbf{x}}; \alpha )$. After translation to the equilibrium $\bar{{\mathbf{x}}}$, we put it into the form:

$$\begin{aligned} \dot{{\mathbf{x}}} = J(\bar{{\mathbf{x}}};\alpha ){{\mathbf{x}}} + \tilde{{\mathbf{f}}}({\mathbf{x}};\alpha ), \end{aligned}$$

where ${{\mathbf{x}}}=(x_1,x_2,\ldots ,x_m)$, $J(\bar{{\mathbf{x}}};\alpha ){{\mathbf{x}}}$ is the linear term, and $\tilde{{\mathbf{f}}}=(f_1,f_2,\ldots ,f_m)$ is the nonlinear term. At $\alpha =\alpha ^*$, $J(\bar{{\mathbf{x}}};\alpha ^*)$ has a pair of purely imaginary eigenvalues $\lambda (\alpha ^*)=\pm i\omega ^*$. Let us make a transformation so that the linear part is in normal form. We consider the change of variables ${{\mathbf{x}}}=P{{\mathbf{z}}}$, where

$$\begin{aligned} P= \left[ \begin{array}{ccccc} ~\mathrm{Im}({\mathbf{u}})&\mathrm{Re}({\mathbf{u}})&{\mathbf{v}}_3&\cdots&{\mathbf{v}}_m \end{array} \right] _{m \times m}, \end{aligned}$$

and ${\mathbf{u}}\in {\mathbb {R}}^m$ is the eigenvector of $J(\bar{{\mathbf{x}}};\alpha ^*)$ corresponding to the eigenvalue $i\omega ^*$ and ${\mathbf{v}}_3,\ldots ,{\mathbf{v}}_m$ are the real or imaginary parts of the generalized eigenvectors for the remaining eigenvalues. Thus,

49 $$\begin{aligned} P^{-1}J(\bar{{\mathbf{x}}};\alpha ^*)P = \left[ \begin{array}{ccc} ~0 &{} -\omega ^* &{} \vdots ~ \\ ~\omega ^* &{} 0 &{} \vdots ~ \\ ~\cdots &{} \cdots &{} D \end{array} \right] , \end{aligned}$$

where *D* is an $(m-2)\times (m-2)$ matrix. Then, the transformed system becomes

$$\begin{aligned} \dot{{\mathbf{z}}}=P^{-1}J(\bar{{\mathbf{x}}};\alpha )P{{\mathbf{z}}} + {\mathbf{F}}({{\mathbf{z}}};\alpha ), \end{aligned}$$

where ${{\mathbf{z}}}=(z_1,\ldots , z_m)$ and ${\mathbf{F}}({{\mathbf{z}}};\alpha )=P^{-1}{\tilde{\mathbf{f}}}(P{{\mathbf{z}}};\alpha )$ with ${\mathbf{F}}=(F_1,\ldots ,F_m)$. At $\alpha =\alpha ^*$, ${{\mathbf{z}}}={\mathbf{0}}$, we define

$$\begin{aligned} g_{11}&:= \frac{1}{4}\left[ \left( \frac{\partial ^2 F_1}{\partial z_1^2} +\frac{\partial ^2 F_1}{\partial z_2^2}\right) +i\left( \frac{\partial ^2 F_2}{\partial z_1^2}+\frac{\partial ^2 F_2}{\partial z_2^2}\right) \right] , \\ g_{02}&:= \frac{1}{4}\left[ \left( \frac{\partial ^2 F_1}{\partial z_1^2} -\frac{\partial ^2 F_1}{\partial z_2^2}-2\frac{\partial ^2 F_2}{\partial z_1 \partial z_2}\right) +i\left( \frac{\partial ^2 F_2}{\partial z_1^2}-\frac{\partial ^2 F_2}{\partial z_2^2}+2\frac{\partial ^2 F_1}{\partial z_1 \partial z_2}\right) \right] , \\ g_{20}&:= \frac{1}{4}\left[ \left( \frac{\partial ^2 F_1}{\partial z_1^2} -\frac{\partial ^2 F_1}{\partial z_2^2}+2\frac{\partial ^2 F_2}{\partial z_1 \partial z_2}\right) +i\left( \frac{\partial ^2 F_2}{\partial z_1^2}-\frac{\partial ^2 F_2}{\partial z_2^2}-2\frac{\partial ^2 F_1}{\partial z_1 \partial z_2}\right) \right] , \\ G_{21}&:= \frac{1}{8}\left[ \left( \frac{\partial ^3 F_1}{\partial z_1^3} +\frac{\partial ^3 F_1}{\partial z_1 \partial z_2^2}+\frac{\partial ^3 F_2}{\partial z_1^2 \partial z_2}+\frac{\partial ^3 F_2}{\partial z_2^3}\right) \right. \\&\quad \left. +i\left( \frac{\partial ^3 F_2}{\partial z_1^3}+\frac{\partial ^3 F_2}{\partial z_1 \partial z_2^2}-\frac{\partial ^3 F_1}{\partial z_1^2 \partial z_2}-\frac{\partial ^3 F_1}{\partial z_2^3}\right) \right] . \end{aligned}$$

Next, for $k=3,\ldots ,m$, we set

$$\begin{aligned} h^{(k-2)}_{11}&:=\frac{1}{4}\left( \frac{\partial ^2 F_k}{\partial z_1^2} + \frac{\partial ^2 F_k}{\partial z_2^2}\right) , \\ h^{(k-2)}_{20}&:=\frac{1}{4}\left[ \left( \frac{\partial ^2 F_k}{\partial z_1^2} - \frac{\partial ^2 F_k}{\partial z_2^2}\right) -2i\left( \frac{\partial ^2 F_k}{\partial z_1 \partial z_2}\right) \right] , \end{aligned}$$

evaluated at $\alpha =\alpha ^*$, ${{\mathbf{z}}}={\mathbf{0}}$. Let $w^{(k-2)}_{11}$, $w^{(k-2)}_{20} \in {{\mathbb {C}}}^{m-2}$ be the solutions of

$$\begin{aligned} Dw^{(k-2)}_{11}=-h^{(k-2)}_{11},~~(D-2i\omega _c^* I)w^{(k-2)}_{20}=-h^{(k-2)}_{20}, \end{aligned}$$

where *D* is defined in (49). For $k=3,\ldots , m$, let

$$\begin{aligned} G^{(k-2)}_{110}&:= \frac{1}{2}\left[ \left( \frac{\partial ^2 F_1}{\partial z_1 \partial z_k} + \frac{\partial ^2 F_2}{\partial z_2 \partial z_k}\right) +i\left( \frac{\partial ^2 F_2}{\partial z_1 \partial z_k} - \frac{\partial ^2 F_1}{\partial z_2 \partial z_k}\right) \right] , \\ G^{(k-2)}_{101}&:= \frac{1}{2}\left[ \left( \frac{\partial ^2 F_1}{\partial z_1 \partial z_k} - \frac{\partial ^2 F_2}{\partial z_2 \partial z_k}\right) +i\left( \frac{\partial ^2 F_2}{\partial z_1 \partial z_k} + \frac{\partial ^2 F_1}{\partial z_2 \partial z_k}\right) \right] , \end{aligned}$$

evaluated at $\alpha =\alpha ^*$, ${{\mathbf{z}}}={\mathbf{0}}$. Then we define

$$\begin{aligned} g_{21} := G_{21} + \sum ^{m-2}_{k=1} (2G^{(k)}_{110}w^{(k)}_{11} + G^{(k)}_{101}w^{(k)}_{20}). \end{aligned}$$

The following quantities can then be defined:

50 $$\begin{aligned} C_{1}(\alpha ^*)&:=\frac{i}{2\omega ^*}(g_{20}g_{11}-2|g_{11}|^{2}-\frac{1}{3}|g_{02}|^{2})+\frac{g_{21}}{2}, \end{aligned}$$

51 $$\begin{aligned} p_{2}&:=-\frac{\mathrm{Re}(C_{1}(\alpha ^*))}{\mathrm{Re}(\lambda '(\alpha ^*))}, \end{aligned}$$

52 $$\begin{aligned} \zeta _{2}&:=2\mathrm{Re}(C_{1}(\alpha ^*)),\end{aligned}$$

53 $$\begin{aligned} T_{2}&:=\frac{-1}{\omega ^*}[\mathrm{Im}(C_{1}(\alpha ^*))+p_{2}\mathrm{Im}(\lambda '(\alpha ^*))], \end{aligned}$$

where $\lambda (\alpha )$ is the branch of eigenvalue crossing the imaginary axis at $\alpha =\alpha ^*$, and $\lambda (\alpha ^*)=i \omega ^*$, with $\omega ^* >0$. The details can be found in Hassard et al. (1981).

The computed values of $C_1(\alpha _0)$, $p_2$, $\zeta _2$, and $T_2$ in Example 3.1 are

$$\begin{aligned} C_1(\alpha _0)= & {} -6.630408-13.971477i~\mathrm{and}~\lambda '(\alpha _0) = 0.611426+1.059021i,\\ p_2= & {} -\frac{\mathrm{Re}(C_1(\alpha _0))}{\mathrm{Re}(\lambda '(\alpha _0))} = 10.844170>0, \\ \zeta _2= & {} 2\mathrm{Re}(C_1(\alpha _0)) = -13.260817<0, \\ T_2= & {} -\frac{1}{\sqrt{3}}[\mathrm{Im}(C_1(\alpha _0))+p_2\mathrm{Im}(\lambda '(\alpha _0))] = 1.436028>0. \end{aligned}$$

The computed values of $C_1(\alpha _0)$, $p_2$, $\zeta _2$, and $T_2$ in Example 3.2 are

$$\begin{aligned} C_1(\alpha _0)= & {} -3.541903-6.152133i~\mathrm{and}~\lambda '(\alpha _0) = 0.624443 + 1.081567i,\\ p_2= & {} -\frac{\mathrm{Re}(C_1(\alpha _0))}{\mathrm{Re}(\lambda '(\alpha _0))} = 5.672099>0, \\ \zeta _2= & {} 2\mathrm{Re}(C_1(\alpha _0)) = -7.083806<0, \\ T_2= & {} -\frac{1}{\sqrt{3}}[\mathrm{Im}(C_1(\alpha _0))+p_2\mathrm{Im}(\lambda '(\alpha _0))] = 0.010032>0. \end{aligned}$$

**II.** Single-cell system for clock gene model

When $c=0$, system (44) is decoupled into

54 $$\begin{aligned} \left\{ \begin{aligned} \displaystyle {\dot{x}}_1&= \displaystyle \frac{\nu _1 k_1^n}{k_1^n + x_3^n} - \frac{\nu _2 x_1}{k_2 +x_1} \\ \displaystyle {\dot{x}}_2&= \displaystyle \nu _3 x_1 -\frac{\nu _4 x_2}{k_4 +x_2} -\nu _5 x_2 \\ \displaystyle {\dot{x}}_3&= \displaystyle \nu _5 x_2 -\frac{\nu _6 x_3}{k_6 +x_3} \\ \displaystyle {\dot{x}}_4&= \displaystyle \frac{\nu _7 k_7^h}{k_7^h + x_3^h} - \frac{\nu _8 x_4}{k_8 +x_4},\\ \end{aligned} \right. \end{aligned}$$

and

55 $$\begin{aligned} \left\{ \begin{aligned} \displaystyle {\dot{y}}_1&= \displaystyle \sigma \left( \frac{\nu _1 k_1^n}{k_1^n + y_3^n} - \frac{\nu _2 y_1}{k_2 +y_1}\right) \\ \displaystyle {\dot{y}}_2&= \displaystyle \sigma \left( \nu _3 y_1 -\frac{\nu _4 y_2}{k_4 +y_2} -\nu _5 y_2 \right) \\ \displaystyle {\dot{y}}_3&= \displaystyle \sigma \left( \nu _5 y_2 -\frac{\nu _6 y_3}{k_6 +y_3}\right) \\ \displaystyle {\dot{y}}_4&= \displaystyle \sigma \left( \frac{\nu _7 k_7^h}{k_7^h + y_3^h} - \frac{\nu _8 y_4}{k_8 +y_4}\right) . \\ \end{aligned} \right. \end{aligned}$$

First, we discuss the existence of positive equilibrium.

### Proposition 3

Assume $\nu _{8}>\nu _{7}$. For any fixed integers $n \ge 1$ and $h \ge 1$, there exists a unique positive equilibrium $\bar{{\mathbf{x}}}=({\bar{x}}_{1},{\bar{x}}_{2},{\bar{x}}_{3},{\bar{x}}_{4})$ for system (54), where

$$\begin{aligned} {\bar{x}}_{1}= & {} \frac{\nu _{6}{\bar{x}}_{3}[k_{6}(\nu _{4}+k_{4}\nu _{5})+(\nu _{4}+k_{4}\nu _{5}+\nu _{6}){\bar{x}}_{3}]}{\nu _{3}(k_{6}+{\bar{x}}_{3})[\nu _{6}{\bar{x}}_{3}+ k_{4}\nu _{5}(k_{6}+{\bar{x}}_{3})]},\\ {\bar{x}}_{2}= & {} \frac{\nu _{6}{\bar{x}}_{3}}{\nu _{5}(k_{6}+{\bar{x}}_{3})},\\ {\bar{x}}_{4}= & {} \frac{k_{7}^{h}k_{8}\nu _{7}}{k_{7}^{h}(\nu _{8}-\nu _{7})+\nu _{8}{\bar{x}}_{3}^{h}}, \end{aligned}$$

and ${\bar{x}}_{3}$ is the unique solution to the equation $P_{L}(\xi )=P_{R}(\xi )$, with

$$\begin{aligned} P_{L}(\xi )&:=\frac{\nu _{1}k_{1}^{n}}{k_{1}^{n}+\xi ^{n}}, \\ P_{R}(\xi )&:= \{[\nu _{2}\nu _{6}(\nu _{4}+k_{4}\nu _{5}+\nu _{6})]\xi ^{2}+k_{6}\nu _{2}\nu _{6}(\nu _{4}+k_{4}\nu _{5})\xi \} \\&\quad \cdot \{[k_{2}\nu _{3}(k_{4}\nu _{5}+\nu _{6})+\nu _{6}(\nu _{4}+k_{4}\nu _{5}+\nu _{6})]\xi ^{2} \\&\quad +[k_{6}(\nu _{6}(\nu _{4}+k_{4}\nu _{5})+k_{2}\nu _{3}(2k_{4}\nu _{5}+\nu _{6}))]\xi +k_{2}k_{4}k_{6}^{2}\nu _{3}\nu _{5}\}^{-1}. \end{aligned}$$

### Proof

$({\bar{x}}_{1},{\bar{x}}_{2},{\bar{x}}_{3},{\bar{x}}_{4})$ is an equilibrium of (54) if and only if $(x_1, x_2, x_3, x_4)=({\bar{x}}_{1}, {\bar{x}}_{2}, {\bar{x}}_{3}, {\bar{x}}_{4})$ satisfies

$$\begin{aligned} \left\{ \begin{array}{l} \displaystyle \frac{\nu _1 k_1^n}{k_1^n + x_3^n} - \frac{\nu _2 x_1}{k_2 +x_1} = 0 \\ \displaystyle \nu _3 x_1 -\frac{\nu _4 x_2}{k_4 +x_2} -\nu _5 x_2 = 0 \\ \displaystyle \nu _5 x_2 -\frac{\nu _6 x_3}{k_6 +x_3} = 0 \\ \displaystyle \frac{\nu _7 k_7^h}{k_7^h + x_3^h} - \frac{\nu _8 x_4}{k_8 +x_4} = 0. \end{array} \right. \end{aligned}$$

Accordingly,

$$\begin{aligned} {\bar{x}}_{1}= & {} \frac{\nu _{6}{\bar{x}}_{3}[k_{6}(\nu _{4}+k_{4}\nu _{5})+(\nu _{4}+k_{4}\nu _{5}+\nu _{6}){\bar{x}}_{3}]}{\nu _{3}(k_{6}+ {\bar{x}}_{3})[\nu _{6}{\bar{x}}_{3}+k_{4}\nu _{5}(k_{6}+{\bar{x}}_{3})]},\\ {\bar{x}}_{2}= & {} \frac{\nu _{6}{\bar{x}}_{3}}{\nu _{5}(k_{6}+{\bar{x}}_{3})},\\ {\bar{x}}_{4}= & {} \frac{k_{7}^{h}k_{8}\nu _{7}}{k_{7}^{h}(\nu _{8}-\nu _{7})+\nu _{8}{\bar{x}}_{3}^{h}}, \end{aligned}$$

and ${\bar{x}}_{3}$ satisfies $P_{L}(\xi )=P_{R}(\xi )$. Observe that there exists exactly one positive solution to $P_{L}(\xi )=P_{R}(\xi )$, due to $\nu _{8}>\nu _{7}$, and

$$\begin{aligned}&P_{R}(0)=0,~ P_{R}'(\xi )>0, \text{ for } \text{ all } \xi> 0,\\&P_{L}(0)>0,~ P_{L}'(\xi )<0, \text{ for } \text{ all } \xi > 0, \end{aligned}$$

and

$$\begin{aligned} \lim _{\xi \rightarrow \infty }P_{R}(\xi )=\frac{\nu _{2}\nu _{6}(\nu _{4}+k_{4}\nu _{5}+\nu _{6})}{k_{2}\nu _{3}(k_{4}\nu _{5}+\nu _{6})+\nu _{6}(\nu _{4}+k_{4}\nu _{5}+\nu _{6})}>0, ~\lim _{\xi \rightarrow \infty }P_{L}(\xi )=0. \end{aligned}$$

$$\square $$

We now take $\nu _1$ as the bifurcation parameter and employ Theorem 1 to locate the bifurcation values and confirm the existence of periodic solutions bifurcating from $\bar{{\mathbf{x}}}$. The Jacobian matrix associated with the linearization of system (55) at $\bar{{\mathbf{x}}}$ is

$$\begin{aligned} J(\bar{{\mathbf{x}}};\nu _1)=\left[ \begin{array}{cccc} ~- d_1 &{} 0 &{} - \nu _1\gamma _1 &{} 0~ \\ ~\nu _3 &{} - d_2 &{} 0 &{} 0~ \\ ~0 &{} \nu _5 &{} - d_3 &{} 0~ \\ 0 &{} 0 &{} -\gamma _3 &{} - d_4 \end{array}\right] , \end{aligned}$$

where

$$\begin{aligned}&d_1:=\frac{\nu _2k_2}{(k_2+{\bar{x}}_1)^2},~d_2:=\frac{\nu _4k_4}{(k_4+{\bar{x}}_2)^2}+\nu _5, ~d_3:=\frac{\nu _6k_6}{(k_6+{\bar{x}}_3)^2},~d_4:=\frac{\nu _8k_8}{(k_8+{\bar{x}}_4)^4},\\&\gamma _1 :=\frac{k_1^n n{\bar{x}}_3^{n-1}}{(k_1^n + {\bar{x}}_3^n)^2},~\gamma _3 :=\frac{\nu _7 k_7^h h{\bar{x}}_3^{h-1}}{(k_7^h + {\bar{x}}_3^h)^2}. \end{aligned}$$

The characteristic polynomial $\varDelta (\lambda ):=\mathrm{det}(\lambda I-J(\bar{{\mathbf{x}}};\nu _1))$ can be factored as

56 $$\begin{aligned} \varDelta (\lambda ) = \varDelta _{1}(\lambda )\cdot \varDelta _{2}(\lambda ), \end{aligned}$$

where

$$\begin{aligned}&\varDelta _{1}(\lambda ) := \lambda + d_4, \\&\varDelta _{2}(\lambda ) := \lambda ^3 + b_1\lambda ^2 + b_2\lambda + b_3, \\&b_1 := d_1+d_2+d_3,~b_2 := d_1d_2 + d_1d_3 + d_2d_3,~b_3 := d_1d_2d_3 + \nu _1\nu _3\nu _5 \gamma _1. \end{aligned}$$

Note that $$ b_1>0$$ and $$ b_3>0$$. Thus, applying (10) to $$\varDelta _{2}(\lambda )=0$$, we conclude that $$\varDelta (\lambda )$$ has a pair of purely imaginary roots and two negative roots if and only if $$ b_1 b_2- b_3=0$$. Our approach is to take $$\nu _1$$ as the bifurcation parameter and allow it to vary, while fix the other parameters at suitable values. From the equation $$ b_1(\nu _1^\diamond ) b_2(\nu _1^\diamond )- b_3(\nu _1^\diamond )=0$$, we find

57 $$\begin{aligned} \nu _1^\diamond = \frac{(d_1+d_2)(d_1+d_3)(d_2+d_3)}{\nu _3\nu _5\gamma _1} >0. \end{aligned}$$

From (57), we see that $\nu _1^\diamond$ can be expressed by ${\bar{x}}_1, {\bar{x}}_2$, ${\bar{x}}_3$, and hence by ${\bar{x}}_3$ and the other parameters. We substitute $\nu _1=\nu _1^\diamond$ by this expression into the stationary equations to solve for ${\bar{x}}_3$ numerically. If such solution ${\bar{x}}_3>0$ exists, then we can find ${\bar{x}}_1, {\bar{x}}_2$, ${\bar{x}}_4$ from (3). The crossing condition can also be examined numerically. By substituting $\nu _1=\nu _1^\diamond$ in (57) into $\varDelta _{2}(\lambda )$, we can also compute its purely imaginary roots $\pm i \omega _0$. From Theorem 1, let us summarize:

### Theorem 5

Assume that the equilibrium of system (54) exists at the Hopf bifurcation value $\nu _1=\nu _1^\diamond$, and the crossing condition holds. Then, the system undergoes a Hopf bifurcation at ${\mathbf{x}}=\bar{\mathbf{x}}$ and $\nu _1 = \nu _1^\diamond$, and a small-amplitude periodic solution near $\bar{\mathbf{x}}$ emerges as $\nu _1<\nu _1^\diamond$ or $\nu _1>\nu _1^\diamond$ and $\nu _1$ is close to $\nu _1^\diamond$, with frequency approximately

$$\begin{aligned} \omega _0 = \sqrt{d_1d_2 + d_1d_3 + d_2d_3} >0. \end{aligned}$$

Note that system (55) undergoes a Hopf bifurcation at the same ${\mathbf{x}}=\bar{\mathbf{x}}$ and $\nu _1 = \nu _1^\diamond$, with frequency about $\sigma \omega _0$ for the emergent periodic solution.

**Example S.** Consider system (54) with the following parameter values

$$\begin{aligned}&k_1= 0.103,~ k_2= 9.916,~ k_4= 0.182,~ k_6= 0.302,~ k_7= 1.87,~ k_8= 0.377, \\&\nu _2= 0.963,~ \nu _3= 45.172,~ \nu _4= 1.447,~\nu _5= 0.15,~\nu _6= 0.147,~\nu _7= 1.912 \\&\nu _8= 2.315, ~n=h=2. \end{aligned}$$

These values either coincide with the ones in the example in Uriu et al. (2010) or fall within the designated ranges therein. We look for a value $\nu _1^\diamond$ which satisfies (57). First, we express $\nu _1^\diamond$ in (57) in terms of $\gamma _1$ which in turn is a function of ${\bar{x}}_3$. We substitute $\nu _1=\nu _1^\diamond$ into system (54) to find the equilibrium. After computation, the equilibrium is $\bar{{\mathbf{x}}}=({\bar{x}}_1,{\bar{x}}_2,{\bar{x}}_3,{\bar{x}}_4)$, with ${\bar{x}}_1 \approx 0.026935$, ${\bar{x}}_2 \approx 0.629151$, ${\bar{x}}_3 \approx 0.541554$, ${\bar{x}}_4 \approx 1.207095$. With this $\bar{{\mathbf{x}}}$, we then compute to obtain the bifurcation value $\nu _1^\diamond \approx 0.074726$. Therefore, with further computation, we can confirm that a periodic solution emerges at $\nu _1>\nu _1^\diamond$ and $\nu _1$ close to $\nu _1^\diamond$, with frequency approximately $\omega _0 = 0.305785$. We illustrate such result by computing numerically system (54) with $\nu _1 = 0.076$ and the other parameter values as above. We observe the periodic solution with frequency 0.305142, shown in Fig. 30.

The Jacobian matrix associated with the linearization of system (44) at $\overline{{\mathbb {X}}}$ is

$$\begin{aligned} J(\overline{{\mathbb {X}}};\nu _1) := \left[ \begin{array}{cccccccc} ~-d_1 &{} 0 &{} -(\nu _1+\nu _c{\bar{y}}_4)\gamma _1 &{} 0 &{} 0 &{} 0 &{} 0 &{} \gamma _2~ \\ ~\nu _3 &{} -d_2 &{} 0 &{} 0 &{} 0 &{} 0 &{} 0 &{} 0~ \\ ~0 &{} \nu _5 &{} -d_3 &{} 0 &{} 0 &{} 0 &{} 0 &{} 0~ \\ ~0 &{} 0 &{} -\gamma _3 &{} -d_4 &{} 0 &{} 0 &{} 0 &{} 0~ \\ ~0 &{} 0 &{} 0 &{} \gamma _5 &{} -\sigma d_5 &{} 0 &{} \displaystyle -\sigma \left( \nu _1+\frac{\nu _c}{\sigma }{\bar{x}}_4\right) \gamma _4 &{} 0~\\ ~0 &{} 0 &{} 0 &{} 0 &{} \sigma \nu _3 &{} -\sigma d_6 &{} 0 &{} 0~\\ ~0 &{} 0 &{} 0 &{} 0 &{} 0 &{} \sigma \nu _5 &{} -\sigma d_7 &{} 0~\\ ~0 &{} 0 &{} 0 &{} 0 &{} 0 &{} 0 &{} -\sigma \gamma _6 &{} -\sigma d_8~\\ \end{array}\right] , \end{aligned}$$

where

$$\begin{aligned}&d_1:=\frac{\nu _2k_2}{(k_2+{\bar{x}}_1)^2},~d_2:=\frac{\nu _4k_4}{(k_4+{\bar{x}}_2)^2}+\nu _5, ~d_3:=\frac{\nu _6k_6}{(k_6+{\bar{x}}_3)^2},~d_4:=\frac{\nu _8k_8}{(k_8+{\bar{x}}_4)^2},\\&d_5:=\frac{\nu _2k_2}{(k_2+{\bar{y}}_1)^2},~d_6:=\frac{\nu _4k_4}{(k_4+{\bar{y}}_2)^2}+\nu _5, ~d_7:=\frac{\nu _6k_6}{(k_6+{\bar{y}}_3)^2},~d_8:=\frac{\nu _8k_8}{(k_8+{\bar{y}}_4)^2},\\&\gamma _1 :=\frac{k_1^n n{\bar{x}}_3^{n-1}}{(k_1^n + {\bar{x}}_3^n)^2},~\gamma _2 :=\frac{\nu _ck_1^n}{k_1^n+{\bar{x}}_3^n},~\gamma _3 :=\frac{\nu _7k_7^h h{\bar{x}}_3^{h-1}}{(k_7^h + {\bar{x}}_3^h)^2},\\&\gamma _4 :=\frac{k_1^n n{\bar{y}}_3^{n-1}}{(k_1^n + {\bar{y}}_3^n)^2},~\gamma _5 :=\frac{\nu _ck_1^n}{k_1^n+{\bar{y}}_3^n},~\gamma _6 :=\frac{\nu _7k_7^h h{\bar{y}}_3^{h-1}}{(k_7^h + {\bar{y}}_3^h)^2}. \end{aligned}$$
